# CBioProfiler: A Web and Standalone Pipeline for Cancer Biomarker and Subtype Characterization

**DOI:** 10.1093/gpbjnl/qzae045

**Published:** 2024-06-12

**Authors:** Xiaoping Liu, Zisong Wang, Hongjie Shi, Sheng Li, Xinghuan Wang

**Affiliations:** Department of Urology, Zhongnan Hospital of Wuhan University, Wuhan 430071, China; Department of Pathology, Zhongnan Hospital of Wuhan University, Wuhan 430071, China; Cancer Precision Diagnosis and Treatment and Translational Medicine Hubei Engineering Research Center, Zhongnan Hospital of Wuhan University, Wuhan 430071, China; Department of Urology, Zhongnan Hospital of Wuhan University, Wuhan 430071, China; Cancer Precision Diagnosis and Treatment and Translational Medicine Hubei Engineering Research Center, Zhongnan Hospital of Wuhan University, Wuhan 430071, China; Department of Biological Repositories, Zhongnan Hospital of Wuhan University, Wuhan 430071, China; Department of Urology, Zhongnan Hospital of Wuhan University, Wuhan 430071, China; Cancer Precision Diagnosis and Treatment and Translational Medicine Hubei Engineering Research Center, Zhongnan Hospital of Wuhan University, Wuhan 430071, China; Department of Biological Repositories, Zhongnan Hospital of Wuhan University, Wuhan 430071, China; Department of Urology, Zhongnan Hospital of Wuhan University, Wuhan 430071, China; Cancer Precision Diagnosis and Treatment and Translational Medicine Hubei Engineering Research Center, Zhongnan Hospital of Wuhan University, Wuhan 430071, China; Department of Biological Repositories, Zhongnan Hospital of Wuhan University, Wuhan 430071, China

**Keywords:** CBioProfiler, Cancer biomarker, Cancer subtype, Machine learning, Web app

## Abstract

Cancer is a leading cause of death worldwide, and the identification of biomarkers and subtypes that can predict the long-term survival of cancer patients is essential for their risk stratification, treatment, and prognosis. However, there are currently no standardized tools for exploring cancer biomarkers or subtypes. In this study, we introduced Cancer Biomarker and subtype Profiler (CBioProfiler), a web server and standalone application that includes two pipelines for analyzing cancer biomarkers and subtypes. The cancer biomarker pipeline consists of five modules for identifying and annotating cancer survival-related biomarkers using multiple survival-related machine learning algorithms. The cancer subtype pipeline includes three modules for data preprocessing, subtype identification using multiple unsupervised machine learning methods, and subtype evaluation and validation. CBioProfiler also includes CuratedCancerPrognosisData, a novel R package that integrates reviewed and curated gene expression and clinical data from 268 studies. These studies cover 43 common blood and solid tumors and draw upon 47,686 clinical samples. The web server is available at https://www.cbioprofiler.com/ and https://cbioprofiler.znhospital.cn/CBioProfiler/, and the standalone app and source code can be found at https://github.com/liuxiaoping2020/CBioProfiler.

## Introduction

In recent years, with the advancement of high-throughput sequencing technologies including DNA sequencing, RNA sequencing, microarray, single-cell sequencing, and their wide application in medical research and clinical practice, a large number of gene expression profiling studies of cancer patients have been published [1]. The gene expression data of these studies and the clinical data of the corresponding patients are mostly stored in public databases such as Gene Expression Omnibus (GEO) (https://www.ncbi.nlm.nih.gov/geo/), The Cancer Genome Atlas (TCGA) (https://portal.gdc.cancer.gov/), ArrayExpress (https://www.ebi.ac.uk/arrayexpress/), Therapeutically Applicable Research To Generate Effective Treatments (TARGET) (https://ocg.cancer.gov/programs/target), International Cancer Genome Consortium (ICGC) (https://dcc.icgc.org/), and Chinese Glioma Genome Atlas (CGGA) (http://www.cgga.org.cn/). Some of them are uploaded as research supplements on the official website of the journal or related research institutions. However, due to variations in data storage, preprocessing, and operational interfaces across databases, as well as significant differences in data collection, preprocessing, format, and documentation between clinical and gene expression data in each study, individuals aiming to fully and effectively utilize these high-throughput data for research and clinical practice guidance encounter substantial obstacles.

Due to the population aging and changes in people’s lifestyles, malignant tumors have emerged as one of the primary threats to human health and longevity. [2]. Therefore, developing and validating of novel tumor biomarkers and subtypes that can be used for tumor diagnosis, risk stratification, and prognosis is crucial for the early detection and personalized treatment of tumors. With the advancement of artificial intelligence, more and more machine learning strategies have been applied to the screening and validation of biomarkers and subtypes for cancer patients [3,4]. However, due to the lack of a unified, standardized, and rigorous model as well as variable selection processes, the reliability of relevant biomarkers and subtypes is questionable.

**Figure 1:**
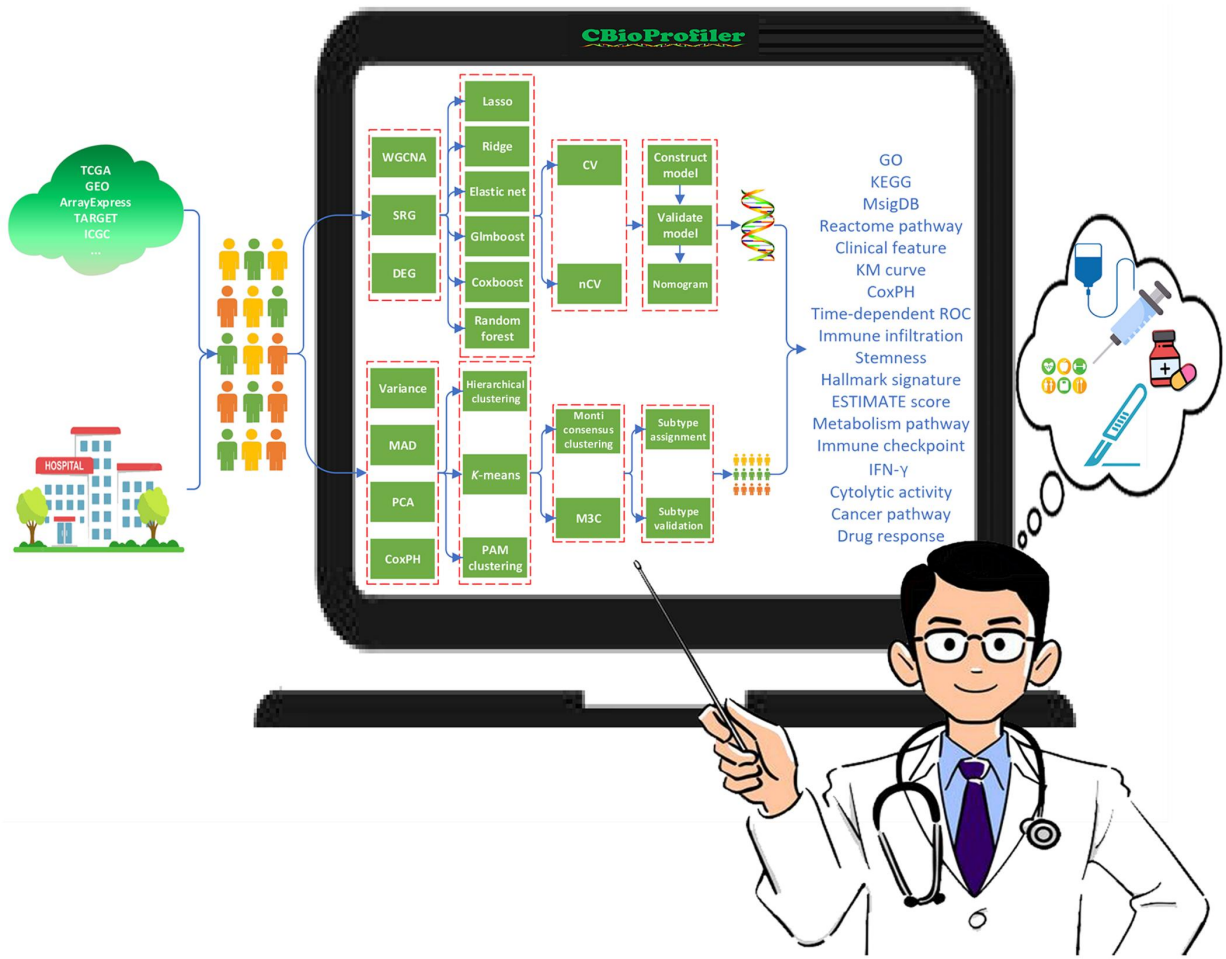
Overview of CBioProfiler The main pipeline of CBioProfiler includes two main pipelines: cancer biomarker pipeline and cancer subtype pipeline. The cancer biomarker pipeline includes five modules: (1) dimensionality reduction using three methods of WGCNA, SRG, DEG analysis; (2) benchmark experiment with six machine learning learners (Lasso, ridge, elastic net, Glmboost, Coxboost, and random forest) using CV and nCV; (3) prediction model construction and nomogram; (4) clinical annotation using a variety of clinical approaches; and (5) biological annotation using ORA and GSEA. The subtype pipeline includes three modules: (1) data preprocessing (feature selection based on variance, MAD, CoxPH model, and PCA, (2) subtype identification (integration of multiple unsupervised machine learning methods (hierarchical clustering, *K*-means, PAM clustering, *etc*.) using two popular consensus clustering methods (Monti consensus clustering and M3C), (3) subtype evaluation and validation. TCGA, The Cancer Genome Atlas; GEO, Gene Expression Omnibus; TARGET, Therapeutically Applicable Research to Generate Effective Treatments; ICGC, International Cancer Genome Consortium; WGCNA, weighted gene co-expression network analysis; SRG, survival-related gene; DEG, differentially expressed gene; CV, cross-validation; nCV, nested cross-validation; ORA, over-representation analysis; GSEA, gene set enrichment analysis; MAD, median absolute deviation; PCA, principal component analysis; PAM, partitioning around medoids; CBioProfiler, Cancer Biomarker and subtype Profiler; M3C, Monte Carlo reference-based consensus clustering; CoxPH, Cox proportional hazards; Lasso, least absolute shrinkage and selection operator; GO, Gene Ontology; KEGG, Kyoto Encyclopedia of Genes and Genomes; MSigDB, Molecular Signatures Database; KM, Kaplan–Meier; ROC, receiver operating characteristic curve; IFN-γ, interferon-gamma; ESTIMATE, estimation of stromal and immune cells in malignant tumor tissues using expression data.

Thus, in the present study, we developed and introduced Cancer Biomarker and subtype Profiler (CBioProfiler), a web server and standalone pipeline that reviewed, curated, and integrated the gene expression data and corresponding clinical data of 47,686 samples from 268 gene expression studies of 43 common blood and solid tumors, for screening, validation, and annotation of cancer biomarkers and subtypes from molecular level to clinical settings (**Figure 1**) (https://github.com/liuxiaoping2020/CBioProfiler_tutorial/blob/main/CBioProfiler_tutorial.pdf).

## Method

### Data collection and curation

We searched and downloaded gene expression profiling data of tumor patients from GEO, TCGA, ICGC, ArrayExpress, TARGET, CGGA, and other public databases or websites. The following criteria were used to include datasets in our research: (1) the research subjects were cancer patients; (2) the dataset contained gene expression profiling data; (3) the dataset reported at least one type of follow-up information, such as overall survival (OS), progression-free survival (PFS), relapse-free survival (RFS), disease-free survival (DFS), or distant metastasis-free survival (DMFS); (4) the sample size of the dataset was greater than 20. For data from GEO and ArrayExpress, R package GEOquery (v2.56.0) and ArrayExpress (v1.48.0) were used to download them, respectively. If the raw data were available, robust multichip average (RMA) method [5] was used to normalize the raw data using R package affy (v1.66.0) or oligo (v1.52.1); otherwise, the normalized data were used. For data from TCGA and Multiple Myeloma Research Foundation’s Relating Clinical Outcomes in Multiple Myeloma to Personal Assessment of Genetic Profile (MMRF-CoMMpass) [6], we downloaded the RNA sequencing (RNA-seq) count data from Genomic Data Commons (GDC) and transformed them to transcripts per million (TPM) values using R package TCGAbiolinks (v1.14.0). For data from TARGET, ICGC, and CGGA, normalized data were downloaded and used indirectly. Annotation files provided by the submitters were used to annotate the gene expression profiling data. When multiple probes match to the same gene, we chose the most variant probe, and when multiple genes correspond to the same probe, we dropped them since unspecific annotation. Clinical data were uniformly reformatted and curated using in-house R scripts for each dataset, and independent double-checking was conducted by investigators to ensure the accuracy of the curation. The workflow of the curation is summarized in Figure S1.

### Cancer biomarker pipeline

#### Dimensionality reduction

CBioProfiler uses three of the most commonly used bioinformatics methods to reduce the dimensionality of data: (1) weighted gene co-expression network analysis (WGCNA) [7]; (2) survival-related genes (SRGs) [8]; (3) differentially expressed genes (DEGs) [9]. WGCNA includes three steps: (1) Euclidean distance-based sample network is used to filter outlying samples, (2) a weighted gene co-expression network is constructed to identify gene modules whose expression profiles are similar based on adjacency matrix and appropriate soft threshold, and (3) correlations between gene modules and clinical features are calculated. Empirical Bayesian method is used to perform DEG analysis using R package limma (v3.44.3) [9]. SRG is implemented based on univariate Cox proportional hazards (CoxPH) regression model using R package survival (v3.2-3).

#### Survival learners

For benchmark experiment, CBioProfiler includes six embedded machine learning algorithms, including least absolute shrinkage and selection operator (Lasso) [10], ridge [11], elastic net [12], Glmboost [13], Coxboost [14], and random forest [15] for survival analysis. Lasso, proposed by Robert Tibshirani in 1996, obtains a more refined model by constructing a L1 norm penalty function, which forces the sum of absolute values of coefficients to be less than a certain fixed value and sets some regression coefficients to zero. Therefore, it is a regression analysis method that performs feature selection and regularization at the same time, and aims to enhance the prediction accuracy and interpretability of statistical models [10]. Ridge regression is similar to linear regression, both of which are to solve the over-fitting problem of standard linear regression. The difference is that ridge regression adds the L2 norm penalty [11]. Elastic net integrates the L1 norm and the L2 norm, which makes it having both the variable selection and regularization advantages of Lasso and ridge regression [12]. Glmboost fits generalized linear model and conducts variable selection based on component-wise boosting [13]. Unlike Glmboost, Coxboost fits a CoxPH by component-wise likelihood-based boosting [14]. For feature selection, the aforementioned five survival learners retain features with non-zero coefficients. Random forest, being an ensemble model, is originally available only for regression and classification tasks. However, randomForestSRC extends the capabilities of random forest to survival analysis, performing variable selection based on maximal subtree information [15]. Parameter sets for the survival learners are summarized in Table S1.

#### Benchmark experiment

Benchmark experiment is supported by the R package mlr (v2.18.0). To train and validate the survival learners, we utilize cross-validation (CV) and nested cross-validation (nCV) methodologies as part of the benchmark experiment. During CV, the entire dataset is randomly split into a training set and a test set, and then *k*-fold CV is applied to the training set as follows: (1) divide the training set into equal *K* folds; (2) use the first fold as inner test set, and the rest as inner training set; (3) train the model and calculate the C-index of the model on the inner test set; (4) use a different fold as inner test set each time, and repeat steps (2) and (3) *K* times; and (5) apply the best model to test set and external independent validation cohort. CV is designed for model selection. When the best model is selected, bootstrap resampling can be used to evaluate and compare the performances of different survival learners. The workflow of CV is summarized in Figure S2.

With respect to nCV, the entire dataset is divided into *N* outer folds, and then each outer fold is divided into a training set and a test set. Then, the main steps of nCV can be summarized as follows: (1) divide the training set into equal *K* folds; (2) use the first fold as inner test set, and the rest as inner training set; (3) train the model and calculate the C-index of the model on the inner test set; (4) use a different fold as inner test set each time, and repeat steps (2) and (3) *K* times; (5) apply the best model to outer fold test set; (6) select the best outer model features and parameters and train on the whole dataset to get final model; (7) if the users have divided the whole dataset into two parts, one is for training nCV, the other is for validation, then they can validate the final model on the validation part and external validation cohort; otherwise, they can validate the final model on external cohort. nCV utilizes multi-layer CV to implement model selection. The workflow is summarized in Figure S3.

#### Prediction model

After the benchmark experiments are completed, CBioProfiler could compute the fitted relative risk of patients in the training set, test set, and external validation set. Lasso, ridge, elastic net, Glmboost, and Coxboost calculate the relative risk using the predict function, while randomForestSRC uses the sum of cumulative hazard function (CHF). Based on the relative risk scores, prediction models for specific survival endpoints are constructed and validated using time-dependent receiver operating characteristic curve (ROC) [implemented with R package survivalROC (v1.0.3)], Kaplan–Meier curve [implemented and visualized using R packages survival (v3.2-3) and survminer (v0.4.8)], and CoxPH. Moreover, CBioProfiler allows to construct nomogram that includes the relative risk score and other clinical features, which helps researchers and physicians predict the survival probability of cancer patients. The nomogram can be internally and externally validated and calibrated based on bootstrap resampling and calibration analysis.

#### Clinical annotation

To help users investigate the clinical relevance of the biomarkers that they identified, CBioProfiler allows users to analyze the correlation between a given biomarker and clinical features (“Correlation with clinical features” module), characterize the prognostication significance of given biomarkers (“Kaplan–Meier curve” module, “CoxPH model” module, and “Time-dependent ROC” module), identify genes correlated with given biomarkers (“Most correlated genes” module and “Correlation with specific gene” module), compare the expression levels of given biomarkers among different groups (“Gene expression in different groups” module), and investigate the relationship between given biomarkers and immune cell infiltration [16], cancer stemness score [17], estimation of stromal and immune cells in malignant tumor tissues using expression data (ESTIMATE) score [18], immune checkpoint [19], interferon-gamma (IFN-γ) score [20], cytolytic activity [21], cancer-related signaling pathway [22], metabolism pathway [23], hallmark signature [24], and drug response [25]. Spearman’s correlation and Pearson’s correlation are used for correlation analysis.

#### Biological annotation

Yu and his colleagues developed clusterProfiler (v3.18.1) [26], a very outstanding R package for gene functional annotation. CBioProfiler integrates some useful functions of clusterProfiler to annotate tumor biomarkers, allowing users to perform functional annotation of their biomarkers regarding Gene Ontology (GO), Kyoto Encyclopedia of Genes and Genomes (KEGG), molecular signatures database (MSigDB), and Reactome pathway based on over-representation analysis (ORA) [27,28] and gene set enrichment analysis (GSEA) [29].

### Cancer subtype pipeline

The subtype pipeline includes three modules: (1) data preprocessing, *i.e.*, feature selection based on variance, median absolute deviation (MAD), CoxPH model [30], and principal component analysis (PCA) [31]; (2) subtype identification, *i.e.*, integration of multiple unsupervised machine learning methods (*K*-means clustering [32], hierarchical clustering [33], partitioning around medoids (PAM) clustering [34], *etc.*) using two popular consensus clustering methods, ConsensusClusterPlus (v1.52.0) [35] and M3C (v1.10.0) [36]; (3) subtype evaluation and validation. In order to further clarify the biological and clinical significance of different subtypes, CBioProfiler also provides a variety of biological annotation modules (similar to biomarker modules). For group comparisons, *t*-test, analysis of variance (ANOVA), Kruskal–Wallis, and Wilcoxon test are available for use.

### Meta-analysis pipeline

CBioProfiler offers a meta-analysis module that helps researchers evaluate the effect of biomarkers on patient prognosis. This module utilizes the methods of Schwarzer et al. [37], which involves calculating the correlation between a particular gene and the survival time of patients in a specific cohort using a univariate CoxPH model. The module then performs meta-analysis based on the hazard ratio (HR) and its 95% confidence interval (CI) of the patients.

## Results

### Curated public gene expression data

**Figure 2:**
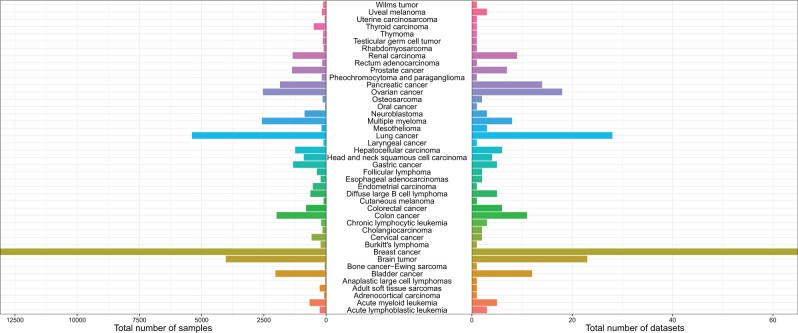
Total number of samples and datasets included in CuratedCancerPrognosisData

We reviewed, curated, normalized, and integrated the gene expression data and corresponding clinical data of 43 common blood and solid tumors from GEO, TCGA, ICGC, TARGET, ArrayExpress, and other public databases. These public data from 47,686 clinical samples of 268 gene expression studies (**Figure 2**; Table S2) (https://liuxiaoping2020.github.io/CBioProfilerDatasource/) were integrated to build an R package “CuratedCancerPrognosisData”, and the associated source code was deposited at https://zenodo.org/records/7481234.

### Compared with other similar online tools or standalone apps

As shown in **Table 1**, compared with other similar tools, CBioProfiler demonstrates significant superiority across various aspects, including: (1) CBioProfiler encompasses the broadest range of disease types and samples, and provides a personalized data submission interface for researchers to analyze their own data; (2) CBioProfiler covers the most functional modules; (3) CBioProfiler offers both an online version and a standalone local app version, catering to diverse user preferences and facilitating seamless access to its functionalities.

**Table 1:** Comparison of CBioProfiler with other similar online tools or standalone apps

		CBioProfiler	GEPIA/GEPIA2	PROGgeneV2	SurvExpress	KM Plotter	UALCAN	GSCALite	CAS-viewer	OncoLnc	CaPSSA	LOGpc	PRECOG	cBioPortal
Data	Sample size	47,686	10,588	28,503	39,325	14,912	7233	10,558	10,558	8616	10,206	31,310	19,168	
	No. of disease types	45	33	27	26	21	33	33	33	21	27	27	39	32
	No. of datasets	268	33	193	225	45	35	63	63	21	28	209	165	> 100
	Availability of curated data	Yes	No	No	No	No	No	No	No	No	No	No	Yes	Yes
	Normalized gene expression data	Yes	Yes	Yes	Yes	No	No	No	No	No	No	No	Yes	Yes
	Customized data input and analysis	Yes	Yes	No	No	No	No	No	No	No	Yes	No	No	No
Feature	Dimensionality reduction	Yes	Yes	No	No	No	No	No	No	No	No	No	No	No
	CV or nCV	Yes	No	No	No	No	No	No	No	No	No	No	No	No
	Construction and evaluation of predictive model	Yes	No	No	No	No	No	No	No	No	Yes	No	No	No
	Nomogram	Yes	No	No	No	No	No	No	No	No	No	No	No	No
	Cancer subtype identification and validation	Yes	No	No	No	No	No	No	No	No	No	No	No	No
	Cancer subtype annotation	Yes	No	No	No	No	No	No	No	No	No	No	No	No
	KM curve	Yes	Yes	Yes	Yes	Yes	Yes	Yes	Yes	Yes	Yes	Yes	Yes	Yes
	Optimal cutoff	Yes	No	No	No	No	No	No	No	No	No	No	No	No
	Univariate CoxPH	Yes	Yes	No	No	No	No	No	No	No	No	No	No	No
	Multivariate CoxPH	Yes	No	Yes	No	No	No	No	No	No	No	No	No	No
	Time-dependent ROC analysis	Yes	No	No	No	No	No	No	No	No	No	No	No	No
	Correlation with clinical features	Yes	No	No	No	No	No	No	No	No	Yes	No	No	No
	Differential expression analysis	Yes	Yes	No	No	No	Yes	Yes	Yes	No	Yes	No	No	No
	Correlation with other genes	Yes	Yes	No	No	No	No	No	No	No	No	No	No	No
	Immune cell infiltration analysis	Yes	Yes	No	No	No	No	No	No	No	No	No	No	No
	Stemness score	Yes	No	No	No	No	No	No	No	No	No	No	No	No
	ESTIMATE score	Yes	No	No	No	No	No	No	No	No	No	No	No	No
	Immune checkpoints	Yes	No	No	No	No	No	No	No	No	No	No	No	No
	INF-γ	Yes	No	No	No	No	No	No	No	No	No	No	No	No
	Cytolytic activity	Yes	No	No	No	No	No	No	No	No	No	No	No	No
	Cancer pathway	Yes	No	No	No	No	No	No	No	No	No	No	No	No
	Metabolism score	Yes	No	No	No	No	No	No	No	No	No	No	No	No
	Hallmark signature	Yes	No	No	No	No	No	No	No	No	No	No	No	No
	Drug response	Yes	No	No	No	No	No	No	No	No	No	No	No	No
	Multi-omics analysis	No	No	No	No	No	No	Yes	No	No	Yes	No	No	No
	Pan-cancer analysis	No	Yes	No	No	No	No	No	No	Yes	No	No	No	Yes
Enrichment analysis	GSEA	Yes	No	No	No	No	No	No	No	No	No	No	No	No
	ORA	Yes	No	No	No	No	No	No	No	No	No	No	No	No
	GO	Yes	No	No	No	No	No	No	No	No	No	No	No	No
	KEGG	Yes	No	No	No	No	No	No	No	No	No	No	No	No
	MsigDB	Yes	No	No	No	No	No	No	No	No	No	No	No	No
	Reactome pathway	Yes	No	No	No	No	No	No	No	No	No	No	No	No
Software	Log in required	No	No	No	No	No	No	No	No	No	No	No	No	No
	Web interface	Yes	Yes	Yes	Yes	Yes	Yes	Yes	Yes	Yes	Yes	Yes	Yes	Yes
	Standalone application	Yes	No	No	No	No	No	No	No	No	No	No	No	No
	Interactive results	Yes	No	No	No	No	No	No	No	No	No	No	No	No
	Availability of source code	Yes	No	No	No	No	No	No	No	No	No	No	No	No
	Result download	Yes	Yes	Yes	Yes	Yes	Yes	Yes	Yes	Yes	Yes	No	No	Yes

*Note*: CBioProfiler, Cancer Biomarker and subtype Profiler; ESTIMATE, estimation of stromal and immune cells in malignant tumor tissues using expression data; KM, Kaplan–Meier; CoxPH, Cox proportional hazards; ROC, receiver operating characteristic curve; GSEA, gene set enrichment analysis; ORA, over representation analysis; KEGG, Kyoto Encyclopedia of Genes and Genomes; MsigDB, Molecular Signatures Database; CV, cross-validation; nCV, nested cross-validation; INF-γ, interferon-gamma; GO, Gene Ontology.

### Case study: dimensionality reduction

**Figure 3:**
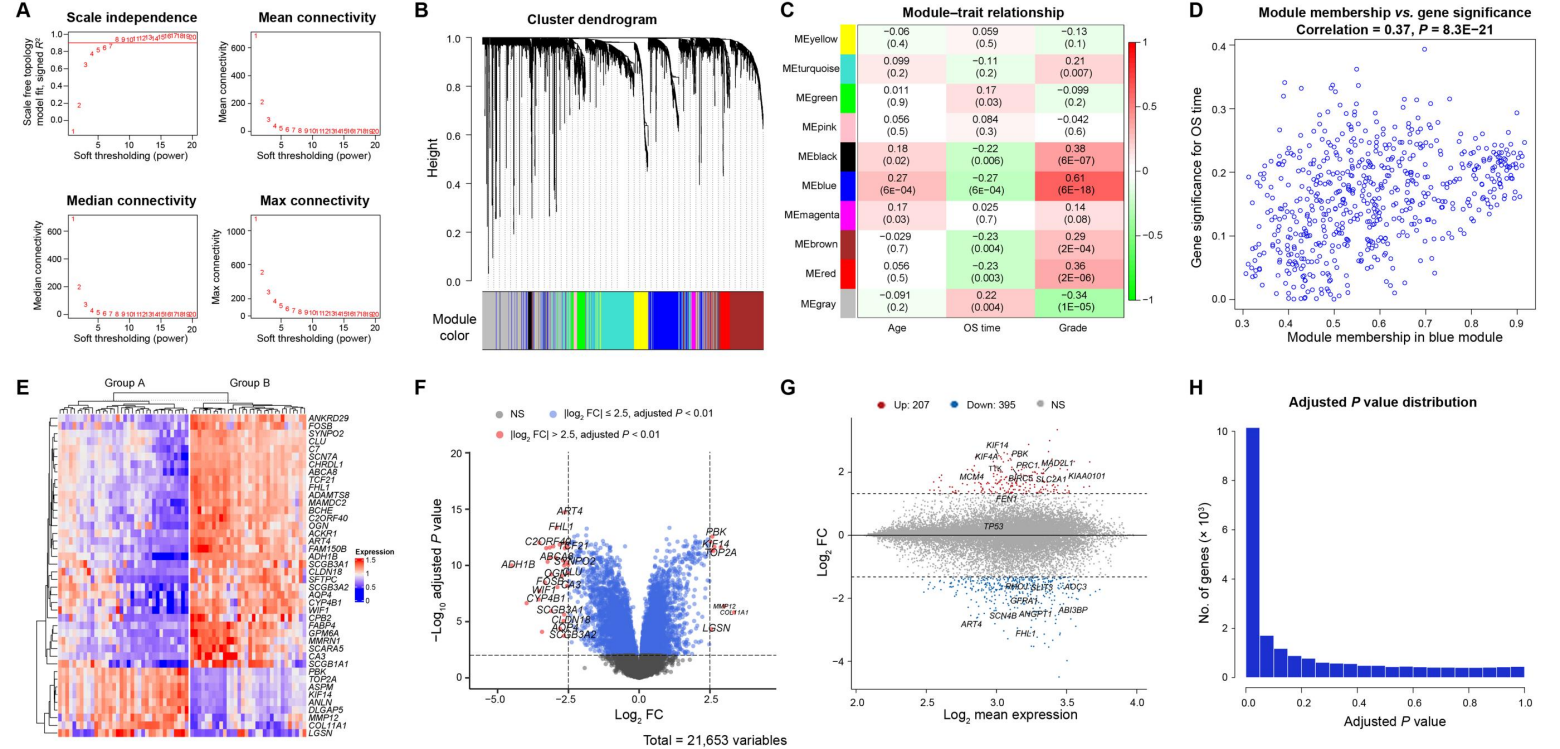
Main result output of WGCNA and DEG analysis **A**. Selection of soft-thresholding power. **B**. Modules detected by WGCNA. **C**. Heatmap showing module–trait relationships. Each cell reports the correlation (*P* value) resulting from correlating module eigengenes (rows) to traits (columns). **D**. Gene significance for grade *vs*. module membership in the blue module. **E**. Heatmap showing DEGs between group A and group B. **F**. Volcano plot showing DEGs between group A and group B. **G**. MA plot showing DEGs between group A and group B. **H**. Adjusting *P* plot of DEG analysis. OS, overall survival; FC, fold change; NS, no significance.

CBioProfiler enables three methods (WGCNA, SRG, and DEG) to conduct dimensionality reduction. WGCNA [38] is a biometric method that can cluster genes with similar expression patterns or functions into the same module, while unassigned genes are categorized into a gray module. Bladder cancer represents one of the most common types of malignant cancers of human genitourinary system. Kim et al. published a far-reaching bladder cancer gene expression study GSE13507, which evaluated the predictive effect of bladder cancer prognosis-related genes for patients [39]. In this example, we used the WGCNA to perform dimensionality reduction on GSE13507, and then screened genes that were closely related to the patients’ OS for subsequent studies. After Euclidean distance-based clustering, three samples were detected as outliers, and the remaining 162 samples were used to construct co-expression network (Figure S4). Then, according to the soft-thresholding power 8 (**Figure 3**A), we constructed a co-expression network, which clustered all genes into 10 modules (Figure 3B). Next, we analyzed the relationship between the gene modules and the clinical features of bladder cancer patients. As a result, “green” module was most positively relevant to the OS, while “blue” module was most negatively correlated with the OS of bladder cancer patients (Figure 3C and D). Finally, we could output the genes of any module or non-gray module for subsequent research.

CoxPH is often used clinically to evaluate the impact of clinical phenotypes on patient survival. Thus, we used the breast cancer gene expression study Molecular Taxonomy of Breast Cancer International Consortium (METABRIC) [40] to illustrate the use of univariate CoxPH for dimensionality reduction analysis. The METABRIC project introduced a novel risk stratification system for patients with breast cancer based on multi-omics high-throughput data. We performed univariate CoxPH to analyze the impact of a single gene on the OS of breast cancer patients. We included the top 60 genes that are closely related to the OS of patients (Table S3).

DEGs between biological groups are of great significance to clarify the biological significance of the groups. Therefore, it is also very recommended to screen DEGs for dimensionality reduction analysis. Okayama et al. conducted transcription profiling of 226 stage I and II lung adenocarcinomas, which clustered these lung adenocarcinomas into two groups (group A: patients that are mainly males, ever-smokers, and advanced stages; group B: patients that are mainly never-smokers and early stages) [41]. As shown in Figure 3E–H, there were 42 significant DEGs detected at adjusted *P* value < 0.01 and absolute log_2_ fold change (FC) > 2.5 between the two groups of lung adenocarcinomas. The DEGs were visualized using heatmap, volcano plot, MA plot, and adjusted *P* plot.

### Case study: building and validating prediction model and constructing associated nomogram

**Figure 4:**
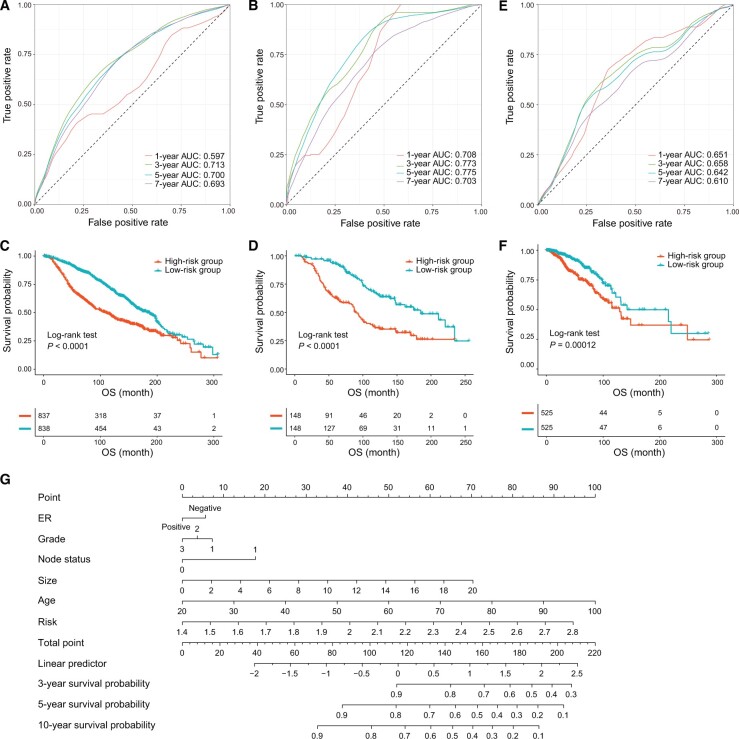
Construction, evaluation and translation of the prediction model **A**. AUCs of time-dependent ROC analysis at 1-, 3-, 5-, and 7-year time points in the training set. **B**. AUCs of time-dependent ROC analysis at 1-, 3-, 5-, and 7-year time points in the test set. **C**. Survival differences between the low-risk group and high-risk group in the training set. **D**. Survival differences between the low-risk group and high-risk group in the test set. **E**. AUCs of time-dependent ROC analysis at 1-, 3-, 5-, and 7-year time points in the validation set. **F**. Survival differences between the low-risk group and high-risk group in the validation set. **G**. Nomogram prediction of the 3-, 5-, and 10-year survival probabilities based on the CoxPH model that integrates the risk, ER status, tumor size, node status, age, and grade of breast cancer patients. AUC, area under the curve; ER, estrogen receptor.

To build a prediction model, METABRIC was treated as the discovery cohort, which was then randomly stratified into a training set and a test set according to a ratio of 0.85. The 60 genes selected by the CoxPH model were applied to train three machine learning learners (Lasso, elastic net, and Glmboost) to construct a prediction model based on 10-fold CV. The performances of these models were then evaluated and compared using 100 bootstraps. As shown in Figure S5, the performance of elastic net outperformed both Lasso and Glmboost. Thus, elastic net was selected to build the prediction model. Results of time-dependent ROC showed that the areas under the curves (AUCs) of the prediction model in the training set at 1-, 3-, 5-, and 7-year time points were 0.597, 0.713, 0.700, and 0.693, respectively (**Figure 4**A), while the corresponding AUCs in the test set were 0.708, 0.773, 0.775, and 0.703, respectively (Figure 4B). Kaplan–Meier plots showed that patients in the low-risk group had significant better OS compared to those in the high-risk group in both training set and test set (Figure 4C and D). Moreover, the risk score remained an independent prognostic factor after adjusting for other clinical features of breast cancer patients (Figure S6).

TCGA-BRCA [42] is an independent multi-omics study of breast cancer. Thus, we utilized it as an external validation cohort. Time-dependent ROC analysis showed that the AUCs of the prediction model in the validation cohort were 0.651, 0.658, 0.642, and 0.610 at 1-, 3-, 5-, and 7-year time points, respectively (Figure 4E), and the risk score could also stratify patients into different risk groups (Figure 4F, Figure S7).

To aid physicians and researchers in predicting a patient’s long-term survival rate, CBioProfiler can generate a nomogram to predict the patient’s long-term survival rate. In this example, we included the patient’s risk score, estrogen receptor (ER) status, tumor size, lymph node metastasis status, age, and grade to draw a nomogram for predicting the OS probabilities of patients at 3-, 5-, and 10-year time points (Figure 4G). The users can estimate the survival probability of each patient based on the “Total point” which is the sum of the “Points” corresponding to each clinical feature. Then, we internally and externally validated and calibrated the performance of the nomogram (Figures S8 and S9).

### Case study: clinical annotation

**Figure 5:**
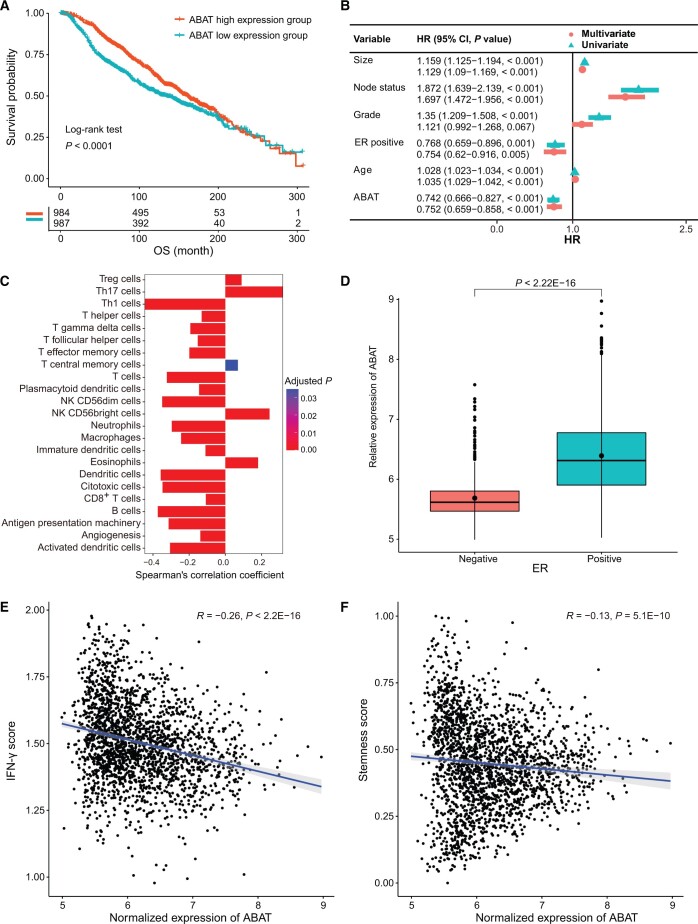
Clinical annotation of ABAT in the METABRIC cohort **A**. Survival differences between ABAT low and high expression groups. **B**. CoxPH model identifying the prediction ability of ABAT. **C**. Most correlated genes of ABAT based on Spearman’s correlation analysis. **D**. Relative expression of ABAT in the ER-negative and ER-positive groups. **E**. Correlation between the relative expression of ABAT and the IFN-γ score. **F**. Correlation between the relative expression of ABAT and the stemness score. ABAT, 4-aminobutyrate aminotransferase; METABRIC, the Molecular Taxonomy of Breast Cancer International Consortium; CI, confidence interval; HR, hazard ratio.

To further elucidate the clinical significance of the molecular markers screened by CBioProfiler, we chose 4-aminobutyrate aminotransferase (ABAT) for clinical annotation analysis. As shown in Table S4, the expression level of ABAT was closely related to the patient’s ER, tumor size, and grade. Survival analysis showed that the OS of breast cancer patients in the ABAT high expression group was significantly better than that in the ABAT low expression group (**Figure 5**A). After adjusting for other clinical factors, the expression level of ABAT still had independent prognostic significance for breast cancer patients (Figure 5B). Correlation analysis between the expression of ABAT and the enrichment score of single-sample GSEA (ssGSEA) analysis of the gene sets provided by Bindea et al. [43] showed that the expression of ABAT was significantly correlated with the enrichment of B cells, CD8^+^ T cells, cytotoxic cells, dendritic cells, eosinophils, immature dendritic cells, macrophages, neutrophils, natural killer (NK) CD56bright cells, NK CD56dim cells, plasmacytoid dendritic cells, T cells, T helper cells, T central memory cells, T effector memory cells, T follicular helper cells, T gamma delta cells, Th1 cells, Th17 cells, Treg cells, angiogenesis, and antigen presentation machinery (Figure 5C). Meanwhile, the expression of ABAT was negatively correlated several well-known immune checkpoint molecules (PDCD1, CD274, PDCD1LG2, CTLA4, PVR, LAG3, TIGIT, HAVCR2, VTCN1, CD86, CD28, CD80, CD27, CD40, IL2RB, TNFRSF9, TNFRSF4, ICOS, CD276, BTLA, KIR3DL1, CYBB, and SIGLEC7) (Figure S10) and stromal score, immune score, and estimate score (Figure S11) calculated using R package ESTIMATE. The expression of ABAT in the ER-positive group was significantly higher than that in the ER-negative group (Figure 5D). ABAT expression was also correlated with IFN-γ score (Figure 5E), stemness score (Figure 5F), and cytotoxic activity (Figure S12) of breast cancer. Finally, the expression of ABAT was significant correlated with many well-known cancer-related signaling pathways (Figure S13), hallmarks signatures (Figure S14), and metabolism pathways (Figure S15).

### Case study: biological annotation

**Figure 6:**
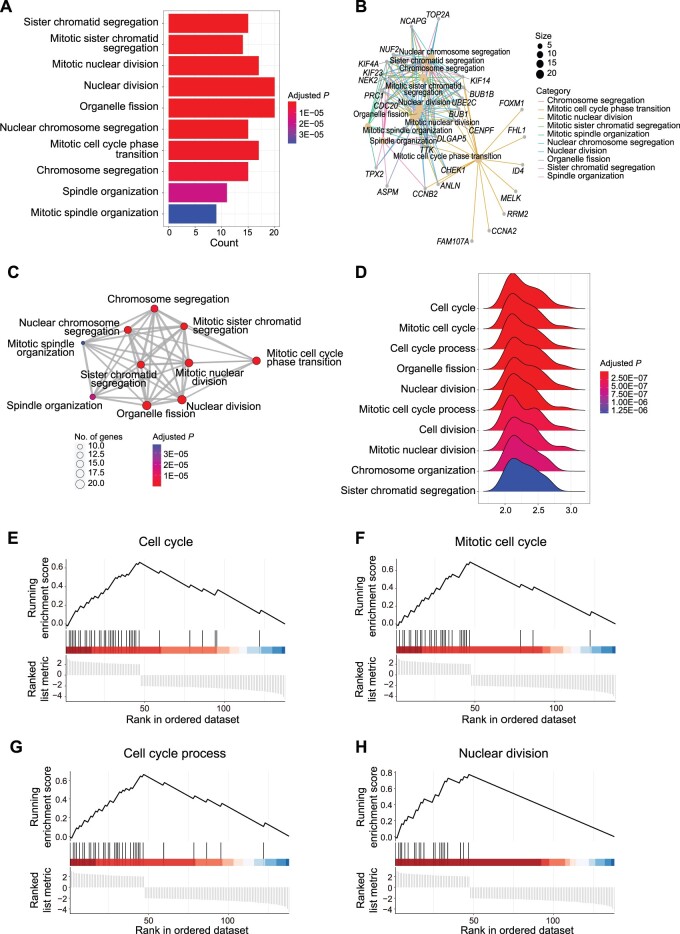
Functional enrichment analysis of the biomarkers identified by CBioProfiler Bar plot (**A**), gene-concept network (**B**), and enrichment map (**C**) showing the top 10 GO biological process terms where biomarkers were enriched. **D**.–**H**. Top GO biological process terms identified by GSEA.

CBioProfiler provides a variety of enrichment analysis and corresponding visualization methods, so that researchers can clarify the biological function and significance of the biomarkers that they screened. In this example, we performed GO enrichment analysis on 136 DEGs between the group A and group B at |log_2_ FC| > 2 in GSE31210 [41] by CBioProfiler. ORA and GSEA were implemented, respectively. **Figure 6**A–C showed the top 10 GO terms (sister chromatid segregation, mitotic sister chromatid segregation, mitotic nuclear division, nuclear division, organelle fission, nuclear chromosome segregation, mitotic cell cycle phase transition, chromosome segregation, spindle organization, and mitotic spindle organization) that the 136 DEGs were enriched in. While results of GSEA showed that these genes were mainly enriched in cell cycle, mitotic cell cycle, cell cycle process, and nuclear division (Figure 6D–H).

### Case study: cancer subtype identification, validation, and annotation

**Figure 7:**
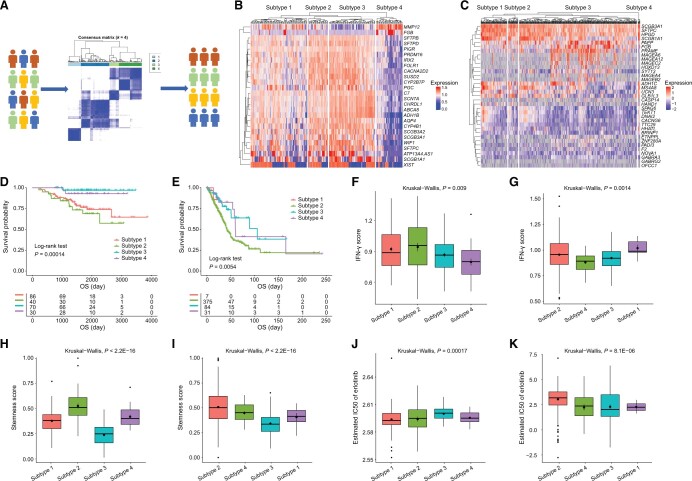
Identification, validation, and characterization of cancer subtypes **A**. Preprocessed gene expression profile of GSE31210 (the training set) was subjected to Monte Carlo simulation-based consensus clustering to identify cancer subtypes and validated on the TCGA-LUAD cohort (the validation set). **B**. DEGs among the different subtypes in the training set. **C**. DEGs among the different subtypes in the validation set. **D**. Survival differences of patients among the different subtypes in the training set. **E**. Survival differences of patients among the different subtypes in the validation set. **F**. Comparison of IFN-γ score among the different subtypes in the training set. **G**. Comparison of IFN-γ score among the different subtypes in the validation set. **H**. Comparison of stemness score among the different subtypes in the training set. **I**. Comparison of stemness score among the different subtypes in the validation set. **J**. Comparison of erlotinib response among the different subtypes in the training set. **K**. Comparison of erlotinib response among the different subtypes in the validation set. IC50, half maximal inhibitory concentration.

As mentioned above, GSE31210, published by Okayama et al., is a transcription profile of 226 patients with stage I and II lung adenocarcinomas [41]. Herein, we performed a Monte Carlo simulation-based consensus clustering analysis [36] of lung adenocarcinoma gene expression profiles that had undergone variable screening using the CoxPH model to identify potential subtypes of lung adenocarcinomas in the GSE31210 cohort (the training set) (**Figure 7**A, Figure S16; Table S5) and validated the subtypes on the TCGA-LUAD cohort (the validation set) [44] (Table S6).

In order to further clarify the potential clinical and biological significance of different subtypes, we conducted comparisons between different subtypes in the two cohorts from multiple aspects. Figure 7B and C showed the DEGs among different subtypes in the training set and the validation set, respectively. Survival analysis revealed significant differences among different subtypes (Figure 7D and E), and the corresponding time-dependent ROC analysis confirmed the robust predictive performance (Figure S17).

In addition, patients with different subtypes of lung adenocarcinomas had significant differences in clinical features (Tables S7 and S8), IFN-γ score (Figure 7F and G), stemness score (Figure 7H and I), erlotinib response (Figure 7J and K), cancer-related signaling pathways (Figures S18 and S19), ESTIMATE score (Figures S20 and S21), hallmark signature score (Figures S22 and S23), immune microenvironment (Figures S24 and S25), immune checkpoints (Figures S26 and S27), and metabolic related signaling pathways (Figures S28 and S29).

### Case study: meta-analysis

To assist users in synthesizing and drawing conclusions from a larger body of evidence, CBioProfiler enables meta-analysis on the predictive ability of a biomarker using multiple gene expression and prognosis studies. This facilitates the generation of more robust and reliable results compared to individual studies alone. As mentioned above, the prognostication ability of ABAT in breast cancer has been analyzed in the breast cancer gene expression study METABRIC. To draw a more robust and generalized conclusion, we included a total of 31 breast cancer gene expression studies (GSE3143, GSE10886_GPL1390, GSE10886_GPL887, GSE18229_GPL1390, GSE18229_GPL887, GSE22226_GPL1708, GSE22226_GPL4133, GSE2607_GPL1390, GSE2607_GPL887, GSE6130_GPL1390, GSE6130_GPL887, Caldas_2007, GSE12071, GSE10510, GSE159956, GSE22133_GPL5345, E_TABM_158, GSE1456_GPL96, GSE16446, GSE20711, GSE37751, GSE42568, GSE48390, GSE58812, GSE7390, ICGC_BRCA_FR, ICGC_BRCA_KR, TCGA_BRCA, METABRIC, Veer_2002, and Korkola_2007) containing 6,992 patients with breast cancer. As shown in Figure S30, the result of meta-analysis confirmed that ABAT was significantly associated with the OS of patients with breast cancer (HR = 0.83, 95% CI: 0.75–0.9, *P* < 0.01).

## Discussion

CBioProfiler, to our knowledge, is the first web and standalone application that integrates multiple popular machine learning algorithms and CV strategies for cancer biomarker and subtype identification, validation, and clinical and biological annotation. Moreover, we also developed CuratedCancerPrognosisData, an R package that reviewed, integrated 47,686 clinical samples from 268 gene expression studies of 43 common blood and solid tumors. Compared with other similar online tools or standalone apps (Table 1) based on public data from TCGA, GEO, *etc*., CBioProfiler boasts the following advantages. (1) CBioProfiler encompasses the widest range of disease types and samples, offers fully accessible the curated data for academic use, and provides a personalized data submission interface for researchers to analyze their own data. (2) CBioProfiler offers the most comprehensive set of analysis modules. These modules can be used either as a complete pipeline for screening, evaluating, validating, and annotating tumor molecular markers or individually to achieve specific research goals. For example, users can perform DEG, WGCNA, and SRG analyses separately, or they can perform clinical annotations, such as survival analysis and immune infiltration analysis, for some molecular markers that they are interested in. (3) CBioProfiler is available in both an online version and a standalone local app version. The online version allows users to promptly conduct research on related molecular markers, while the standalone app version can be downloaded for more computationally intensive analysis.

We developed and introduced CBioProfiler, a web and standalone pipeline that reviewed, curated, and integrated the gene expression data and corresponding clinical information of 47,686 clinical samples from 268 gene expression studies of 43 common blood and solid tumors. This was done in order to identify, validate, and annotate cancer biomarkers and subtypes from the molecular level to clinical settings.

## Code availability

The source code of CBioProfiler can be downloaded from https://github.com/liuxiaoping2020/CBioProfiler or https://gitee.com/liuxiaoping2020/CBioProfiler. The source code for CuratedCancerPrognosisData has been deposited at https://zenodo.org/records/7481234.

## Supplementary Material

qzae045_Supplementary_Data

## Data Availability

CBioProfiler is publicly available as a web server at https://www.cbioprofiler.com/ and https://cbioprofiler.znhospital.cn/CBioProfiler/.

## CRediT author statement

**Xiaoping Liu:** Conceptualization, Supervision, Methodology, Validation, Software, Formal analysis, Investigation, Data curation, Writing – original draft, Writing – review & editing. **Xinghuan Wang:** Conceptualization, Supervision, Resources, Writing – review & editing. **Hongjie Shi:** Methodology, Validation. **Sheng Li:** Conceptualization, Writing – review & editing. **Zisong Wang:** Methodology, Validation, Writing – review & editing. All authors have read and approved the final manuscript.
