# Supplementary material for: CBioProfiler: A Web and Standalone Pipeline for Cancer Biomarker and Subtype Characterization
Source: Genomics Proteomics Bioinformatics. 2024 Jun 12;22(3):qzae045. doi: 10.1093/gpbjnl/qzae045 (PMC11464420; doi:10.1093/gpbjnl/qzae045)
Supplement: qzae045_Supplementary_Data [file qzae045_supplementary_data.zip › Supplementary material captions.docx]

**Supplementary material**

**Figure S1 Flow chart of curation of public gene expression studies integrated in CuratedCancerPrognosisData**

CGGA, Chinese Glioma Genome Atlas; PFS, progression-free survival; RFS, relapse-free survival; DFS, disease-free survival; DMFS, distant metastasis-free survival; RMA, robust multichip average; TPM, transcripts per kilobase million; FPKM, fragments per kilobase million ; RSEM, RNA-Seq by expectation-maximization.

**Figure S2 Workflow of cross-validation**

**Figure S3 Workflow of nested cross-validation**

**Figure S4 Detection of outliers based on sample dendrogram**

**Figure S5 Comparison of C-indexes of three survival learners based on cross-validation and bootstraps**

**Figure S6 Cox proportion hazards regression model identifying independent prognostication role of the risk score in the training set (A) and test set (B)**

**Figure S7 Cox proportion hazards regression model identifying independent prognostication role of the risk score in the validation set**

PGR, progesterone receptor; HER2, human epidermal growth factor receptor 2.

**Figure S8 Internally (A) and externally (B) validation of the nomogram**

**Figure S9 Internally and externally calibration of the nomogram in the training set (A**–**C), test set (D**–**F), and validation set (G**–**I)**

**Figure S10 The correlation between the expression of ABAT and well-known immune checkpoint molecules**

The correlation analysis was performed using Spearman’s rank correlation.

**Figure S11 The correlation between the expression of ABAT and (A) stromal score, (B) immune score, and (C) estimate score calculated using R packages ESTIMATE**

The correlation analysis was performed using Spearman’s rank correlation.

**Figure S12 The correlation between the expression of ABAT and** **cytotoxic activity**

The correlation analysis was performed using Spearman’s rank correlation.

**Figure S13 The correlation between the expression of ABAT and well-known** **cancer related pathway**

The correlation analysis was performed using Spearman’s rank correlation.

**Figure S14 The correlation between the expression of ABAT and** **hallmarks**

**signature**

The correlation analysis was performed using Spearman’s rank correlation.

**Figure S15 The correlation between the expression of ABAT and** **metabolism pathway**

The correlation analysis was performed using Spearman’s rank correlation.

**Figure S16 Useful indexes for the determination of best K of clusters**

**A**. CDF. **B**. Entropy. **C**. *P* values from the beta distribution. **D**. RCSI. CDF, cumulative distribution function; RCSI, relative cluster stability index.

**Figure S17 Prediction performance of the cancer subtypes in the training set (A) and validation set (B)**

**Figure S18 Comparison of common cancer related pathway score among the cancer subtypes in the training set**

**Figure S19 Comparison of common cancer related pathway score among the cancer subtypes in the validation set**

**Figure S20 Comparison of the estimation of stromal and immune cells (ESTIMATE score) among the cancer subtypes in the training set**

**Figure S21 Comparison of the estimation of stromal and immune cells (ESTIMATE score) among the cancer subtypes in the validation set**

**Figure S22 Comparison of hallmark signature score among the cancer subtypes in the training set**

**Figure S23 Comparison of hallmark signature score among the cancer subtypes in the validation set**

**Figure S24 Comparison of immune cell infiltrations among the cancer subtypes in the training set**

**Figure S25 Comparison of immune cell infiltrations among the cancer subtypes in the validation set**

**Figure S26 Comparison of the expression of immune checkpoint molecules among the cancer subtypes in the training set**

**Figure S27 Comparison of the expression of immune checkpoint molecules among the cancer subtypes in the validation set**

**Figure S28 Comparison of the metabolic related signaling pathways among the cancer subtypes in the training set**

**Figure S29 Comparison of the metabolic related signaling pathways among the cancer subtypes in the validation set**

**Figure S30 Meta-analysis of the prognosis role of ABAT in breast cancer**

SE, .

**Table S1 Parameter setting range of each model used in this model**

**Table S2 Public gene expression studies that included in the present study**

**Table S3 Univariate Cox proportional hazards regression model on the overall survival of patients with breast cancer in METABRIC cohort**

**Table S4 Differences between ABAT high expression group and ABAT low expression group**

**Table S5 Subtype assignment in the training cohort**

**Table S6 Subtype assignment in the validation cohort TCGA-LUAD**

**Table S7 Comparison of clinical features in the GSE31210 cohort (training set)**

**Table S8 Comparison of clinical features in the TCGA-LUAD cohort (validation set)**
