# Supplementary material for: CBioProfiler: A Web and Standalone Pipeline for Cancer Biomarker and Subtype Characterization
Source: Genomics Proteomics Bioinformatics. 2024 Jun 12;22(3):qzae045. doi: 10.1093/gpbjnl/qzae045 (PMC11464420; doi:10.1093/gpbjnl/qzae045)
Supplement: qzae045_Supplementary_Data [file qzae045_supplementary_data.zip › Table S1-done.docx]

**Table S1 Parameter setting range of each model used in this model**

| **Learner** | **Parameter** | **Parameter type** | **Parameter range** |
| --- | --- | --- | --- |
| Random forest | mtry | Integer | 1, 15 |
|  | node size | Discrete | 3, 5, 8, 10, 15, 18, 20 |
|  | ntree | Discrete | 500, 1000, 1500, 2000 |
|  | node depth | Integer | 5, 20 |
| GLMBoost | mstop | Integer | 1E2, 1E3 |
|  | nu | Discrete | 0.05, 0.1, 0.3, 0.5, 0.8, 1 |
| CoxBoost | stepno | Integer | Lower = 50, upper = 200 |
| Elastic net | alpha | Numeric | Lower = 0, upper = 1 |
|  | s | Numeric | Lower = 0.001, upper = 30 |
| Ridge | s | Numeric | Lower = 0, upper = 20 |
| LASSO | s | Numeric | Lower = 0, upper = 20 |

*Note*: LASSO, least absolute shrinkage and selection operator.
