# Supplementary material for: CBioProfiler: A Web and Standalone Pipeline for Cancer Biomarker and Subtype Characterization
Source: Genomics Proteomics Bioinformatics. 2024 Jun 12;22(3):qzae045. doi: 10.1093/gpbjnl/qzae045 (PMC11464420; doi:10.1093/gpbjnl/qzae045)
Supplement: qzae045_Supplementary_Data [file qzae045_supplementary_data.zip › Table S2-done.docx]

**Table S2 Public gene expression studies that included in the present study**

| **ID** | **Cancer** | **Dataset** | **Total genes** | **Total samples** | **Platform** | **Last update** | **Link** | **PMID** |
| --- | --- | --- | --- | --- | --- | --- | --- | --- |
| 1 | Acute lymphoblastic leukemia | TARGET_ALL_P3 | 58,387 | 117 | Illumina HiSeq | 2019 | https://xenabrowser.net/datapages/ | 25207766 |
| 2 | Acute lymphoblastic leukemia | E_MTAB_1216 | 12,547 | 101 | Affymetrix GeneChip Human Genome HG-U133A | 2014 | https://www.ebi.ac.uk/arrayexpress/experiments/E-MTAB-1216 | NA |
| 3 | Acute lymphoblastic leukemia | E_MTAB_1205 | 21,653 | 38 | Affymetrix GeneChip Human Genome U133 Plus 2.0 | 2014 | https://www.ebi.ac.uk/arrayexpress/experiments/E-MTAB-1205 | 23436797 |
| 4 | Acute myeloid leukemia | TARGET_AML | 58,387 | 145 | Illumina HiSeq | 2019 | https://xenabrowser.net/datapages/ | 26941285 |
| 5 | Acute myeloid leukemia | GSE12417_GPL570 | 21,653 | 79 | Affymetrix Human Genome U133 Plus 2.0 Array | 2021 | https://www.ncbi.nlm.nih.gov/geo/query/acc.cgi?acc=GSE12417 | 18716133 |
| 6 | Acute myeloid leukemia | GSE12417_GPL96 | 12,547 | 163 | Affymetrix Human Genome U133A Array | 2021 | https://www.ncbi.nlm.nih.gov/geo/query/acc.cgi?acc=GSE12417 | 18716133 |
| 7 | Acute myeloid leukemia | GSE12417_GPL97 | 10,603 | 163 | Affymetrix Human Genome U133B Array | 2021 | https://www.ncbi.nlm.nih.gov/geo/query/acc.cgi?acc=GSE12417 | 18716133 |
| 8 | Acute myeloid leukemia | TCGA_LAML | 34,849 | 117 | HTSeq | 2021 | https://portal.gdc.cancer.gov/projectsTCGA-LAML | 23634996 |
| 9 | Adrenocortical carcinoma | TCGA_ACC | 34,849 | 79 | HTSeq | 2021 | https://portal.gdc.cancer.gov/projectsTCGA-ACC | 27165744 |
| 10 | Adult soft tissue sarcomas | TCGA_SARC | 34,849 | 255 | HTSeq | 2021 | https://portal.gdc.cancer.gov/projectsTCGA-SARC | 29100075 |
| 11 | Anaplastic large cell lymphomas | E_TABM_117 | 12,547 | 37 | Affymetrix GeneChip Human Genome HG-U133A | 2014 | https://www.ebi.ac.uk/arrayexpress/experiments/E-TABM-117 | 17077326 |
| 12 | Bladder cancer | GSE13507 | 24,357 | 256 | Illumina human-6 v2.0 expression beadchip | 2020 | https://www.ncbi.nlm.nih.gov/geo/query/acc.cgi?acc=GSE13507 | 20059769 |
| 13 | Bladder cancer | GSE5287 | 12,178 | 30 | Affymetrix Human Genome U133A Array | 2018 | https://www.ncbi.nlm.nih.gov/geo/query/acc.cgi?acc=GSE5287 | 17671123 |
| 14 | Bladder cancer | GSE1827 | 6225 | 80 | JAKE | 2015 | https://www.ncbi.nlm.nih.gov/geo/query/acc.cgi?acc=GSE1827 | 15930339 |
| 15 | Bladder cancer | GSE19915_GPL3883 | 10,314 | 84 | Swegene Human 27K RAP UniGene188 array | 2012 | https://www.ncbi.nlm.nih.gov/geo/query/acc.cgi?acc=GSE19915 | 20406976 |
| 16 | Bladder cancer | GSE19915_GPL5186 | 11,671 | 98 | SWEGENE H_v3.0.1 35K | 2012 | https://www.ncbi.nlm.nih.gov/geo/query/acc.cgi?acc=GSE19915 | 20406976 |
| 17 | Bladder cancer | GSE32894 | 16,940 | 308 | Illumina HumanHT-12 V3.0 expression beadchip | 2020 | https://www.ncbi.nlm.nih.gov/geo/query/acc.cgi?acc=GSE32894 | 22553347 |
| 18 | Bladder cancer | E_MTAB_4321 | 38,267 | 476 | FPKM normalized RNAseq data | NA | https://www.ebi.ac.uk/arrayexpress/experiments/E-MTAB-4321 | 27321955 |
| 19 | Bladder cancer | GSE19423 | 24,357 | 48 | Illumina human-6 v2.0 expression beadchip | 2013 | https://www.ncbi.nlm.nih.gov/geo/query/acc.cgi?acc=GSE19423 | 20233890 |
| 20 | Bladder cancer | GSE48276 | 20,818 | 116 | Illumina HumanHT-12 WG-DASL V4.0 R2 expression beadchip | 2017 | https://www.ncbi.nlm.nih.gov/geo/query/acc.cgi?acc=GSE48276 | 24525232 |
| 21 | Bladder cancer | GSE19750 | 21,653 | 48 | Affymetrix Human Genome U133 Plus 2.0 Array | 2019 | https://www.ncbi.nlm.nih.gov/geo/query/acc.cgi?acc=GSE19750 | 24238056 |
| 22 | Bladder cancer | GSE31684 | 21,653 | 93 | Affymetrix Human Genome U133 Plus 2.0 Array | 2019 | https://www.ncbi.nlm.nih.gov/geo/query/acc.cgi?acc=GSE31684 | 22228636 |
| 23 | Bladder cancer | TCGA_BLCA | 34,849 | 400 | HTSeq | 2021 | https://portal.gdc.cancer.gov/projectsTCGA-BLCA | 24476821 |
| 24 | Bone cancer-Ewing sarcoma | ICGC_BOCA_FR | 24,818 | 57 | HTSeq | 2019 | https://dcc.icgc.org/releases/current/Projects/BOCA-FR | 25223734 |
| 25 | Brain tumor-GBM | E_MTAB_951 | 21,653 | 23 | Affymetrix GeneChip Human Genome U133 Plus 2.0 | 2014 | https://www.ebi.ac.uk/arrayexpress/experiments/E-MTAB-951 | 24804210 |
| 26 | Brain tumor-GBM | GSE13041_GPL570 | 21,653 | 27 | Affymetrix Human Genome U133 Plus 2.0 Array | 2019 | https://www.ncbi.nlm.nih.gov/geo/query/acc.cgi?acc=GSE13041 | 18940004 |
| 27 | Brain tumor-GBM | GSE13041_GPL8300 | 8619 | 49 | Affymetrix Human Genome U95 Version 2 Array | 2019 | https://www.ncbi.nlm.nih.gov/geo/query/acc.cgi?acc=GSE13041 | 18940004 |
| 28 | Brain tumor-GBM | GSE13041_GPL96 | 12,547 | 191 | Affymetrix Human Genome U133A Array | 2019 | https://www.ncbi.nlm.nih.gov/geo/query/acc.cgi?acc=GSE13041 | 18940004 |
| 29 | Brain tumor-GBM | GSE42669 | 20,202 | 58 | Affymetrix Human Gene 1.0 ST Array | 2018 | https://www.ncbi.nlm.nih.gov/geo/query/acc.cgi?acc=GSE42669 | 23333277 |
| 30 | Brain tumor-GBM | GSE7696 | 21,653 | 84 | Affymetrix Human Genome U133 Plus 2.0 Array | 2019 | https://www.ncbi.nlm.nih.gov/geo/query/acc.cgi?acc=GSE7696 | 18565887 |
| 31 | Brain tumor-GBM | TCGA_GBM | 34,849 | 143 | HTSeq | 2021 | https://portal.gdc.cancer.gov/projectsTCGA-GBM | 18772890 |
| 32 | Brain tumor-Glioma | E_MTAB_2768 | 21,653 | 71 | Affymetrix GeneChip Human Genome U133 Plus 2.1 | 2018 | https://www.ebi.ac.uk/arrayexpress/experiments/E-MTAB-2768 | 26068201 |
| 33 | Brain tumor-Glioma | GSE2817 | 21,653 | 25 | Affymetrix Human Genome U133 Plus 2.0 Array | 2019 | https://www.ncbi.nlm.nih.gov/geo/query/acc.cgi?acc=GSE2817 | 17140431 |
| 34 | Brain tumor-Glioma | TCGA_LGG | 34,849 | 495 | HTSeq | 2021 | https://portal.gdc.cancer.gov/projectsTCGA-LGG | 26061751 |
| 35 | Brain tumor-Glioma/GBM | GSE108474 | 23,519 | 541 | Affymetrix Human Genome U133 Plus 2.0 Array | 2019 | https://www.ncbi.nlm.nih.gov/geo/query/acc.cgi?acc=GSE108474 | 30106394 |
| 36 | Brain tumor-Glioma/GBM | CGGA_301 | 19,416 | 300 | Agilent Whole Human Genome (Array) | 2021 | http://www.cgga.org.cn/download.jsp | 27564467 |
| 37 | Brain tumor-Glioma/GBM | E_MTAB_3892 | 21,653 | 175 | Affymetrix GeneChip Human Genome U133 Plus 2.0 | 2018 | https://www.ebi.ac.uk/arrayexpress/experiments/E-MTAB-3892 | 27090007 |
| 38 | Brain tumor-Glioma/GBM | GSE16011 | 21,653 | 284 | Affymetrix GeneChip Human Genome U133 Plus 2.0 Array | 2014 | https://www.ncbi.nlm.nih.gov/geo/query/acc.cgi?acc=GSE16011 | 19920198 |
| 39 | Brain tumor-Glioma/GBM | GSE4271_GPL96 | 12,547 | 100 | Affymetrix Human Genome U133B Array | 2019 | https://www.ncbi.nlm.nih.gov/geo/query/acc.cgi?acc=GSE4271 | 16530701 |
| 40 | Brain tumor-Glioma/GBM | GSE4271_GPL97 | 10,603 | 100 | Affymetrix Human Genome U133B Array | 2019 | https://www.ncbi.nlm.nih.gov/geo/query/acc.cgi?acc=GSE4271 | 16530701 |
| 41 | Brain tumor-Glioma/GBM | GSE4412_GPL96 | 12,547 | 85 | Affymetrix Human Genome U134A Array | 2018 | https://www.ncbi.nlm.nih.gov/geo/query/acc.cgi?acc=GSE4412 | 15374961 |
| 42 | Brain tumor-Glioma/GBM | GSE4412_GPL97 | 10,603 | 85 | Affymetrix Human Genome U134B Array | 2018 | https://www.ncbi.nlm.nih.gov/geo/query/acc.cgi?acc=GSE4412 | 15374961 |
| 43 | Brain tumor-Glioma/GBM | CGGA_325 | 24,326 | 325 | Illumina HiSeq 2000 or 2500 | 2021 | http://www.cgga.org.cn/download.jsp | 28291232 |
| 44 | Brain tumor-Glioma/GBM | CGGA_693 | 23,987 | 693 | Illumina HiSeq | 2021 | http://www.cgga.org.cn/download.jsp | 25031032 |
| 45 | Brain tumor-Medulloblastoma | GSE30074 | 20,202 | 30 | Affymetrix Human Gene 1.0 ST Array | 2019 | https://www.ncbi.nlm.nih.gov/geo/query/acc.cgi?acc=GSE30074 | 22090452 |
| 46 | Brain tumor-Medulloblastoma | GSE37418 | 21,653 | 76 | Affymetrix Human Genome U133 Plus 2.0 Array | 2019 | https://www.ncbi.nlm.nih.gov/geo/query/acc.cgi?acc=GSE37418 | 22722829 |
| 47 | Brain tumor-Meningioma | GSE16581 | 21,653 | 68 | Affymetrix GeneChip Human Genome U133 Plus 2.1 Array | 2019 | https://www.ncbi.nlm.nih.gov/geo/query/acc.cgi?acc=GSE16581 | 20015288 |
| 48 | Breast cancer | E_MTAB_7201 | 34,729 | 94 | Agilent-072363 SurePrint G3 Human GE v3 8x60K Microarray 039494 | 2018 | https://www.ebi.ac.uk/arrayexpress/experiments/E-MTAB-7201 | 28123884 |
| 49 | Breast cancer | GSE3143 | 8619 | 158 | Affymetrix HGU95 | 2018 | https://www.ncbi.nlm.nih.gov/geo/query/acc.cgi?acc=GSE3143 | 16273092 |
| 50 | Breast cancer | GSE10886_GPL1390 | 10,040 | 197 | Agilent Human 1A Oligo UNC custom Microarrays | 2017 | https://www.ncbi.nlm.nih.gov/geo/query/acc.cgi?acc=GSE10886 | 19204204 |
| 51 | Breast cancer | GSE10886_GPL887 | 16,546 | 27 | Agilent-012097 Human 1A Microarray (V2) G4110B | 2017 | https://www.ncbi.nlm.nih.gov/geo/query/acc.cgi?acc=GSE10886 | 19204204 |
| 52 | Breast cancer | GSE18229_GPL1390 | 10,040 | 199 | Agilent Human 1A Oligo UNC custom Microarrays | 2017 | https://www.ncbi.nlm.nih.gov/geo/query/acc.cgi?acc=GSE18229 | 20813035 |
| 53 | Breast cancer | GSE18229_GPL887 | 16,546 | 94 | Agilent-012097 Human 1A Microarray (V2) G4110B (Feature Number version) | 2017 | https://www.ncbi.nlm.nih.gov/geo/query/acc.cgi?acc=GSE18229 | 20813035 |
| 54 | Breast cancer | GSE22226_GPL1708 | 18,841 | 129 | Agilent-012391 Whole Human Genome Oligo Microarray G4112A (Feature Number version) | 2018 | https://www.ncbi.nlm.nih.gov/geo/query/acc.cgi?acc=GSE22226 | 22198468 |
| 55 | Breast cancer | GSE22226_GPL4133 | 19,749 | 20 | Agilent-014850 Whole Human Genome Microarray 4x44K G4112F (Feature Number version) | 2018 | https://www.ncbi.nlm.nih.gov/geo/query/acc.cgi?acc=GSE22226 | 22198468 |
| 56 | Breast cancer | GSE2607_GPL1390 | 10,040 | 64 | Agilent Human 1A Oligo UNC custom Microarrays | 2017 | https://www.ncbi.nlm.nih.gov/geo/query/acc.cgi?acc=GSE2607 | 16626501 |
| 57 | Breast cancer | GSE2607_GPL887 | 16,546 | 50 | Agilent-012097 Human 1A Microarray (V2) G4110B (Feature Number version) | 2017 | https://www.ncbi.nlm.nih.gov/geo/query/acc.cgi?acc=GSE2607 | 16626501 |
| 58 | Breast cancer | GSE6130_GPL1390 | 10,040 | 96 | Agilent Human 1A Oligo UNC custom Microarrays | 2017 | https://www.ncbi.nlm.nih.gov/geo/query/acc.cgi?acc=GSE6130 | 17525107 |
| 59 | Breast cancer | GSE6130_GPL887 | 16,546 | 53 | Agilent-012097 Human 1A Microarray (V2) G4110B (Feature Number version) | 2017 | https://www.ncbi.nlm.nih.gov/geo/query/acc.cgi?acc=GSE6130 | 17525107 |
| 60 | Breast cancer | GSE19536_GPL6480 | 19,595 | 114 | Agilent-014850 Whole Human Genome Microarray 4x44K G4112F (Probe Name version) | 2020 | https://www.ncbi.nlm.nih.gov/geo/query/acc.cgi?acc=GSE19536 | 21364938 |
| 61 | Breast cancer | Caldas_2007 | 14,708 | 135 | Agilent Human 1A 60-mer Oligo Microarray | 2011 | https://pubmed.ncbi.nlm.nih.gov/16936776/ | 16936776 |
| 62 | Breast cancer | GSE16987 | 18,196 | 161 | Illumina humanRef-8 v2.0 expression beadchip | 2015 | https://www.ncbi.nlm.nih.gov/geo/query/acc.cgi?acc=GSE16987 | 21939527 |
| 63 | Breast cancer | GSE12071 | 12,697 | 46 | SWEGENE H_v3.0.1 35K | 2012 | https://www.ncbi.nlm.nih.gov/geo/query/acc.cgi?acc=GSE12071 | 18778486 |
| 64 | Breast cancer | GSE9893 | 14,819 | 155 | MLRG Human 21K V12.0 | 2015 | https://www.ncbi.nlm.nih.gov/geo/query/acc.cgi?acc=GSE9893 | 18347175 |
| 65 | Breast cancer | GSE6577 | 6742 | 88 | Swegene Human 27K RAP UniGene188 array | 2019 | https://www.ncbi.nlm.nih.gov/geo/query/acc.cgi?acc=GSE6577 | 17404078 |
| 66 | Breast cancer | GSE10510 | 19,852 | 152 | DKFZ Division of Molecular Genome Analysis Human Operon 4.0 oligo Array 35k | 2013 | https://www.ncbi.nlm.nih.gov/geo/query/acc.cgi?acc=GSE10510 | 18592372 |
| 67 | Breast cancer | GSE37181 | 25,438 | 123 | Illumina HumanWG-6 v3.0 expression beadchip | 2019 | https://www.ncbi.nlm.nih.gov/geo/query/acc.cgi?acc=GSE37181 | 24853384 |
| 68 | Breast cancer | GSE159956 | 12,201 | 295 | Rosetta (Merck) GEL Breast Tumor Profiles | 2021 | https://www.ncbi.nlm.nih.gov/geo/query/acc.cgi?acc=GSE159956 | NA |
| 69 | Breast cancer | GSE22133_GPL5345 | 8427 | 359 | SWEGENE H_v2.1.1 55K | 2012 | https://www.ncbi.nlm.nih.gov/geo/query/acc.cgi?acc=GSE22133 | 20576095 |
| 70 | Breast cancer | GSE22219 | 21,438 | 216 | Illumina humanRef-8 v1.0 expression beadchip | 2013 | https://www.ncbi.nlm.nih.gov/geo/query/acc.cgi?acc=GSE22219 | 21737487 |
| 71 | Breast cancer | GSE1379 | 11,728 | 60 | Arcturus 22k human oligonucleotide microarray | 2012 | https://www.ncbi.nlm.nih.gov/geo/query/acc.cgi?acc=GSE1379 | 15193263 |
| 72 | Breast cancer | GSE45725 | 18,630 | 340 | Illumina HumanRef-8 v3.0 expression beadchip | 2017 | https://www.ncbi.nlm.nih.gov/geo/query/acc.cgi?acc=GSE45725 | 24996446 |
| 73 | Breast cancer | GSE175692 | 771 | 184 | nCounter Breast Cancer 360 Panel | 2021 | https://www.ncbi.nlm.nih.gov/geo/query/acc.cgi?acc=GSE175692 | 34051058 |
| 74 | Breast cancer | E_TABM_158 | 12,888 | 100 | Affymetrix High Throughput Array U133AA of Av2 | 2014 | https://www.ebi.ac.uk/arrayexpress/experiments/E-TABM-158 | 17157792 |
| 75 | Breast cancer | GSE11121 | 12,547 | 200 | Affymetrix Human Genome U133A Array | 2020 | https://www.ncbi.nlm.nih.gov/geo/query/acc.cgi?acc=GSE11121 | 18593943 |
| 76 | Breast cancer | GSE12093 | 12,547 | 136 | Affymetrix Human Genome U133A Array | 2018 | https://www.ncbi.nlm.nih.gov/geo/query/acc.cgi?acc=GSE12093 | 18821012 |
| 77 | Breast cancer | GSE12276 | 21,653 | 204 | Affymetrix HGU | 2019 | https://www.ncbi.nlm.nih.gov/geo/query/acc.cgi?acc=GSE12276 | 19421193 |
| 78 | Breast cancer | GSE1456_GPL96 | 12,547 | 159 | Affymetrix HGU | 2018 | https://www.ncbi.nlm.nih.gov/geo/query/acc.cgi?acc=GSE1456 | 16280042 |
| 79 | Breast cancer | GSE146558 | 21,653 | 109 | Affymetrix Human Genome U133 Plus 2.0 Array | 2021 | https://www.ncbi.nlm.nih.gov/geo/query/acc.cgi?acc=GSE146558 | 34387660 |
| 80 | Breast cancer | GSE158309 | 12,547 | 461 | Affymetrix Human Genome U133A Array | 2020 | https://www.ncbi.nlm.nih.gov/geo/query/acc.cgi?acc=GSE158309 | 33003293 |
| 81 | Breast cancer | GSE16446 | 21,653 | 120 | Affymetrix Human Genome U133 Plus 2.0 Array | 2019 | https://www.ncbi.nlm.nih.gov/geo/query/acc.cgi?acc=GSE16446 | 21422418 |
| 82 | Breast cancer | GSE17705 | 12,547 | 298 | Affymetrix Human Genome U133A Array | 2018 | https://www.ncbi.nlm.nih.gov/geo/query/acc.cgi?acc=GSE17705 | 20697068 |
| 83 | Breast cancer | GSE19615 | 21,653 | 115 | Affymetrix Human Genome U133 Plus 2.0 Array | 2019 | https://www.ncbi.nlm.nih.gov/geo/query/acc.cgi?acc=GSE19615 | 20098429 |
| 84 | Breast cancer | GSE2034 | 12,547 | 286 | Affymetrix Human Genome U134A Array | 2018 | https://www.ncbi.nlm.nih.gov/geo/query/acc.cgi?acc=GSE2034 | 17420468, 15721472 |
| 85 | Breast cancer | GSE20711 | 21,653 | 90 | Affymetrix Human Genome U133 Plus 2.0 Array | 2021 | https://www.ncbi.nlm.nih.gov/geo/query/acc.cgi?acc=GSE20711 | 21910250 |
| 86 | Breast cancer | GSE21653 | 21,653 | 266 | Affymetrix Human Genome U133 Plus 2.0 Array | 2019 | https://www.ncbi.nlm.nih.gov/geo/query/acc.cgi?acc=GSE21653 | 20490655 |
| 87 | Breast cancer | GSE25055 | 12,547 | 310 | Affymetrix Human Genome U136A Array | 2018 | https://www.ncbi.nlm.nih.gov/geo/query/acc.cgi?acc=GSE25055 | 21558518 |
| 88 | Breast cancer | GSE25065 | 12,547 | 198 | Affymetrix Human Genome U137A Array | 2018 | https://www.ncbi.nlm.nih.gov/geo/query/acc.cgi?acc=GSE25065 | 21558518 |
| 89 | Breast cancer | GSE2603 | 12,547 | 121 | Affymetrix Human Genome U133A Array | 2020 | https://www.ncbi.nlm.nih.gov/geo/query/acc.cgi?acc=GSE2603 | 16049480 |
| 90 | Breast cancer | GSE2990 | 12,547 | 189 | Affymetrix Human Genome U133A Array | 2018 | https://www.ncbi.nlm.nih.gov/geo/query/acc.cgi?acc=GSE2990 | 16478745, 17401012 |
| 91 | Breast cancer | GSE3494_GPL96 | 12,547 | 251 | Affymetrix Human Genome U133A Array | 2019 | https://www.ncbi.nlm.nih.gov/geo/query/acc.cgi?acc=GSE3494 | 16141321 |
| 92 | Breast cancer | GSE37751 | 20,202 | 108 | Affymetrix Human Gene 1.0 ST Array | 2019 | https://www.ncbi.nlm.nih.gov/geo/query/acc.cgi?acc=GSE37751 | 24316975 |
| 93 | Breast cancer | GSE42568 | 21,653 | 121 | Affymetrix Human Genome U133 Plus 2.0 Array | 2019 | https://www.ncbi.nlm.nih.gov/geo/query/acc.cgi?acc=GSE42568 | 23740839 |
| 94 | Breast cancer | GSE45255 | 12,547 | 139 | Affymetrix Human Genome U133A Array | 2018 | https://www.ncbi.nlm.nih.gov/geo/query/acc.cgi?acc=GSE45255 | 23618380 |
| 95 | Breast cancer | GSE48390 | 21,653 | 81 | Affymetrix Human Genome U133 Plus 2.0 Array | 2019 | https://www.ncbi.nlm.nih.gov/geo/query/acc.cgi?acc=GSE48390 | 24098497 |
| 96 | Breast cancer | GSE4922_GPL96 | 12,547 | 249 | Affymetrix Human Genome U133A Array | 2018 | https://www.ncbi.nlm.nih.gov/geo/query/acc.cgi?acc=GSE4922 | 17079448 |
| 97 | Breast cancer | GSE53031 | 19,040 | 167 | Affymetrix Human Genome U219 Array | 2019 | https://www.ncbi.nlm.nih.gov/geo/query/acc.cgi?acc=GSE53031 | 24825746 |
| 98 | Breast cancer | GSE5327 | 12,547 | 58 | Affymetrix Human Genome U134A Array | 2018 | https://www.ncbi.nlm.nih.gov/geo/query/acc.cgi?acc=GSE5327 | 17420468 |
| 99 | Breast cancer | GSE58644 | 20,202 | 321 | Affymetrix Gene1.0ST | 2018 | https://www.ncbi.nlm.nih.gov/geo/query/acc.cgi?acc=GSE58644 | 25284793 |
| 100 | Breast cancer | GSE58812 | 21,653 | 107 | Affymetrix Human Genome U133 Plus 2.0 Array | 2019 | https://www.ncbi.nlm.nih.gov/geo/query/acc.cgi?acc=GSE58812 | 25887482 |
| 101 | Breast cancer | GSE6532_GPL570 | 21,653 | 87 | Affymetrix Human Genome U133 Plus 2.0 Array | 2019 | https://www.ncbi.nlm.nih.gov/geo/query/acc.cgi?acc=GSE6532 | 17401012 |
| 102 | Breast cancer | GSE6532_GPL96 | 12,547 | 138 | Affymetrix Human Genome U133A Array | 2019 | https://www.ncbi.nlm.nih.gov/geo/query/acc.cgi?acc=GSE6532 | 17401012 |
| 103 | Breast cancer | GSE7390 | 12,547 | 198 | Affymetrix Human Genome U135A Array | 2018 | https://www.ncbi.nlm.nih.gov/geo/query/acc.cgi?acc=GSE7390 | 17545524 |
| 104 | Breast cancer | GSE9195 | 21,653 | 77 | Affymetrix Human Genome U133 Plus 2.0 Array | 2019 | https://www.ncbi.nlm.nih.gov/geo/query/acc.cgi?acc=GSE9195 | 18498629 |
| 105 | Breast cancer | ICGC_BRCA_FR | 20,039 | 99 | Affymetrix Human U133 Plus 2.0 | 2019 | https://dcc.icgc.org/releases/current/Projects/BRCA-FR | NA |
| 106 | Breast cancer | ICGC_BRCA_KR | 26,730 | 50 | HTSeq | 2019 | https://dcc.icgc.org/releases/current/Projects/BRCA-KR | NA |
| 107 | Breast cancer | GSE46563 | 25,438 | 94 | Illumina HumanWG-6 v3.0 expression beadchip | 2019 | https://www.ncbi.nlm.nih.gov/geo/query/acc.cgi?acc=GSE46563 | 24599057 |
| 108 | Breast cancer | TCGA_BRCA | 34,849 | 1050 | TCGA-BRCA | 2021 | https://www.ncbi.nlm.nih.gov/geo/query/acc.cgi?acc= | 23000897 |
| 109 | Breast cancer | METABRIC | 25,233 | 2136 | METABRIC | 2021 | https://ega-archive.org/studies/EGAS00000000083 | 22522925 |
| 110 | Breast cancer | NCI | 4111 | 99 | In-house cDNA | 2003 | http://www.ncbi.nlm.nih.gov/pubmed/?term=12917485 | 12917485 |
| 111 | Breast cancer | NKI | 13,114 | 337 | Agilent | 2002 | http://www.ncbi.nlm.nih.gov/pubmed/?term=12490681；http://www.ncbi.nlm.nih.gov/pubmed/?term=11823860 | 12490681, 11823860 |
| 112 | Breast cancer | UCSF | 6497 | 162 | In-house cDNA | 2007 | http://www.ncbi.nlm.nih.gov/pubmed/?term=17428335 | 17428335, 14612510 |
| 113 | Burkitt's lymphoma | GSE4475 | 12,547 | 221 | Affymetrix Human Genome U133A Array | 2018 | https://www.ncbi.nlm.nih.gov/geo/query/acc.cgi?acc=GSE4475 | 16760442 |
| 114 | Cervical cancer | GSE44001 | 20,818 | 300 | Illumina HumanHT-12 WG-DASL V4.0 R2 expression beadchip | 2017 | https://www.ncbi.nlm.nih.gov/geo/query/acc.cgi?acc=GSE44001 | 24145113 |
| 115 | Cervical cancer | TCGA_CESC | 34,849 | 283 | HTSeq | 2021 | https://portal.gdc.cancer.gov/projectsTCGA-CESC | 28112728 |
| 116 | Cholangiocarcinoma | E_MTAB_6389 | 25,359 | 109 | Affymetrix GeneChip HTA-2_0 - Exon Level - HTA-2_0.r1.PsrsJucs.ps probesets | 2019 | https://www.ebi.ac.uk/arrayexpress/experiments/E-MTAB-6389 | NA |
| 117 | Cholangiocarcinoma | TCGA_CHOL | 34,849 | 36 | HTSeq | 2021 | https://portal.gdc.cancer.gov/projectsTCGA-CHOL | 28658632 |
| 118 | Chromophobe renal cell carcinoma | TCGA_KICH | 34,849 | 64 | HTSeq | 2021 | https://portal.gdc.cancer.gov/projectsTCGA-KICH | 25155756 |
| 119 | Chronic lymphocytic leukemia | GSE22762_GPL570 | 21,653 | 107 | Affymetrix Human Genome U133 Plus 2.0 Array | 2019 | https://www.ncbi.nlm.nih.gov/geo/query/acc.cgi?acc=GSE22762 | 21625232 |
| 120 | Chronic lymphocytic leukemia | GSE22762_GPL96 | 12,547 | 44 | Affymetrix Human Genome U133A Array | 2019 | https://www.ncbi.nlm.nih.gov/geo/query/acc.cgi?acc=GSE22762 | 21625232 |
| 121 | Chronic lymphocytic leukemia | GSE22762_GPL97 | 10,603 | 44 | Affymetrix Human Genome U133B Array | 2019 | https://www.ncbi.nlm.nih.gov/geo/query/acc.cgi?acc=GSE22762 | 21625232 |
| 122 | Colon cancer | GSE28722 | 15,240 | 125 | Rosetta custom human 23K array | 2012 | https://www.ncbi.nlm.nih.gov/geo/query/acc.cgi?acc=GSE28722 | 21251323 |
| 123 | Colon cancer | E_MTAB_863 | 3102 | 212 | Affymetrix Custom Array - Almac Diagnostics Colorectal Cancer DSATM research tool ADXCRCG2a520319 | 2014 | https://www.ebi.ac.uk/arrayexpress/experiments/E-MTAB-863 | 22067406 |
| 124 | Colon cancer | E_MTAB_864 | 3102 | 144 | Affymetrix Custom Array - Almac Diagnostics Colorectal Cancer DSATM research tool ADXCRCG2a520319 | 2014 | https://www.ebi.ac.uk/arrayexpress/experiments/E-MTAB-864 | 22067406 |
| 125 | Colon cancer | GSE16125_GPL5175 | 497 | 36 | Affymetrix Human Exon 1.0 ST Array | 2019 | https://www.ncbi.nlm.nih.gov/geo/query/acc.cgi?acc=GSE16125 | 19672874 |
| 126 | Colon cancer | GSE17536 | 21,653 | 177 | Affymetrix Human Genome U133 Plus 2.0 Array | 2020 | https://www.ncbi.nlm.nih.gov/geo/query/acc.cgi?acc=GSE17536 | 19914252 |
| 127 | Colon cancer | GSE17537 | 21,653 | 55 | Affymetrix Human Genome U133 Plus 2.0 Array | 2020 | https://www.ncbi.nlm.nih.gov/geo/query/acc.cgi?acc=GSE17537 | 19914252 |
| 128 | Colon cancer | GSE29621 | 21,653 | 65 | Affymetrix Human Genome U133 Plus 2.0 Array | 2019 | https://www.ncbi.nlm.nih.gov/geo/query/acc.cgi?acc=GSE29621 | 22362069 |
| 129 | Colon cancer | GSE31595 | 21,653 | 37 | Affymetrix Human Genome U133 Plus 2.0 Array | 2019 | https://www.ncbi.nlm.nih.gov/geo/query/acc.cgi?acc=GSE31595 | 22710688 |
| 130 | Colon cancer | GSE38832 | 21,653 | 122 | Affymetrix Human Genome U133 Plus 2.0 Array | 2019 | https://www.ncbi.nlm.nih.gov/geo/query/acc.cgi?acc=GSE38832 | 25320007 |
| 131 | Colon cancer | GSE39582 | 21,653 | 585 | Affymetrix Human Genome U133 Plus 2.0 Array | 2021 | https://www.ncbi.nlm.nih.gov/geo/query/acc.cgi?acc=GSE39582 | 23700391 |
| 132 | Colon cancer | TCGA_COAD | 34,849 | 430 | HTSeq | 2021 | https://portal.gdc.cancer.gov/projectsTCGA-COAD | 22810696 |
| 133 | Colorectal cancer | GSE12945 | 12,547 | 62 | Affymetrix Human Genome U134A Array | 2018 | https://www.ncbi.nlm.nih.gov/geo/query/acc.cgi?acc=GSE12945 | 19399471 |
| 134 | Colorectal cancer | GSE14333 | 21,653 | 290 | Affymetrix Human Genome U133 Plus 2.0 Array | 2019 | https://www.ncbi.nlm.nih.gov/geo/query/acc.cgi?acc=GSE14333 | 19996206 |
| 135 | Colorectal cancer | GSE30378 | 497 | 95 | Affymetrix Human Exon 1.0 ST Array | 2019 | https://www.ncbi.nlm.nih.gov/geo/query/acc.cgi?acc=GSE30378 | 22213796 |
| 136 | Colorectal cancer | GSE41258 | 12,547 | 182 | Affymetrix Human Genome U134A Array | 2019 | https://www.ncbi.nlm.nih.gov/geo/query/acc.cgi?acc=GSE41258 | 19359472 |
| 137 | Colorectal cancer | GSE24549_GPL5175 | 497 | 83 | Affymetrix Human Exon 1.0 ST Array | 2019 | https://www.ncbi.nlm.nih.gov/geo/query/acc.cgi?acc=GSE24549 | 21619627 |
| 138 | Colorectal cancer | GSE24550_GPL5175 | 497 | 90 | Affymetrix Human Exon 1.0 ST Array | 2019 | https://www.ncbi.nlm.nih.gov/geo/query/acc.cgi?acc=GSE24550 | 21619627 |
| 139 | Cutaneous melanoma | TCGA_SKCM | 34,849 | 98 | HTSeq | 2021 | https://portal.gdc.cancer.gov/projectsTCGA-SKCM | 26091043 |
| 140 | Diffuse large B cell lymphoma | E_MEXP_3488 | 20,088 | 43 | Affymetrix GeneChip Human Exon 1.0 ST Array version 1 | 2014 | https://www.ebi.ac.uk/arrayexpress/experiments/E-MEXP-3488 | 25381134 |
| 141 | Diffuse large B cell lymphoma | E_TABM_346 | 12,547 | 53 | Affymetrix GeneChip Human Genome HG-U133A | 2014 | https://www.ebi.ac.uk/arrayexpress/experiments/E-TABM-346 | 18615101 |
| 142 | Diffuse large B cell lymphoma | GSE10846 | 21,653 | 420 | Affymetrix Human Genome U133 Plus 2.0 Array | 2019 | https://www.ncbi.nlm.nih.gov/geo/query/acc.cgi?acc=GSE10846 | 19038878 |
| 143 | Diffuse large B cell lymphoma | GSE23501 | 21,653 | 69 | Affymetrix Human Genome U133 Plus 2.0 Array | 2019 | https://www.ncbi.nlm.nih.gov/geo/query/acc.cgi?acc=GSE23501 | 20610814 |
| 144 | Diffuse large B cell lymphoma | TCGA_DLBC | 34,849 | 46 | HTSeq | 2021 | https://portal.gdc.cancer.gov/projectsTCGA-DLBC | 29641966 |
| 145 | Endometrial Carcinoma | TCGA_UCEC | 34,849 | 533 | HTSeq | 2021 | https://portal.gdc.cancer.gov/projectsTCGA-UCEC | 23636398 |
| 146 | Esophageal adenocarcinomas | GSE19417 | 19,882 | 70 | Rosetta/Merck Human 44k 1.1 microarray | 2012 | https://www.ncbi.nlm.nih.gov/geo/query/acc.cgi?acc=GSE19417 | 20621683 |
| 147 | Esophageal adenocarcinomas | TCGA_ESCA | 34,849 | 151 | HTSeq | 2021 | https://portal.gdc.cancer.gov/projectsTCGA-ESCA | 28052061 |
| 148 | Follicular lymphoma | GSE16131_GPL96 | 12,547 | 184 | Affymetrix Human Genome U133A Array | 2018 | https://www.ncbi.nlm.nih.gov/geo/query/acc.cgi?acc=GSE16131 | 19471018 |
| 149 | Follicular lymphoma | GSE16131_GPL97 | 10,603 | 184 | Affymetrix Human Genome U133B Array | 2018 | https://www.ncbi.nlm.nih.gov/geo/query/acc.cgi?acc=GSE16131 | 19471018 |
| 150 | Gastric cancer | GSE26253 | 13,509 | 432 | Illumina HumanRef-8 WG-DASL v3.0 | 2019 | https://www.ncbi.nlm.nih.gov/geo/query/acc.cgi?acc=GSE26253 | 24598828 |
| 151 | Gastric cancer | GSE15459 | 21,653 | 192 | Affymetrix Human Genome U133 Plus 2.0 Array | 2019 | https://www.ncbi.nlm.nih.gov/geo/query/acc.cgi?acc=GSE15459 | 19798449 |
| 152 | Gastric cancer | GSE34942 | 21,653 | 56 | Affymetrix Human Genome U133 Plus 2.0 Array | 2019 | https://www.ncbi.nlm.nih.gov/geo/query/acc.cgi?acc=GSE34942 | 25053715 |
| 153 | Gastric cancer | GSE62254 | 21,653 | 300 | Affymetrix Human Genome U133 Plus 2.0 Array | 2019 | https://www.ncbi.nlm.nih.gov/geo/query/acc.cgi?acc=GSE62254 | 25894828 |
| 154 | Gastric cancer | TCGA_STAD | 34,849 | 348 | HTSeq | 2021 | https://portal.gdc.cancer.gov/projectsTCGA-STAD | 25079317 |
| 155 | Head and neck squamous cell carcinoma | GSE10300 | 21,653 | 44 | Affymetrix Human Genome U133 Plus 2.0 Array | 2019 | https://www.ncbi.nlm.nih.gov/geo/query/acc.cgi?acc=GSE10300 | 19117988 |
| 156 | Head and neck squamous cell carcinoma | GSE65858 | 22,000 | 270 | Illumina HumanHT-12 V4.0 expression beadchip | 2018 | https://www.ncbi.nlm.nih.gov/geo/query/acc.cgi?acc=GSE65858 | 26095926 |
| 157 | Head and neck squamous cell carcinoma | E_MTAB_1328 | 21,653 | 89 | Affymetrix Human Genome U133 Plus 2.0 Array | 2014 | https://www.ebi.ac.uk/arrayexpress/experiments/E-MTAB-1328 | 23757353 |
| 158 | Head and neck squamous cell carcinoma | TCGA_HNSC | 34,849 | 494 | HTSeq | 2021 | https://portal.gdc.cancer.gov/projectsTCGA-HNSC | 25631445 |
| 159 | Hepatocellular Carcinoma | ICGC_LIRI_JP | 22,913 | 445 | HTSeq | 2019 | https://dcc.icgc.org/releases/current/Projects/LIRI-JP | NA |
| 160 | Hepatocellular Carcinoma | GSE10141 | 6100 | 80 | Human 6k Transcriptionally Informative Gene Panel for DASL | 2020 | https://www.ncbi.nlm.nih.gov/geo/query/acc.cgi?acc=GSE10141 | 18923165 |
| 161 | Hepatocellular Carcinoma | GSE17856 | 14,313 | 52 | Agilent-014850 Whole Human Genome Microarray 4x44K G4112F | 2019 | https://www.ncbi.nlm.nih.gov/geo/query/acc.cgi?acc=GSE17856 | 20380719 |
| 162 | Hepatocellular Carcinoma | GSE27150 | 2348 | 81 | State Key Lab Homo sapien 2.6K | 2012 | https://www.ncbi.nlm.nih.gov/geo/query/acc.cgi?acc=GSE27150 | NA |
| 163 | Hepatocellular Carcinoma | GSE14520 | 12,742 | 221 | Affymetrix HT Human Genome U133A Array | 2021 | https://www.ncbi.nlm.nih.gov/geo/query/acc.cgi?acc=GSE14520 | 21159642 |
| 164 | Hepatocellular Carcinoma | TCGA_LIHC | 34,849 | 363 | HTSeq | 2021 | https://portal.gdc.cancer.gov/projectsTCGA-LIHC | 28622513 |
| 165 | Laryngeal cancer | GSE27020 | 12,547 | 109 | Affymetrix Human Genome U133A Array | 2018 | https://www.ncbi.nlm.nih.gov/geo/query/acc.cgi?acc=GSE27020 | 23950933 |
| 166 | Lung cancer | GSE41271 | 25,438 | 275 | Illumina HumanWG-6 v3.0 expression beadchip | 2019 | https://www.ncbi.nlm.nih.gov/geo/query/acc.cgi?acc=GSE41271 | 23449933 |
| 167 | Lung cancer | GSE3141 | 21,653 | 111 | Affymetrix Human Genome U133 Plus 2.0 Array | 2019 | https://www.ncbi.nlm.nih.gov/geo/query/acc.cgi?acc=GSE3141 | 16273092 |
| 168 | Lung cancer | GSE30219 | 21,653 | 307 | Affymetrix Human Genome U133 Plus 2.0 Array | 2019 | https://www.ncbi.nlm.nih.gov/geo/query/acc.cgi?acc=GSE30219 | 23698379 |
| 169 | Lung cancer | GSE31547 | 12,547 | 50 | Affymetrix Human Genome U133A Array | 2018 | https://www.ncbi.nlm.nih.gov/geo/query/acc.cgi?acc=GSE31547 | NA |
| 170 | Lung cancer-Lung adenocarcinoma | GSE26939 | 17,108 | 116 | Agilent-UNC-custom-4X44K | 2012 | https://www.ncbi.nlm.nih.gov/geo/query/acc.cgi?acc=GSE26939 | 22590557 |
| 171 | Lung cancer-Lung adenocarcinoma | GSE72094 | 22,115 | 442 | Rosetta/Merck Human RSTA Custom Affymetrix 2.0 microarray | 2018 | https://www.ncbi.nlm.nih.gov/geo/query/acc.cgi?acc=GSE72094 | 26477306 |
| 172 | Lung cancer-Lung adenocarcinoma | GSE13213 | 18,479 | 117 | Agilent-014850 Whole Human Genome Microarray 4x44K G4112F | 2019 | https://www.ncbi.nlm.nih.gov/geo/query/acc.cgi?acc=GSE13213 | 19414676 |
| 173 | Lung cancer-Lung adenocarcinoma | GSE11969 | 16,624 | 163 | Agilent Homo sapiens 21.6K custom array | 2013 | https://www.ncbi.nlm.nih.gov/geo/query/acc.cgi?acc=GSE11969 | 16549822 |
| 174 | Lung cancer-Lung adenocarcinoma | GSE5843 | 13,111 | 48 | PRHU05-S1-0006 (PC Human Operon v2 21k) | 2015 | https://www.ncbi.nlm.nih.gov/geo/query/acc.cgi?acc=GSE5843 | 17504995 |
| 175 | Lung cancer-Lung adenocarcinoma | E_MTAB_923 | 21,653 | 103 | Affymetrix GeneChip Human Genome U133 Plus 2.0 | 2014 | https://www.ebi.ac.uk/arrayexpress/experiments/E-MTAB-923 | 22914773 |
| 176 | Lung cancer-Lung adenocarcinoma | GSE31210 | 21,653 | 246 | Affymetrix Human Genome U133 Plus 2.0 Array | 2019 | https://www.ncbi.nlm.nih.gov/geo/query/acc.cgi?acc=GSE31210 | 22080568 |
| 177 | Lung cancer-Lung adenocarcinoma | GSE68465 | 12,547 | 462 | Affymetrix Human Genome U133A Array | 2018 | https://www.ncbi.nlm.nih.gov/geo/query/acc.cgi?acc=GSE68465 | 18641660 |
| 178 | Lung cancer-Lung adenocarcinoma | GSE68571 | 5249 | 96 | Affymetrix Human Full Length HuGeneFL Array | 2016 | https://www.ncbi.nlm.nih.gov/geo/query/acc.cgi?acc=GSE68571 | 12118244 |
| 179 | Lung cancer-Lung adenocarcinoma | GSE63459 | 18,630 | 65 | Illumina HumanRef-8 v3.0 expression beadchip | 2017 | https://www.ncbi.nlm.nih.gov/geo/query/acc.cgi?acc=GSE63459 | 26134223 |
| 180 | Lung cancer-Lung adenocarcinoma | TCGA_LUAD | 34,849 | 497 | HTSeq | 2021 | https://portal.gdc.cancer.gov/projectsTCGA-LUAD | 25079552 |
| 181 | Lung cancer-Lung squamous cell carcinoma | GSE17710 | 17,108 | 56 | Agilent-UNC-custom-4X44K | 2012 | https://www.ncbi.nlm.nih.gov/geo/query/acc.cgi?acc=GSE17710 | 20643781 |
| 182 | Lung cancer-Lung squamous cell carcinoma | E_MTAB_2435 | 21,653 | 93 | Affymetrix GeneChip Human Genome U133 Plus 2.0 | 2018 | https://www.ebi.ac.uk/arrayexpress/experiments/E-MTAB-2435 | 25189482 |
| 183 | Lung cancer-Non-small cell lung cancer | GSE11117 | 9661 | 56 | Novachip human 34.5k | 2012 | https://www.ncbi.nlm.nih.gov/geo/query/acc.cgi?acc=GSE11117 | 19833826 |
| 184 | Lung cancer-Non-small cell lung cancer | GSE8894 | 21,653 | 138 | Affymetrix Human Genome U133 Plus 2.0 Array | 2019 | https://www.ncbi.nlm.nih.gov/geo/query/acc.cgi?acc=GSE8894 | 19010856 |
| 185 | Lung cancer-Non-small cell lung cancer | GSE157009 | 21,653 | 249 | Affymetrix Human Genome U133 Plus 2.0 Array | 2020 | https://www.ncbi.nlm.nih.gov/geo/query/acc.cgi?acc=GSE157009 | 32717408 |
| 186 | Lung cancer-Non-small cell lung cancer | GSE4573 | 12,547 | 129 | Affymetrix Human Genome U133A Array | 2018 | https://www.ncbi.nlm.nih.gov/geo/query/acc.cgi?acc=GSE4573 | 16885343 |
| 187 | Lung cancer-Non-small cell lung cancer | GSE42127 | 25,438 | 176 | Illumina HumanWG-6 v3.0 expression beadchip | 2020 | https://www.ncbi.nlm.nih.gov/geo/query/acc.cgi?acc=GSE42127 | 23357979 |
| 188 | Lung cancer-Non-small cell lung cancer | GSE14814 | 12,547 | 133 | Affymetrix Human Genome U133A Array | 2018 | https://www.ncbi.nlm.nih.gov/geo/query/acc.cgi?acc=GSE14814 | 20823422 |
| 189 | Lung cancer-Non-small cell lung cancer | GSE157010 | 21,653 | 235 | Affymetrix Human Genome U133 Plus 2.0 Array | 2020 | https://www.ncbi.nlm.nih.gov/geo/query/acc.cgi?acc=GSE157010 | 32717408 |
| 190 | Lung cancer-Non-small cell lung cancer | GSE19188 | 21,653 | 156 | Affymetrix Human Genome U133 Plus 2.0 Array | 2019 | https://www.ncbi.nlm.nih.gov/geo/query/acc.cgi?acc=GSE19188 | 20421987 |
| 191 | Lung cancer-Non-small cell lung cancer | GSE37745 | 21,653 | 196 | Affymetrix Human Genome U133 Plus 2.0 Array | 2021 | https://www.ncbi.nlm.nih.gov/geo/query/acc.cgi?acc=GSE37745 | 23032747 |
| 192 | Lung cancer-Non-small cell lung cancer | GSE50081 | 21,653 | 181 | Affymetrix Human Genome U133 Plus 2.0 Array | 2019 | https://www.ncbi.nlm.nih.gov/geo/query/acc.cgi?acc=GSE50081 | 24305008 |
| 193 | Lung cancer-Non-small cell lung cancer | TCGA_LUSC | 34,849 | 489 | HTSeq | 2021 | https://portal.gdc.cancer.gov/projectsTCGA-LUSC | 22960745 |
| 194 | Mesothelioma | E_MTAB_1719 | 21,653 | 38 | Affymetrix GeneChip Human Genome U133 Plus 2.0 | 2018 | https://www.ebi.ac.uk/arrayexpress/experiments/E-MTAB-1719 | 24443521 |
| 195 | Mesothelioma | E_MTAB_6877 | 15,653 | 67 | Affymetrix Human Gene 2.0 ST Array | 2020 | https://www.ebi.ac.uk/arrayexpress/experiments/E-MTAB-6877 | 30902996 |
| 196 | Mesothelioma | TCGA_MESO | 34,849 | 79 | HTSeq | 2021 | https://portal.gdc.cancer.gov/projectsTCGA-MESO | 30322867 |
| 197 | Multiple myeloma | GSE9782_GPL96 | 12,547 | 264 | Affymetrix Human Genome U133A Array | 2018 | https://www.ncbi.nlm.nih.gov/geo/query/acc.cgi?acc=GSE9782 | 17185464 |
| 198 | Multiple myeloma | GSE9782_GPL97 | 10,603 | 264 | Affymetrix Human Genome U133B Array | 2018 | https://www.ncbi.nlm.nih.gov/geo/query/acc.cgi?acc=GSE9782 | 17185464 |
| 199 | Multiple myeloma | E_MTAB_1038 | 20,202 | 73 | Affymetrix GeneChip Human Gene 1.0 ST Array | 2014 | https://www.ebi.ac.uk/arrayexpress/experiments/E-MTAB-1038 | 27234807 |
| 200 | Multiple myeloma | E_MTAB_4032 | 20,202 | 151 | Affymetrix Human Gene 1.0 ST Array | 2016 | https://www.ebi.ac.uk/arrayexpress/experiments/E-MTAB-4032 | 27234807 |
| 201 | Multiple myeloma | GSE136337 | 21,653 | 426 | Affymetrix Human Genome U133 Plus 2.0 Array | 2020 | https://www.ncbi.nlm.nih.gov/geo/query/acc.cgi?acc=GSE136337 | 33147277 |
| 202 | Multiple myeloma | GSE24080 | 21,653 | 559 | Affymetrix Human Genome U133 Plus 2.0 Array | 2019 | https://www.ncbi.nlm.nih.gov/geo/query/acc.cgi?acc=GSE24080 | 20064235 |
| 203 | Multiple myeloma | GSE57317 | 21,653 | 55 | Affymetrix Human Genome U133 Plus 2.0 Array | 2019 | https://www.ncbi.nlm.nih.gov/geo/query/acc.cgi?acc=GSE57317 | 25079174 |
| 204 | Multiple myeloma | MMRF_CoMMpass | 34,849 | 787 | HTSeq | 2018 | https://portal.gdc.cancer.gov/projects | 27417553 |
| 205 | Neuroblastoma | E_MTAB_8248 | 19,860 | 223 | Agilent-020382 Human Custom Microarray 44k | 2019 | https://www.ebi.ac.uk/arrayexpress/experiments/E-MTAB-8248 | 32291317 |
| 206 | Neuroblastoma | TARGET_NBL | 58,387 | 142 | Illumina HiSeq | 2019 | https://xenabrowser.net/datapages/ | 23334666 |
| 207 | Neuroblastoma | GSE62564 | 23,465 | 498 | Illumina HiSeq 2000 (Homo sapiens) | 2019 | https://www.ncbi.nlm.nih.gov/geo/query/acc.cgi?acc=GSE62564 | 25150839 |
| 208 | Oral cancer | ICGC_ORCA_IN | 24,003 | 40 | HTSeq | 2019 | https://dcc.icgc.org/releases/current/Projects/ORCA-IN | NA |
| 209 | Osteosarcoma | TARGET_OS | 58,387 | 87 | Illumina HiSeq | 2019 | https://xenabrowser.net/datapages/ | 29228567 |
| 210 | Osteosarcoma | GSE21257 | 24,996 | 53 | Illumina human-6 v2.0 expression beadchip (using nuIDs as identifier) | 2012 | https://www.ncbi.nlm.nih.gov/geo/query/acc.cgi?acc=GSE21257 | 21372215 |
| 211 | Ovarian cancer | GSE17260 | 19,595 | 110 | Agilent-014850 Whole Human Genome Microarray 4x44K G4112F (Probe Name version) | 2019 | https://www.ncbi.nlm.nih.gov/geo/query/acc.cgi?acc=GSE17260 | 20300634 |
| 212 | Ovarian cancer | ICGC_OV_AU | 24,650 | 111 | HTSeq | 2019 | https://dcc.icgc.org/releases/current/Projects/OV-AU | NA |
| 213 | Ovarian cancer | GSE73614 | 19,584 | 107 | Agilent-014850 Whole Human Genome Microarray 4x44K G4112F | 2019 | https://www.ncbi.nlm.nih.gov/geo/query/acc.cgi?acc=GSE73614 | 27016234 |
| 214 | Ovarian cancer | GSE49997 | 16,751 | 204 | ABI Human Genome Survey Microarray Version 2 | 2016 | https://www.ncbi.nlm.nih.gov/geo/query/acc.cgi?acc=GSE49997 | 22497737 |
| 215 | Ovarian cancer | GSE13876 | 15,971 | 415 | Operon human v3 ~35K 70-mer two-color oligonucleotide microarrays | 2013 | https://www.ncbi.nlm.nih.gov/geo/query/acc.cgi?acc=GSE13876 | 19192944 |
| 216 | Ovarian cancer | GSE14764 | 12,547 | 80 | Affymetrix Human Genome U133A Array | 2019 | https://www.ncbi.nlm.nih.gov/geo/query/acc.cgi?acc=GSE14764 | 19294737 |
| 217 | Ovarian cancer | GSE18520 | 21,653 | 63 | Affymetrix Human Genome U133 Plus 2.0 Array | 2019 | https://www.ncbi.nlm.nih.gov/geo/query/acc.cgi?acc=GSE18520 | 19962670 |
| 218 | Ovarian cancer | GSE19829_GPL570 | 21,653 | 28 | Affymetrix Human Genome U133 Plus 2.0 Array | 2019 | https://www.ncbi.nlm.nih.gov/geo/query/acc.cgi?acc=GSE19829 | 20547991 |
| 219 | Ovarian cancer | GSE19829_GPL8300 | 8619 | 42 | Affymetrix Human Genome U95 Version 2 Array | 2019 | https://www.ncbi.nlm.nih.gov/geo/query/acc.cgi?acc=GSE19829 | 20547991 |
| 220 | Ovarian cancer | GSE23554 | 12,547 | 28 | Affymetrix Human Genome U133A Array | 2018 | https://www.ncbi.nlm.nih.gov/geo/query/acc.cgi?acc=GSE23554 | 21849418 |
| 221 | Ovarian cancer | GSE26712 | 87 | 195 | Affymetrix Human Genome U134A Array | 2018 | https://www.ncbi.nlm.nih.gov/geo/query/acc.cgi?acc=GSE26712 | 18593951 |
| 222 | Ovarian cancer | GSE30161 | 21,653 | 58 | Affymetrix Human Genome U133 Plus 2.0 Array | 2019 | https://www.ncbi.nlm.nih.gov/geo/query/acc.cgi?acc=GSE30161 | 22348014 |
| 223 | Ovarian cancer | GSE31245 | 8619 | 57 | Affymetrix Human Genome U95 Version 2 Array | 2018 | https://www.ncbi.nlm.nih.gov/geo/query/acc.cgi?acc=GSE31245 | 16204010 |
| 224 | Ovarian cancer | GSE63885 | 21,653 | 101 | Affymetrix Human Genome U133 Plus 2.0 Array | 2019 | https://www.ncbi.nlm.nih.gov/geo/query/acc.cgi?acc=GSE63885 | 24478986 |
| 225 | Ovarian cancer | GSE9891 | 21,653 | 285 | Affymetrix Human Genome U133 Plus 2.0 Array | 2019 | https://www.ncbi.nlm.nih.gov/geo/query/acc.cgi?acc=GSE9891 | 18698038 |
| 226 | Ovarian cancer | GSE32062_GPL6480 | 19,595 | 260 | Agilent-014850 Whole Human Genome Microarray 4x44K G4112F (Probe Name version) | 2019 | https://www.ncbi.nlm.nih.gov/geo/query/acc.cgi?acc=GSE32062 | 22241791 |
| 227 | Ovarian cancer | GSE32063 | 19,595 | 40 | Agilent-014850 Whole Human Genome Microarray 4x44K G4112F | 2019 | https://www.ncbi.nlm.nih.gov/geo/query/acc.cgi?acc=GSE32063 | 22241791 |
| 228 | Ovarian cancer | TCGA_OV | 34,849 | 353 | HTSeq | 2021 | https://portal.gdc.cancer.gov/projectsTCGA-OV | 21720365 |
| 229 | Pancreatic ductal adenocarcinoma | ICGC_PACA_CA | 25,731 | 262 | HTSeq | 2019 | https://dcc.icgc.org/releases/current/Projects/PACA-CA | NA |
| 230 | Pancreatic endocrine neoplasms | ICGC_PAEN_AU | 24,650 | 33 | HTSeq | 2019 | https://dcc.icgc.org/releases/current/Projects/PAEN-AU | NA |
| 231 | Pancreatic ductal adenocarcinoma | GSE21501 | 19,749 | 132 | Agilent-014850 Whole Human Genome Microarray 4x44K G4112F | 2013 | https://www.ncbi.nlm.nih.gov/geo/query/acc.cgi?acc=GSE21501 | 20644708 |
| 232 | Pancreatic ductal adenocarcinoma | ICGC_PACA_AU | 24,650 | 92 | HTSeq | 2019 | https://dcc.icgc.org/releases/current/Projects/PACA-AU | 21436628 |
| 233 | Pancreatic ductal adenocarcinoma | GSE71729 | 19,749 | 357 | Agilent-014850 Whole Human Genome Microarray 4x44K G4112F | 2015 | https://www.ncbi.nlm.nih.gov/geo/query/acc.cgi?acc=GSE71729 | 26343385 |
| 234 | pancreatic ductal adenocarcinoma | E_MEXP_2780 | 21,653 | 26 | Affymetrix GeneChip Human Genome U133 Plus 2.0 | 2014 | https://www.ebi.ac.uk/arrayexpress/experiments/E-MEXP-2780 | 22615549 |
| 235 | pancreatic ductal adenocarcinoma | E_MTAB_6134 | 19,040 | 309 | Affymetrix Human Genome U219 Array | 2018 | https://www.ebi.ac.uk/arrayexpress/experiments/E-MTAB-6134 | 30165049 |
| 236 | Pancreatic ductal adenocarcinoma | GSE28735 | 20,202 | 90 | Affymetrix Human Gene 1.0 ST Array | 2018 | https://www.ncbi.nlm.nih.gov/geo/query/acc.cgi?acc=GSE28735 | 22363658 |
| 237 | Pancreatic ductal adenocarcinoma | GSE57495 | 22,115 | 63 | Rosetta/Merck Human RSTA Custom Affymetrix 2.0 microarray | 2018 | https://www.ncbi.nlm.nih.gov/geo/query/acc.cgi?acc=GSE57495 | 26247463 |
| 238 | Pancreatic ductal adenocarcinoma | GSE62452 | 20,202 | 130 | Affymetrix Human Gene 1.0 ST Array | 2018 | https://www.ncbi.nlm.nih.gov/geo/query/acc.cgi?acc=GSE62452 | 27197190 |
| 239 | Pancreatic ductal adenocarcinoma | GSE78229 | 20,202 | 50 | Affymetrix Human Gene 1.0 ST Array | 2018 | https://www.ncbi.nlm.nih.gov/geo/query/acc.cgi?acc=GSE78229 | 27401251 |
| 240 | Pancreatic ductal adenocarcinoma | GSE85916 | 19,040 | 80 | Affymetrix Human Genome U219 Array | 2019 | https://www.ncbi.nlm.nih.gov/geo/query/acc.cgi?acc=GSE85916 | NA |
| 241 | Pancreatic ductal adenocarcinoma | GSE79668 | 24,658 | 51 | Illumina HiSeq 2000 (Homo sapiens) | 2019 | https://www.ncbi.nlm.nih.gov/geo/query/acc.cgi?acc=GSE79668 | 27282075 |
| 242 | Pancreatic ductal adenocarcinoma | TCGA_PAAD | 34,849 | 176 | HTSeq | 2021 | https://portal.gdc.cancer.gov/projectsTCGA-PAAD | 28810144 |
| 243 | Pheochromocytoma and paraganglioma | TCGA_PCPG | 34,849 | 175 | HTSeq | 2021 | https://portal.gdc.cancer.gov/projectsTCGA-PCPG | 28162975 |
| 244 | Prostate cancer | GSE70768 | 22,000 | 199 | Illumina HumanHT-12 V4.0 expression beadchip | 2018 | https://www.ncbi.nlm.nih.gov/geo/query/acc.cgi?acc=GSE70768 | 26501111 |
| 245 | Prostate cancer | GSE70769 | 22,000 | 94 | Illumina HumanHT-12 V4.0 expression beadchip | 2018 | https://www.ncbi.nlm.nih.gov/geo/query/acc.cgi?acc=GSE70769 | 26501111 |
| 246 | Prostate cancer | GSE16560 | 6100 | 281 | Human 6k Transcriptionally Informative Gene Panel for DASL | 2013 | https://www.ncbi.nlm.nih.gov/geo/query/acc.cgi?acc=GSE16560 | 20233430 |
| 247 | Prostate cancer | GSE26022 | 522 | 192 | Illumina Custom Prostate Cancer DASL Panel 1.5K expression beadchip | 2012 | https://www.ncbi.nlm.nih.gov/geo/query/acc.cgi?acc=GSE26022 | NA |
| 248 | Prostate cancer | GSE26242 | 522 | 96 | Illumina Custom Prostate Cancer DASL Panel 1.5K expression beadchip | 2012 | https://www.ncbi.nlm.nih.gov/geo/query/acc.cgi?acc=GSE26242 | NA |
| 249 | Prostate cancer | TCGA_PRAD | 34,849 | 481 | HTSeq | 2021 | https://portal.gdc.cancer.gov/projectsTCGA-PRAD | 26544944 |
| 250 | Prostate cancer | ICGC_PRAD_FR | 22,187 | 25 | HTSeq | 2019 | https://dcc.icgc.org/releases/current/Projects/PRAD-FR | NA |
| 251 | Rectum adenocarcinoma | TCGA_READ | 34,849 | 154 | HTSeq | 2021 | https://portal.gdc.cancer.gov/projectsTCGA-READ | 22810696 |
| 252 | Renal carcinoma | GSE167573 | 55,630 | 76 | HiSeq X Ten (Homo sapiens) | 2021 | https://www.ncbi.nlm.nih.gov/geo/query/acc.cgi?acc=GSE167573 | 34489456 |
| 253 | Renal carcinoma | ICGC_RECA_EU | 23,698 | 136 | HTSeq | 2019 | https://dcc.icgc.org/releases/current/Projects/RECA-EU | NA |
| 254 | Renal carcinoma-Clear-cell renal cell carcinoma | E_MTAB_1980 | 22,682 | 101 | Agilent Human Gene Expression 4x44K v2 Microarray 026652 G4845A | 2014 | https://www.ebi.ac.uk/arrayexpress/experiments/E-MTAB-1980 | 23797736 |
| 255 | Renal carcinoma-Clear-cell renal cell carcinoma | GSE29609 | 18,841 | 39 | Agilent-012391 Whole Human Genome Oligo Microarray G4112A | 2018 | https://www.ncbi.nlm.nih.gov/geo/query/acc.cgi?acc=GSE29609 | 22626276 |
| 256 | Renal carcinoma-Clear-cell renal cell carcinoma | E_MTAB_3218 | 19,040 | 59 | Affymetrix Human Genome U219 Array | 2021 | https://www.ebi.ac.uk/arrayexpress/experiments/E-MTAB-3218 | 27169994 |
| 257 | Renal carcinoma-Clear-cell renal cell carcinoma | E_MTAB_3267 | 20,202 | 59 | Affymetrix GeneChip Human Gene 1.0 ST Array | 2018 | https://www.ebi.ac.uk/arrayexpress/experiments/E-MTAB-3267 | 25583177 |
| 258 | Renal carcinoma-Clear-cell renal cell carcinoma | TCGA_KIRC | 34,849 | 522 | HTSeq | 2021 | https://portal.gdc.cancer.gov/projectsTCGA-KIRC | 23792563 |
| 259 | Renal carcinoma-Renal papillary cell carcinoma | TCGA_KIRP | 34,849 | 284 | HTSeq | 2021 | https://portal.gdc.cancer.gov/projectsTCGA-KIRP | 26536169 |
| 260 | Rhabdomyosarcoma | E_TABM_1202 | 21,653 | 101 | Affymetrix GeneChip Human Genome U133 Plus 2.0 | 2012 | https://www.ebi.ac.uk/arrayexpress/experiments/E-TABM-1202 | 23536298 |
| 261 | Testicular germ cell tumor | TCGA_TGCT | 34,849 | 133 | HTSeq | 2021 | https://portal.gdc.cancer.gov/projectsTCGA-TGCT | 29898407 |
| 262 | Thymoma | TCGA_THYM | 34,849 | 118 | HTSeq | 2021 | https://portal.gdc.cancer.gov/projectsTCGA-THYM | 29438696 |
| 263 | Thyroid carcinoma | TCGA_THCA | 34,849 | 496 | HTSeq | 2021 | https://portal.gdc.cancer.gov/projectsTCGA-THCA | 25417114 |
| 264 | Uterine carcinosarcoma | TCGA_UCS | 34,849 | 54 | HTSeq | 2021 | https://portal.gdc.cancer.gov/projectsTCGA-UCS | 28292439 |
| 265 | Uveal melanoma | GSE39717 | 21,453 | 31 | Illumina humanRef-8 v1.0 expression beadchip | 2015 | https://www.ncbi.nlm.nih.gov/geo/query/acc.cgi?acc=GSE39717 | 17332290 |
| 266 | Uveal melanoma | GSE22138 | 21,653 | 63 | Affymetrix Human Genome U133 Plus 2.0 Array | 2019 | https://www.ncbi.nlm.nih.gov/geo/query/acc.cgi?acc=GSE22138 | 21135111 |
| 267 | Uveal melanoma | TCGA_UVM | 34,849 | 77 | HTSeq | 2021 | https://portal.gdc.cancer.gov/projectsTCGA-UVM | 29316429 |
| 268 | Wilms tumor | TARGET_WT | 58,387 | 120 | Illumina HiSeq | 2019 | https://xenabrowser.net/datapages/ | 28825729 |

*Note*: PMID, PubMed Unique Identifier.
