# Supplementary material for: CBioProfiler: A Web and Standalone Pipeline for Cancer Biomarker and Subtype Characterization
Source: Genomics Proteomics Bioinformatics. 2024 Jun 12;22(3):qzae045. doi: 10.1093/gpbjnl/qzae045 (PMC11464420; doi:10.1093/gpbjnl/qzae045)
Supplement: qzae045_Supplementary_Data [file qzae045_supplementary_data.zip › Table S3-done.docx]

**Table S3 Univariate Cox proportional hazards regression model on the overall survival of patients with breast cancer in METABRIC cohort**

| **Genes** | **Coefficient** | **HR** | **LCI** | **UCI** | ***P* value** | ***P* adjusted** |
| --- | --- | --- | --- | --- | --- | --- |
| *RACGAP1* | 0.376 | 1.456 | 1.316 | 1.610 | < 0.001 | < 0.001 |
| *CDCA5* | 0.240 | 1.271 | 1.190 | 1.358 | < 0.001 | < 0.001 |
| *TROAP* | 0.319 | 1.376 | 1.260 | 1.502 | < 0.001 | < 0.001 |
| *STIP1* | 0.421 | 1.524 | 1.353 | 1.716 | < 0.001 | < 0.001 |
| *PKMYT1* | 0.381 | 1.464 | 1.313 | 1.631 | < 0.001 | < 0.001 |
| *UBE2C* | 0.189 | 1.208 | 1.144 | 1.275 | < 0.001 | < 0.001 |
| *TPX2* | 0.282 | 1.326 | 1.223 | 1.438 | < 0.001 | < 0.001 |
| *KIF20A* | 0.279 | 1.321 | 1.219 | 1.432 | < 0.001 | < 0.001 |
| *CFL1* | 0.559 | 1.749 | 1.485 | 2.060 | < 0.001 | < 0.001 |
| *SUSD3* | −0.142 | 0.868 | 0.833 | 0.905 | < 0.001 | < 0.001 |
| *CLIC6* | −0.106 | 0.900 | 0.872 | 0.928 | < 0.001 | < 0.001 |
| *FAM83D* | 0.272 | 1.313 | 1.211 | 1.424 | < 0.001 | < 0.001 |
| *LARP1* | 0.613 | 1.846 | 1.539 | 2.216 | < 0.001 | < 0.001 |
| *PTTG1* | 0.230 | 1.259 | 1.175 | 1.349 | < 0.001 | < 0.001 |
| *CPT1A* | 0.445 | 1.561 | 1.365 | 1.785 | < 0.001 | < 0.001 |
| *KIF4A* | 0.380 | 1.462 | 1.304 | 1.639 | < 0.001 | < 0.001 |
| *PIGV* | −0.480 | 0.619 | 0.535 | 0.715 | < 0.001 | < 0.001 |
| *AURKA* | 0.241 | 1.273 | 1.184 | 1.369 | < 0.001 | < 0.001 |
| *FCER1A* | −0.208 | 0.812 | 0.762 | 0.865 | < 0.001 | < 0.001 |
| *PPIL3* | −0.500 | 0.606 | 0.521 | 0.706 | < 0.001 | < 0.001 |
| *PLK1* | 0.531 | 1.701 | 1.445 | 2.003 | < 0.001 | < 0.001 |
| *PRC1* | 0.238 | 1.268 | 1.179 | 1.365 | < 0.001 | < 0.001 |
| *UHRF1* | 0.248 | 1.282 | 1.187 | 1.384 | < 0.001 | < 0.001 |
| *CKAP2L* | 0.317 | 1.373 | 1.244 | 1.516 | < 0.001 | < 0.001 |
| *PARP3* | −0.348 | 0.706 | 0.633 | 0.787 | < 0.001 | < 0.001 |
| *VEGFA* | 0.281 | 1.325 | 1.212 | 1.447 | < 0.001 | < 0.001 |
| *MELK* | 0.216 | 1.241 | 1.159 | 1.328 | < 0.001 | < 0.001 |
| *GTSE1* | 0.381 | 1.464 | 1.298 | 1.651 | < 0.001 | < 0.001 |
| *GSK3B* | 0.359 | 1.432 | 1.278 | 1.604 | < 0.001 | < 0.001 |
| *CENPO* | 0.606 | 1.832 | 1.510 | 2.224 | < 0.001 | < 0.001 |
| *FEN1* | 0.292 | 1.339 | 1.220 | 1.471 | < 0.001 | < 0.001 |
| *AK3* | −0.438 | 0.645 | 0.561 | 0.742 | < 0.001 | < 0.001 |
| *TUBA1B* | 0.483 | 1.620 | 1.387 | 1.893 | < 0.001 | < 0.001 |
| *CCNB2* | 0.209 | 1.232 | 1.152 | 1.318 | < 0.001 | < 0.001 |
| *TRIM4* | −0.377 | 0.686 | 0.607 | 0.775 | < 0.001 | < 0.001 |
| *SHMT2* | 0.359 | 1.432 | 1.275 | 1.609 | < 0.001 | < 0.001 |
| *NUSAP1* | 0.250 | 1.284 | 1.184 | 1.392 | < 0.001 | < 0.001 |
| *ENC1* | 0.319 | 1.376 | 1.241 | 1.525 | < 0.001 | < 0.001 |
| *CDC20* | 0.169 | 1.184 | 1.121 | 1.251 | < 0.001 | < 0.001 |
| *RBBP8* | −0.245 | 0.782 | 0.723 | 0.847 | < 0.001 | < 0.001 |
| *CENPE* | 0.317 | 1.374 | 1.239 | 1.523 | < 0.001 | < 0.001 |
| *USP30* | 0.786 | 2.194 | 1.697 | 2.838 | < 0.001 | < 0.001 |
| *FGD3* | −0.170 | 0.844 | 0.798 | 0.892 | < 0.001 | < 0.001 |
| *ESPL1* | 0.551 | 1.735 | 1.447 | 2.081 | < 0.001 | < 0.001 |
| *OMD* | −0.239 | 0.787 | 0.727 | 0.853 | < 0.001 | < 0.001 |
| *S100P* | 0.086 | 1.090 | 1.059 | 1.122 | < 0.001 | < 0.001 |
| *CENPL* | 0.437 | 1.548 | 1.338 | 1.791 | < 0.001 | < 0.001 |
| *GSTM3* | −0.188 | 0.829 | 0.778 | 0.882 | < 0.001 | < 0.001 |
| *ZWINT* | 0.285 | 1.329 | 1.208 | 1.462 | < 0.001 | < 0.001 |
| *PTTG3P* | 0.217 | 1.242 | 1.155 | 1.335 | < 0.001 | < 0.001 |
| *STAT5B* | −0.428 | 0.652 | 0.565 | 0.752 | < 0.001 | < 0.001 |
| *LSR* | 0.396 | 1.485 | 1.301 | 1.696 | < 0.001 | < 0.001 |
| *AURKB* | 0.228 | 1.256 | 1.163 | 1.356 | < 0.001 | < 0.001 |
| *BCL2* | −0.190 | 0.827 | 0.776 | 0.882 | < 0.001 | < 0.001 |
| *RALGAPB* | 0.482 | 1.619 | 1.377 | 1.904 | < 0.001 | < 0.001 |
| *DYNLRB2* | −0.245 | 0.782 | 0.720 | 0.850 | < 0.001 | < 0.001 |
| *UTP23* | 0.392 | 1.481 | 1.297 | 1.691 | < 0.001 | < 0.001 |
| *GSTK1* | −0.394 | 0.674 | 0.590 | 0.770 | < 0.001 | < 0.001 |
| *FANCD2* | 0.341 | 1.407 | 1.253 | 1.579 | < 0.001 | < 0.001 |
| *GPI* | 0.335 | 1.398 | 1.248 | 1.566 | < 0.001 | < 0.001 |

*Note*: METABRIC, the Molecular Taxonomy of Breast Cancer International Consortium; HR, hazard ratio; LCI, lower limit of confidence interval; UCI, upper limit of confidence interval.
