# Supplementary material for: CBioProfiler: A Web and Standalone Pipeline for Cancer Biomarker and Subtype Characterization
Source: Genomics Proteomics Bioinformatics. 2024 Jun 12;22(3):qzae045. doi: 10.1093/gpbjnl/qzae045 (PMC11464420; doi:10.1093/gpbjnl/qzae045)
Supplement: qzae045_Supplementary_Data [file qzae045_supplementary_data.zip › Table S4-done.docx]

**Table S4 Differences between ABAT high expression group and ABAT low expression group**

| **Variable** | **ABAT high expression levels** | | ***P* value** |
| --- | --- | --- | --- |
|  | **High expression**  **(N = 1068)** | **Low expression**  **(N = 1068)** |  |
| **ER** |  |  |  |
| Negative | 46 (4.3%) | 394 (36.9%) | < 0.001 |
| Positive | 984 (92.1%) | 524 (49.1%) | |
| Missing | 38 (3.6%) | 150 (14.0%) | |
| **Tumor size** |  |  |  |
| Mean (SD) | 2.53 (1.36) | 2.72 (1.72) | 0.00536 |
| Median [Min, Max] | 2.20 [0,18.0] | 2.30 [0,18.2] | |
| Missing | 30 (2.8%) | 134 (12.5%) | |
| **Node status** |  |  |  |
| Mean (SD) | 0.444 (0.497) | 0.513 (0.500) | 0.00207 |
| Median [Min, Max] | 0 [0,1.00] | 1.00 [0,1.00] | |
| Missing | 19 (1.8%) | 125 (11.7%) | |
| **Age (years)** |  |  |  |
| Mean (SD) | 63.2 (12.5) | 59.0 (13.2) | < 0.001 |
| Median [Min, Max] | 64.3 [26.4,92.1] | 59.8 [21.9,96.3] | |
| Missing | 2 (0.2%) | 11 (1.0%) |  |
| **Grade** |  |  |  |
| Mean (SD) | 2.20 (0.657) | 2.64 (0.556) | < 0.001 |
| Median [Min, Max] | 2.00 [1.00,3.00] | 3.00 [1.00,3.00] | |
| Missing | 75 (7.0%) | 159 (14.9%) | |

*Note*: ER, estrogen receptor; SD, standard deviation; ABAT, 4-aminobutyrate aminotransferase.
