# Supplementary material for: CBioProfiler: A Web and Standalone Pipeline for Cancer Biomarker and Subtype Characterization
Source: Genomics Proteomics Bioinformatics. 2024 Jun 12;22(3):qzae045. doi: 10.1093/gpbjnl/qzae045 (PMC11464420; doi:10.1093/gpbjnl/qzae045)
Supplement: qzae045_Supplementary_Data [file qzae045_supplementary_data.zip › Table S7-done.docx]

**Table S7 Comparison of clinical features in the GSE31210 cohort (training set)**

| **Variable** | **Subtype 1** | **Subtype 2** | **Subtype 3** | **Subtype 4** | ***P* value** |
| --- | --- | --- | --- | --- | --- |
|  | **(N = 86)** | **(N = 40)** | **(N = 70)** | **(N = 30)** |  |
| **Age (years)** |  |  |  |  |  |
| Mean (SD) | 59.0 (8.05) | 60.0 (7.17) | 60.8 (6.29) | 58.0 (8.05) | 0.28 |
| Median [Min, Max] | 60.0 [30.0,76.0] | 61.0 [38.0,71.0] | 61.0 [35.0,72.0] | 59.0 [39.0,69.0] |  |
| **Cluster** |  |  |  |  |  |
| Cluster 1 | 24 (27.9%) | 23 (57.5%) | 0 (0%) | 0 (0%) | < 0.001 |
| Cluster 2 | 8 (9.3%) | 0 (0%) | 20 (28.6%) | 4 (13.3%) |  |
| Missing | 54 (62.8%) | 17 (42.5%) | 50 (71.4%) | 26 (86.7%) |  |
| **Gender** |  |  |  |  |  |
| Female | 48 (55.8%) | 11 (27.5%) | 43 (61.4%) | 19 (63.3%) | 0.00292 |
| Male | 38 (44.2%) | 29 (72.5%) | 27 (38.6%) | 11 (36.7%) |  |
| **MYC** |  |  |  |  |  |
| High | 4 (4.7%) | 4 (10.0%) | 3 (4.3%) | 6 (20.0%) | 0.127 |
| Low | 81 (94.2%) | 36 (90.0%) | 66 (94.3%) | 24 (80.0%) |  |
| ND | 1 (1.2%) | 0 (0%) | 1 (1.4%) | 0 (0%) |  |
| **Pathological stage** | | |  |  |  |
| IA | 35 (40.7%) | 8 (20.0%) | 47 (67.1%) | 24 (80.0%) | < 0.001 |
| IB | 17 (19.8%) | 13 (32.5%) | 20 (28.6%) | 4 (13.3%) |  |
| II | 34 (39.5%) | 19 (47.5%) | 3 (4.3%) | 2 (6.7%) |  |
| **Gene alteration status** | |  |  |  |  |
| ALK-fusion+ | 9 (10.5%) | 2 (5.0%) | 0 (0%) | 0 (0%) | < 0.001 |
| EGFR mutation+ | 43 (50.0%) | 10 (25.0%) | 49 (70.0%) | 25 (83.3%) |  |
| EGFR/KRAS/ALK- | 23 (26.7%) | 21 (52.5%) | 20 (28.6%) | 4 (13.3%) |  |
| KRAS mutation+ | 11 (12.8%) | 7 (17.5%) | 1 (1.4%) | 1 (3.3%) |  |
| **Smoking status** |  |  |  |  |  |
| Ever-smoker | 44 (51.2%) | 31 (77.5%) | 22 (31.4%) | 14 (46.7%) | < 0.001 |
| Never-smoker | 42 (48.8%) | 9 (22.5%) | 48 (68.6%) | 16 (53.3%) |  |

*Note*: MYC, Myelocytomatosis; ND, not determined.
