# Supplementary material for: CBioProfiler: A Web and Standalone Pipeline for Cancer Biomarker and Subtype Characterization
Source: Genomics Proteomics Bioinformatics. 2024 Jun 12;22(3):qzae045. doi: 10.1093/gpbjnl/qzae045 (PMC11464420; doi:10.1093/gpbjnl/qzae045)
Supplement: qzae045_Supplementary_Data [file qzae045_supplementary_data.zip › Table S8-done.docx]

**Table S8 Comparison of clinical features in the TCGA-LUAD cohort (validation set)**

| **Variable** | **Subtype 1** | **Subtype 2** | **Subtype 3** | **Subtype 4** | ***P* value** |
| --- | --- | --- | --- | --- | --- |
|  | **(N = 7)** | **(N = 375)** | **(N = 84)** | **(N = 31)** |  |
| **Age (years)** |  |  |  |  |  |
| Mean (SD) | 66.6 (8.54) | 64.7 (10.3) | 67.3 (9.36) | 66.3 (8.74) | 0.181 |
| Median [Min, Max] | 70.0 [56.0,78.0] | 65.0 [33.0,88.0] | 67.0 [41.0,86.0] | 67.0 [48.0,80.0] |  |
| Missing | 0 (0%) | 7 (1.9%) | 3 (3.6%) | 0 (0%) |  |
| **Gender** |  |  |  |  |  |
| Female | 3 (42.9%) | 190 (50.7%) | 54 (64.3%) | 22 (71.0%) | 0.0268 |
| Male | 4 (57.1%) | 185 (49.3%) | 30 (35.7%) | 9 (29.0%) |  |
| **Ethnicity** |  |  |  |  |  |
| Not Hispanic or Latino | 5 (71.4%) | 278 (74.1%) | 70 (83.3%) | 25 (80.6%) | 0.859 |
| Hispanic or Latino | 0 (0%) | 6 (1.6%) | 1 (1.2%) | 0 (0%) |  |
| Missing | 2 (28.6%) | 91 (24.3%) | 13 (15.5%) | 6 (19.4%) |  |
| **Race** |  |  |  |  |  |
| Black or African American | 1 (14.3%) | 38 (10.1%) | 8 (9.5%) | 4 (12.9%) | 0.681 |
| White | 5 (71.4%) | 286 (76.3%) | 70 (83.3%) | 23 (74.2%) |  |
| American Indian or Alaska native | 0 (0%) | 1 (0.3%) | 0 (0%) | 0 (0%) |  |
| Asian | 0 (0%) | 4 (1.1%) | 1 (1.2%) | 2 (6.5%) |  |
| Missing | 1 (14.3%) | 46 (12.3%) | 5 (6.0%) | 2 (6.5%) |  |
| **Number pack years smoked** | |  |  |  |  |
| Mean (SD) | 42.1 (13.2) | 44.4 (28.4) | 34.9 (23.1) | 30.0 (15.5) | 0.0218 |
| Median [Min, Max] | 40.3 [28.0,60.0] | 40.0 [1.00,154] | 30.0 [0.150,100] | 35.0 [4.50,50.0] |  |
| Missing | 3 (42.9%) | 114 (30.4%) | 27 (32.1%) | 12 (38.7%) |  |
| **Tobacco smoking history** | | |  |  |  |
| Mean (SD) | 2.57 (0.976) | 2.84 (1.10) | 2.75 (1.01) | 2.77 (1.06) | 0.852 |
| Median [Min, Max] | 3.00 [1.00,4.00] | 3.00 [1.00,5.00] | 3.00 [1.00,4.00] | 3.00 [1.00,4.00] |  |
| Missing | 0 (0%) | 11 (2.9%) | 3 (3.6%) | 0 (0%) |  |
| **Pathologic stage** |  |  |  |  |  |
| Stage I | 3 (42.9%) | 186 (49.6%) | 59 (70.2%) | 19 (61.3%) | 0.0131 |
| Stage II | 2 (28.6%) | 97 (25.9%) | 9 (10.7%) | 10 (32.3%) |  |
| Stage III | 1 (14.3%) | 68 (18.1%) | 9 (10.7%) | 2 (6.5%) |  |
| Stage IV | 1 (14.3%) | 20 (5.3%) | 4 (4.8%) | 0 (0%) |  |
| Missing | 0 (0%) | 4 (1.1%) | 3 (3.6%) | 0 (0%) |  |
