# Supplementary figures and images for: CBioProfiler: A Web and Standalone Pipeline for Cancer Biomarker and Subtype Characterization
Source: Genomics Proteomics Bioinformatics. 2024 Jun 12;22(3):qzae045. doi: 10.1093/gpbjnl/qzae045 (PMC11464420; doi:10.1093/gpbjnl/qzae045)

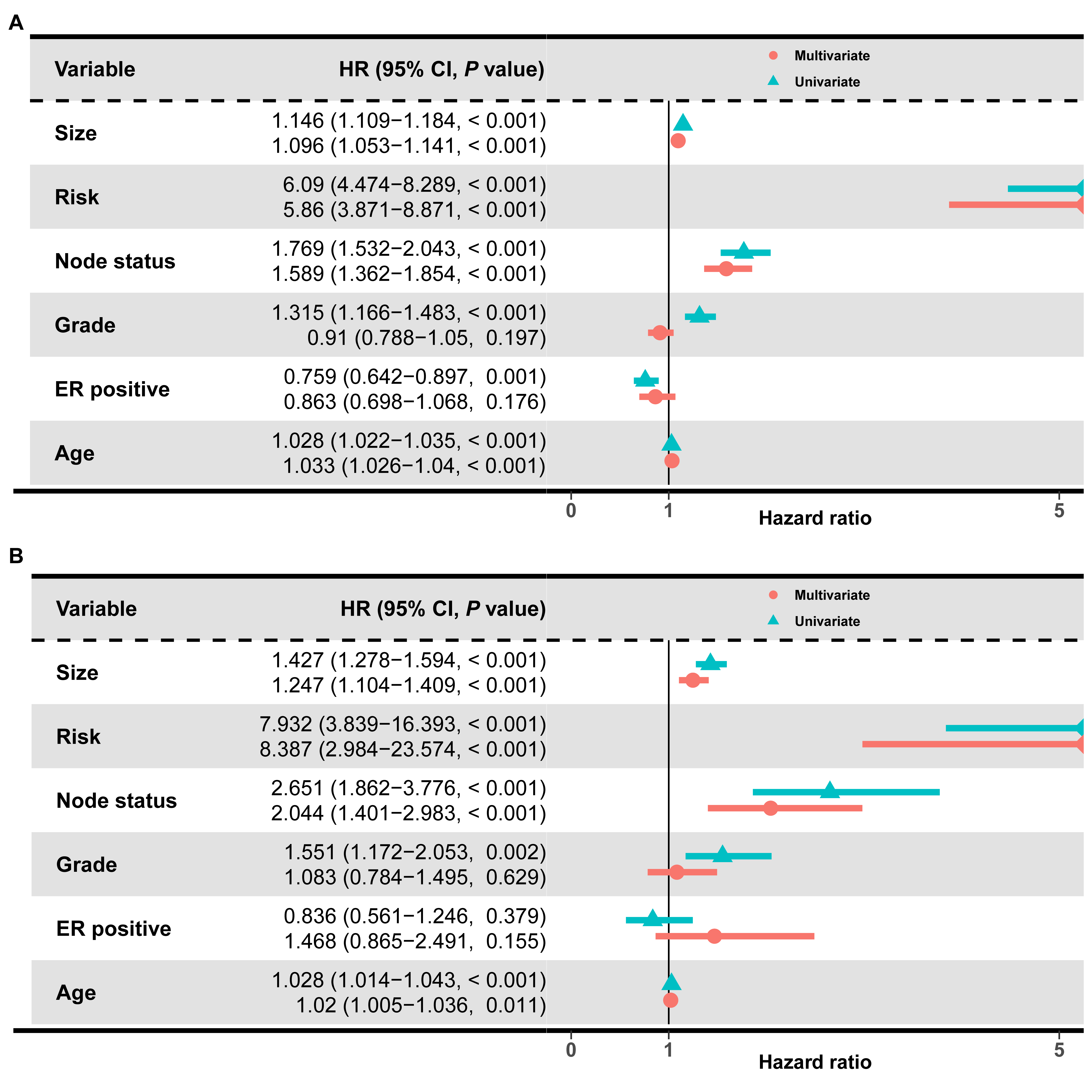

Supplement: qzae045_Supplementary_Data [file qzae045_supplementary_data.zip › Figure S6.tif]

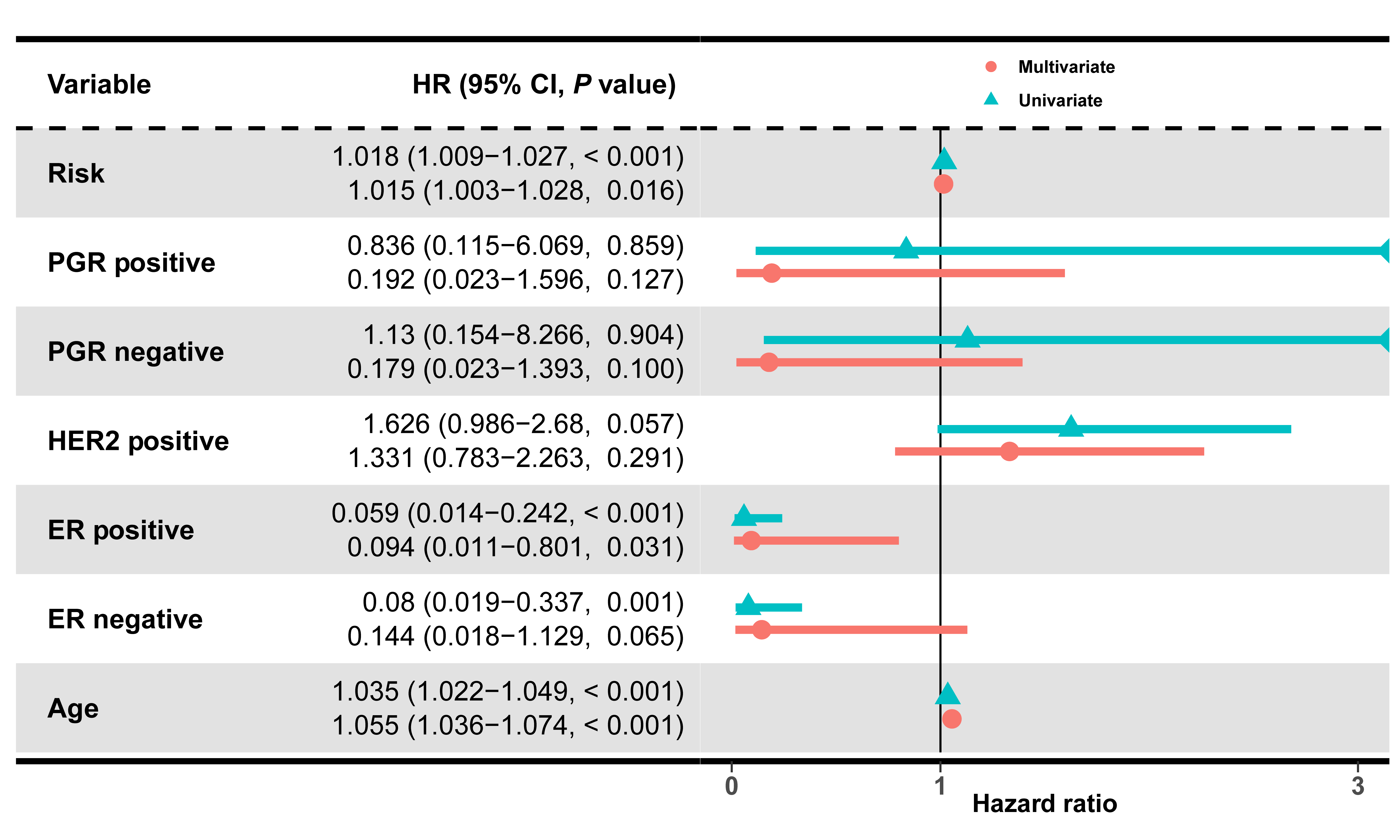

Supplement: qzae045_Supplementary_Data [file qzae045_supplementary_data.zip › Figure S7.tif]

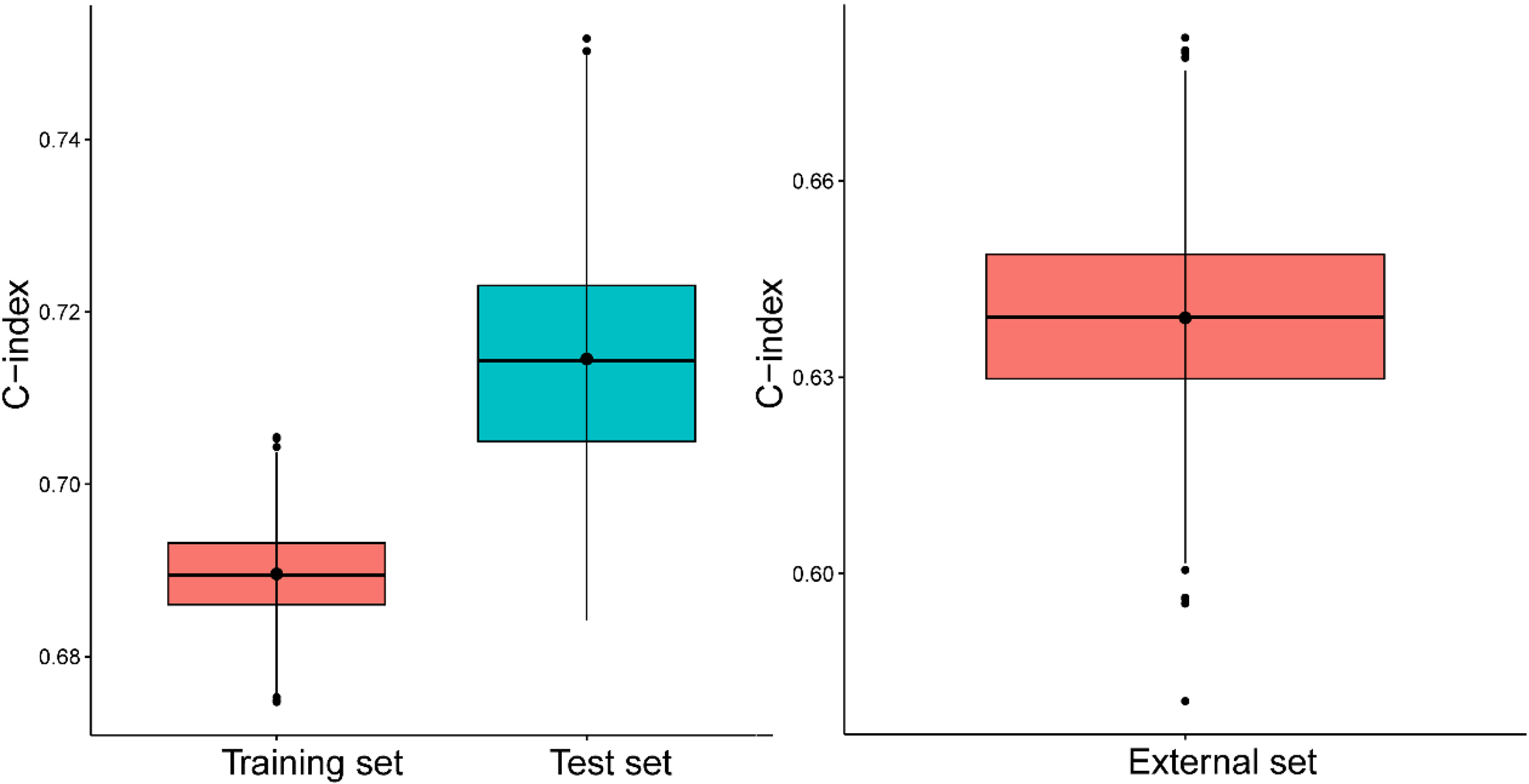

Supplement: qzae045_Supplementary_Data [file qzae045_supplementary_data.zip › Figure S8.tif]

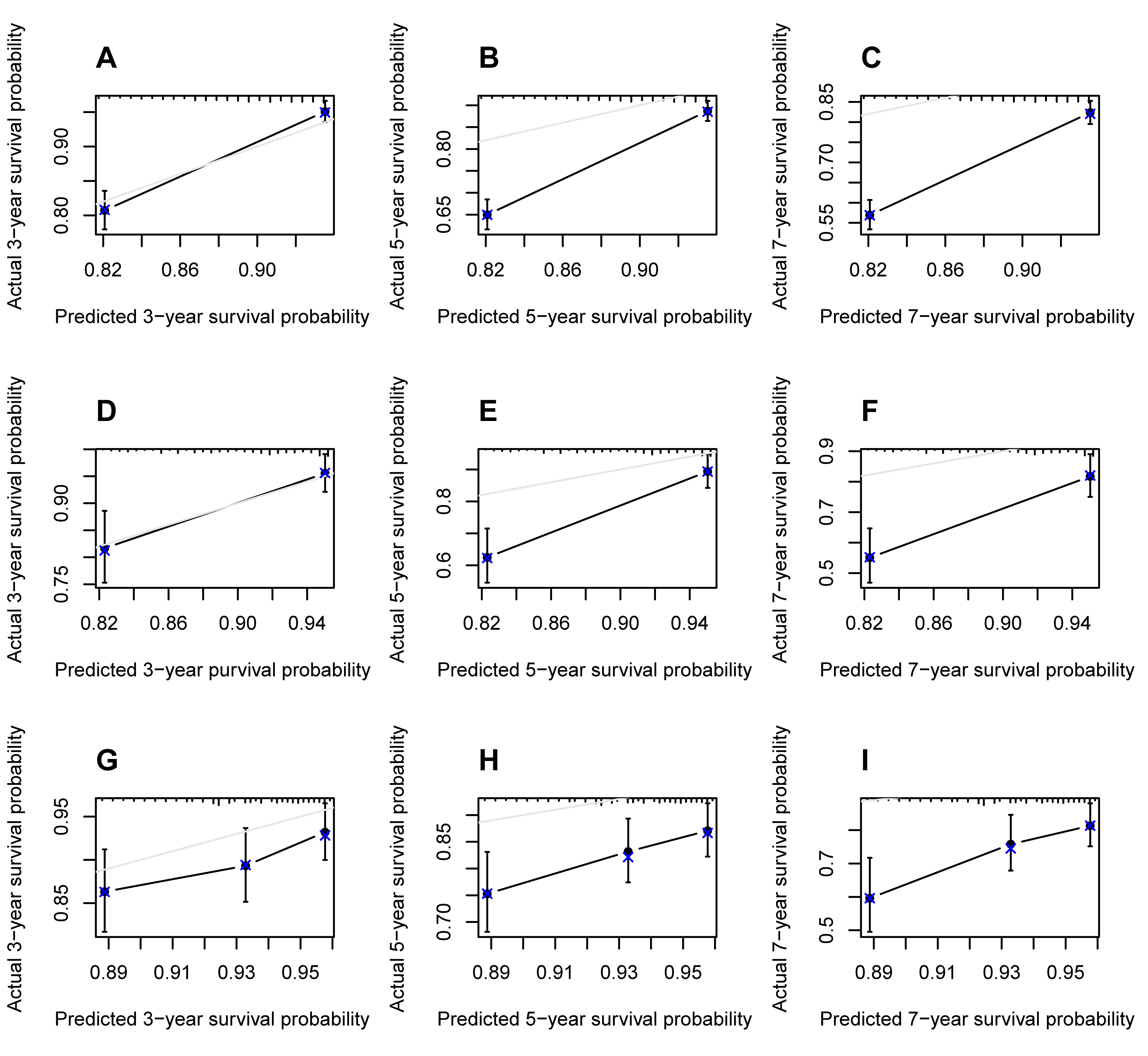

Supplement: qzae045_Supplementary_Data [file qzae045_supplementary_data.zip › Figure S9.tif]

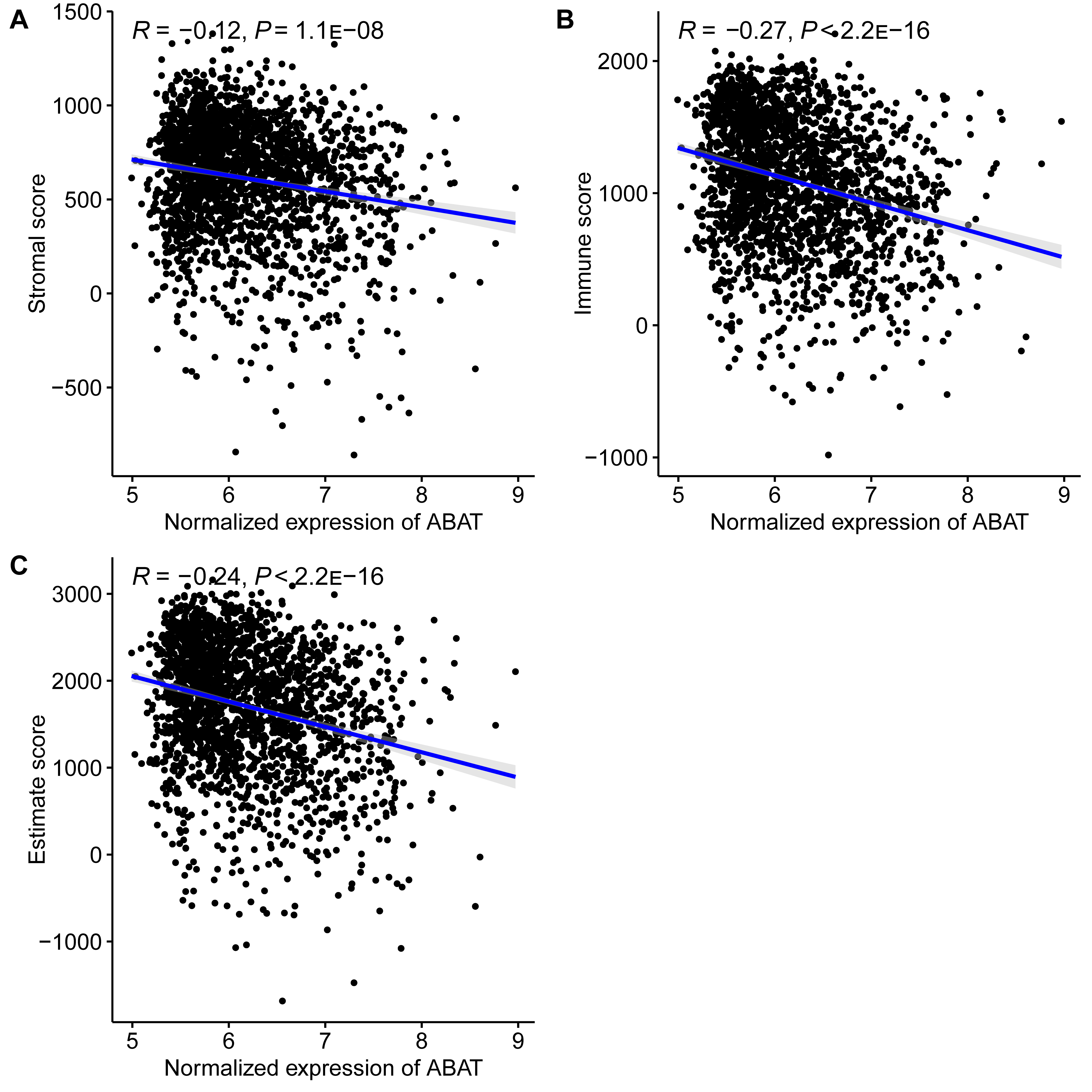

Supplement: qzae045_Supplementary_Data [file qzae045_supplementary_data.zip › Figure S11.tif]

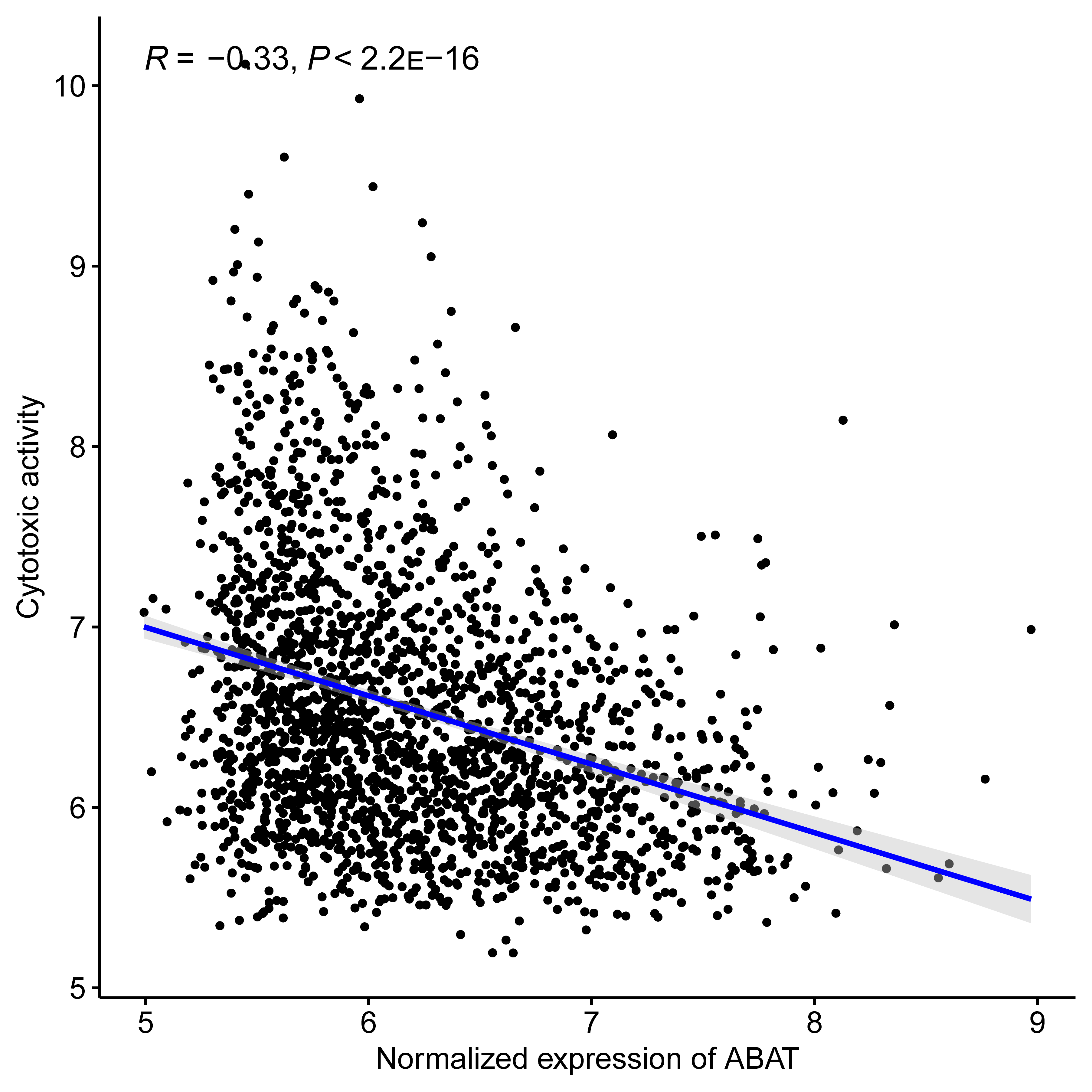

Supplement: qzae045_Supplementary_Data [file qzae045_supplementary_data.zip › Figure S12.tif]

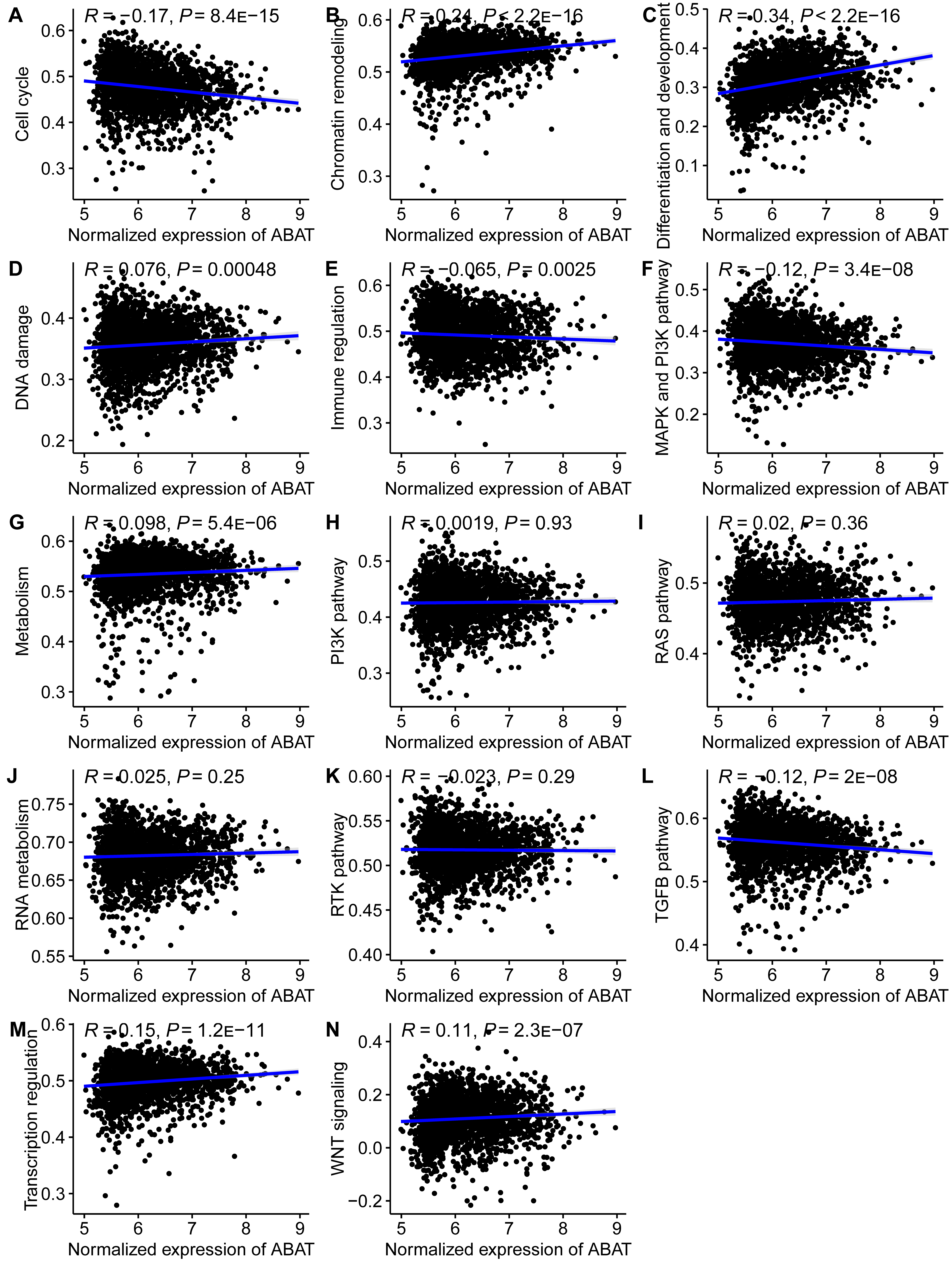

Supplement: qzae045_Supplementary_Data [file qzae045_supplementary_data.zip › Figure S13.tif]

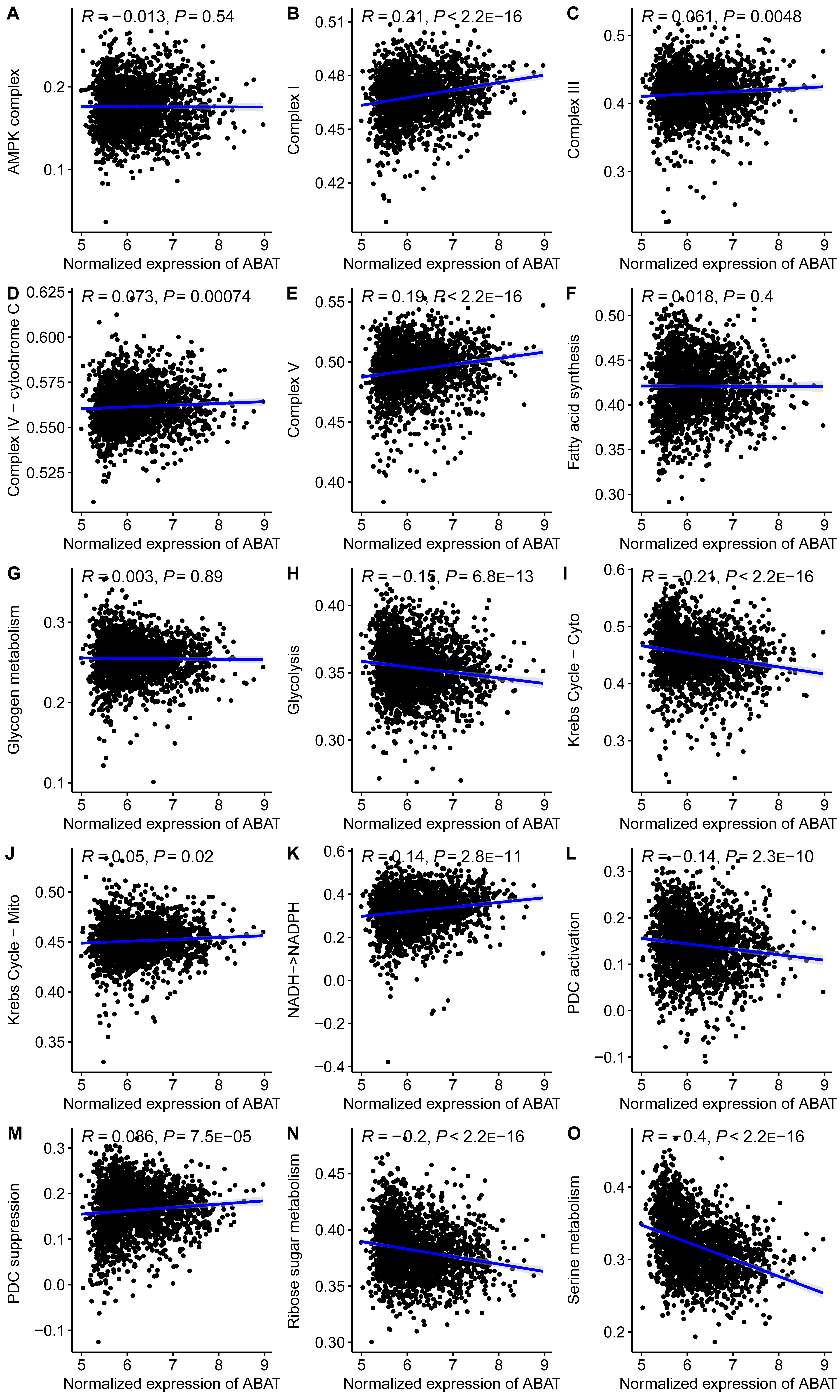

Supplement: qzae045_Supplementary_Data [file qzae045_supplementary_data.zip › Figure S15.tif]

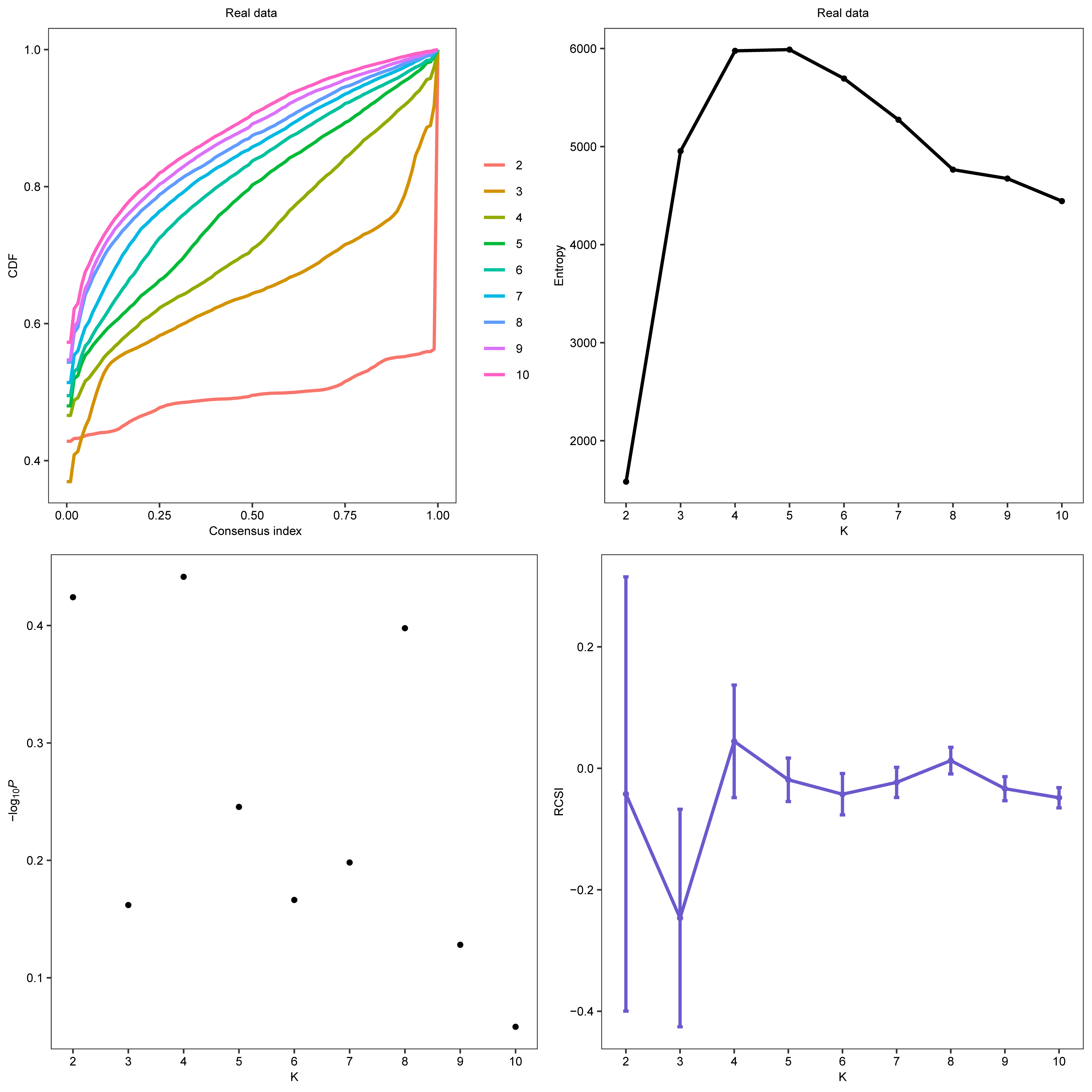

Supplement: qzae045_Supplementary_Data [file qzae045_supplementary_data.zip › Figure S16.tif]

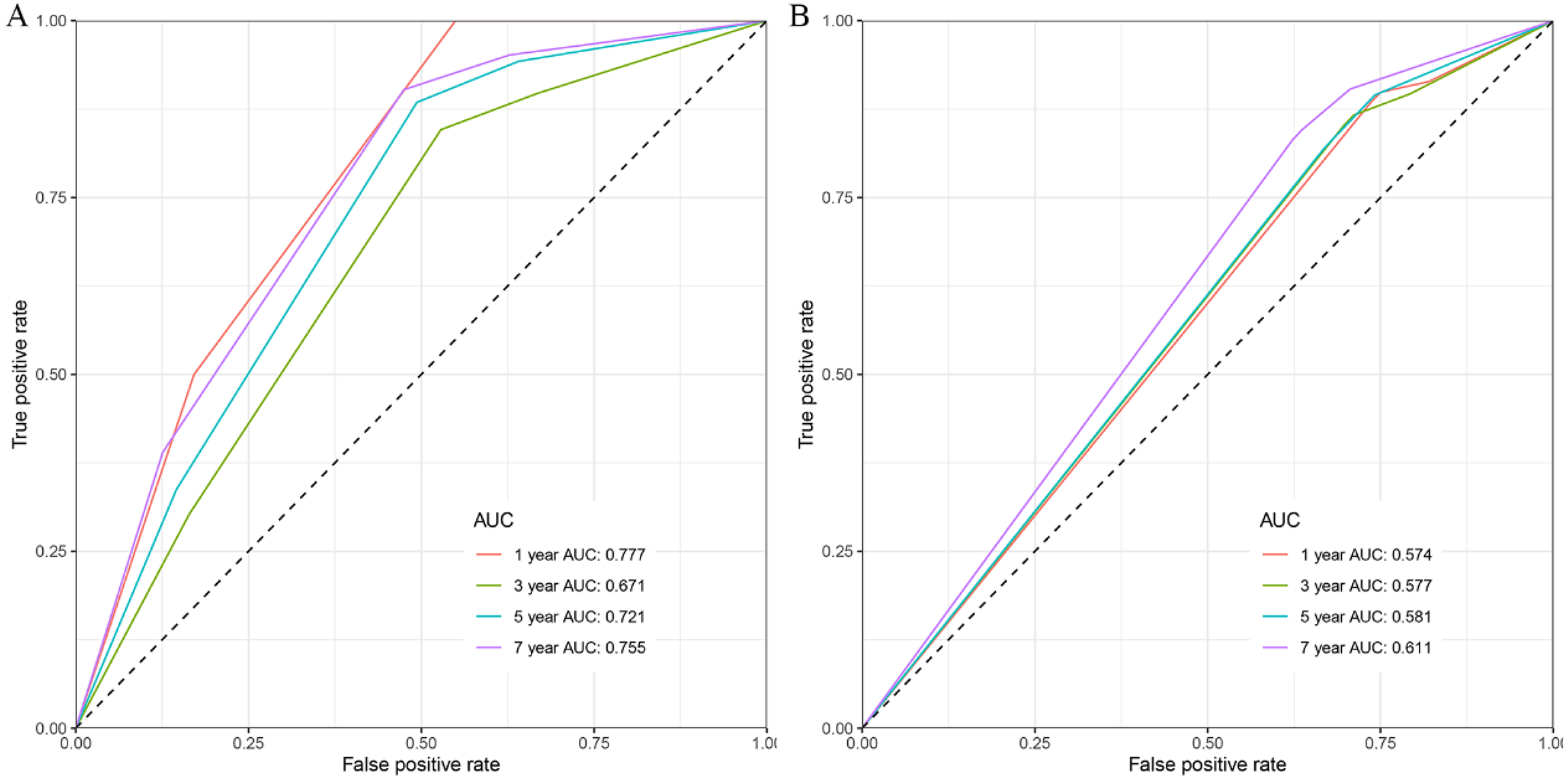

Supplement: qzae045_Supplementary_Data [file qzae045_supplementary_data.zip › Figure S17.tif]

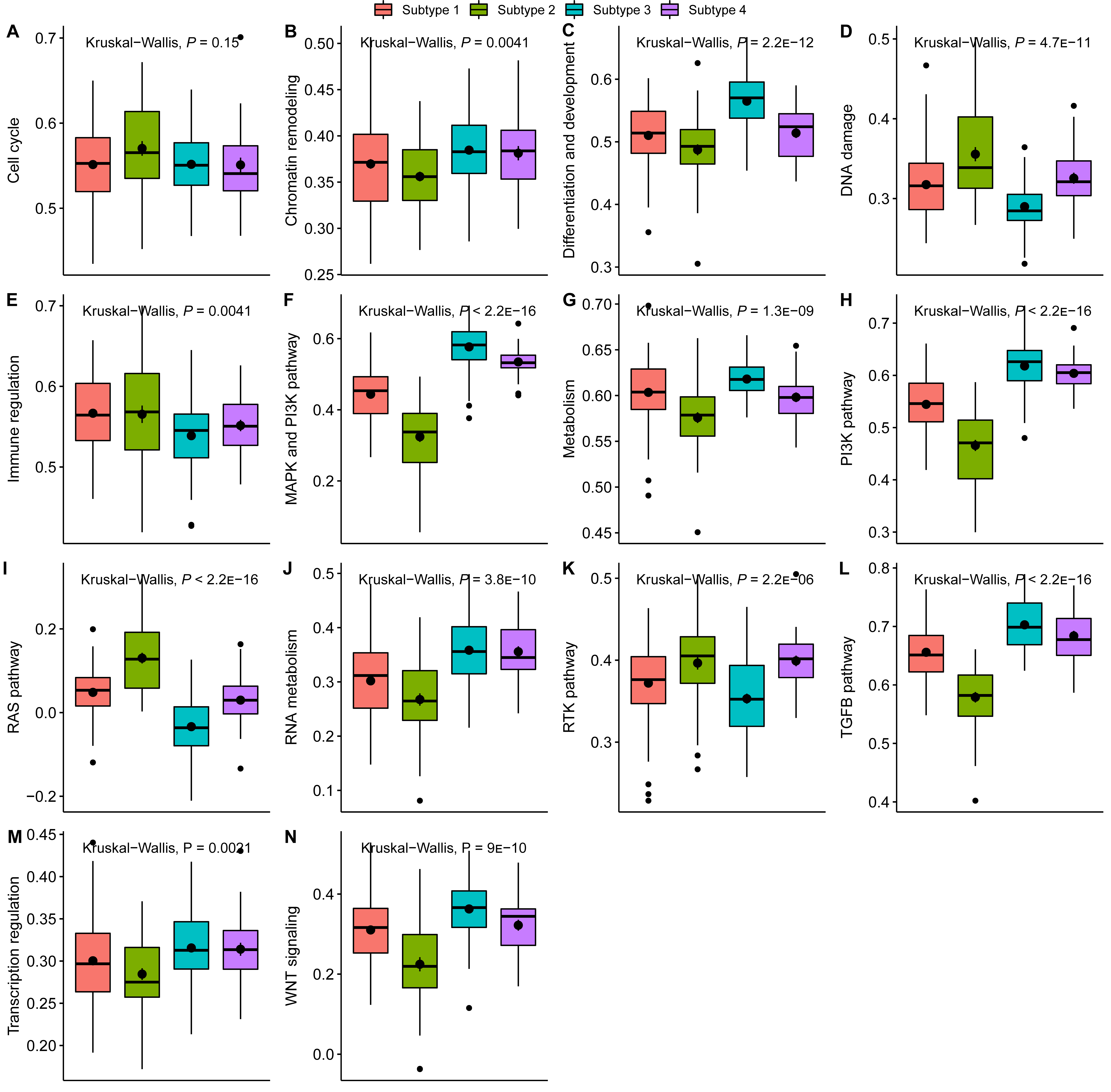

Supplement: qzae045_Supplementary_Data [file qzae045_supplementary_data.zip › Figure S18.tif]

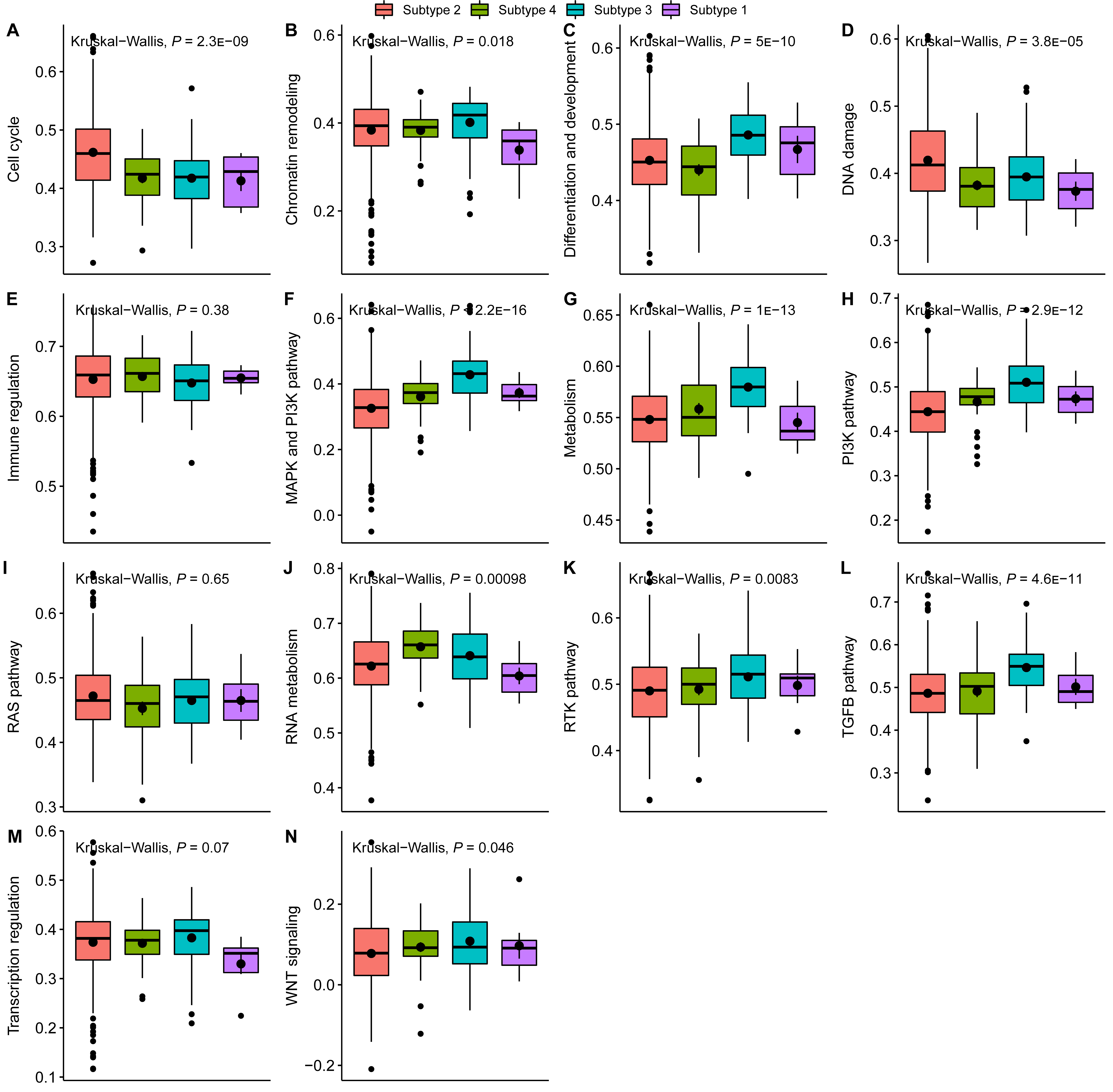

Supplement: qzae045_Supplementary_Data [file qzae045_supplementary_data.zip › Figure S19.tif]

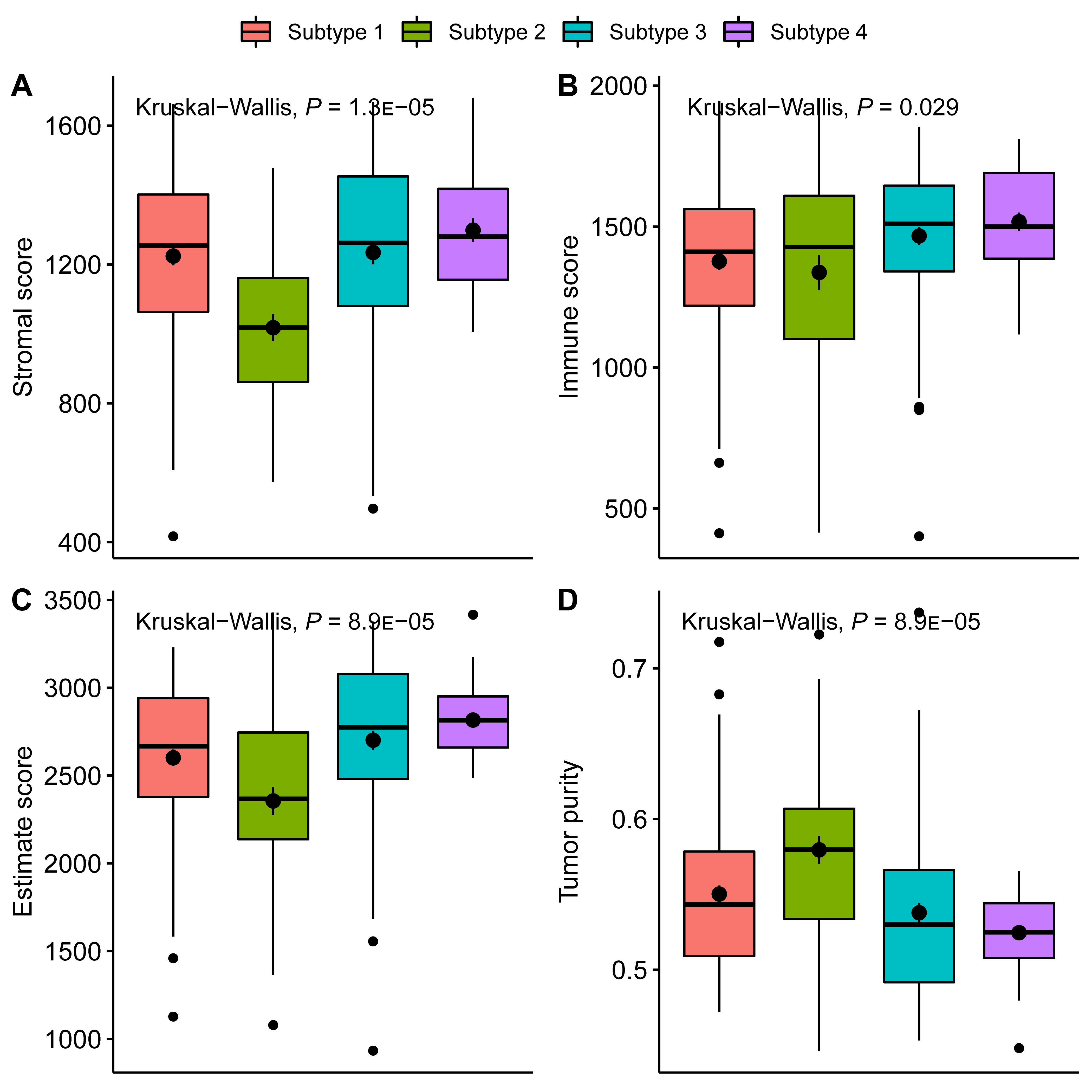

Supplement: qzae045_Supplementary_Data [file qzae045_supplementary_data.zip › Figure S20.tif]

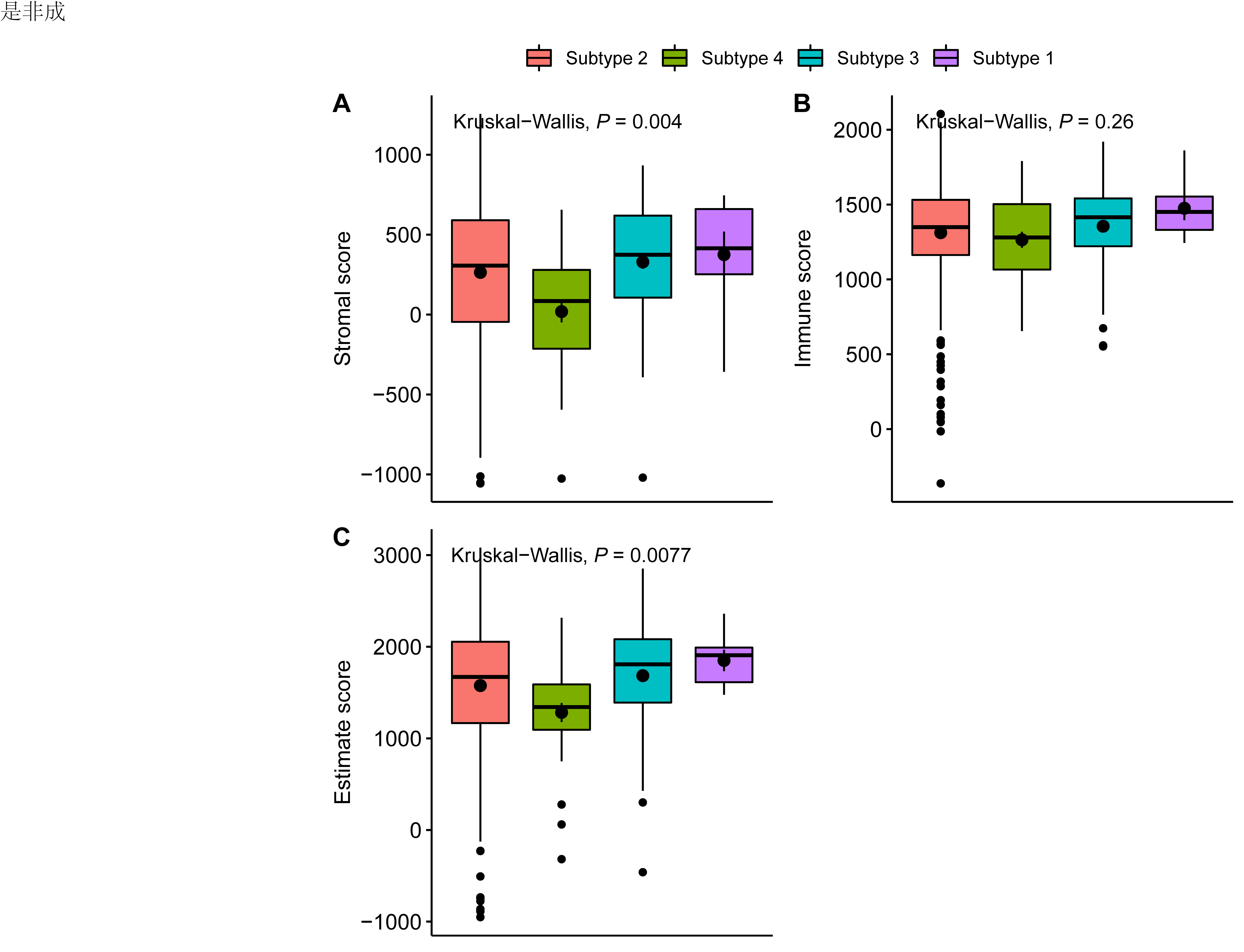

Supplement: qzae045_Supplementary_Data [file qzae045_supplementary_data.zip › Figure S21.tif]

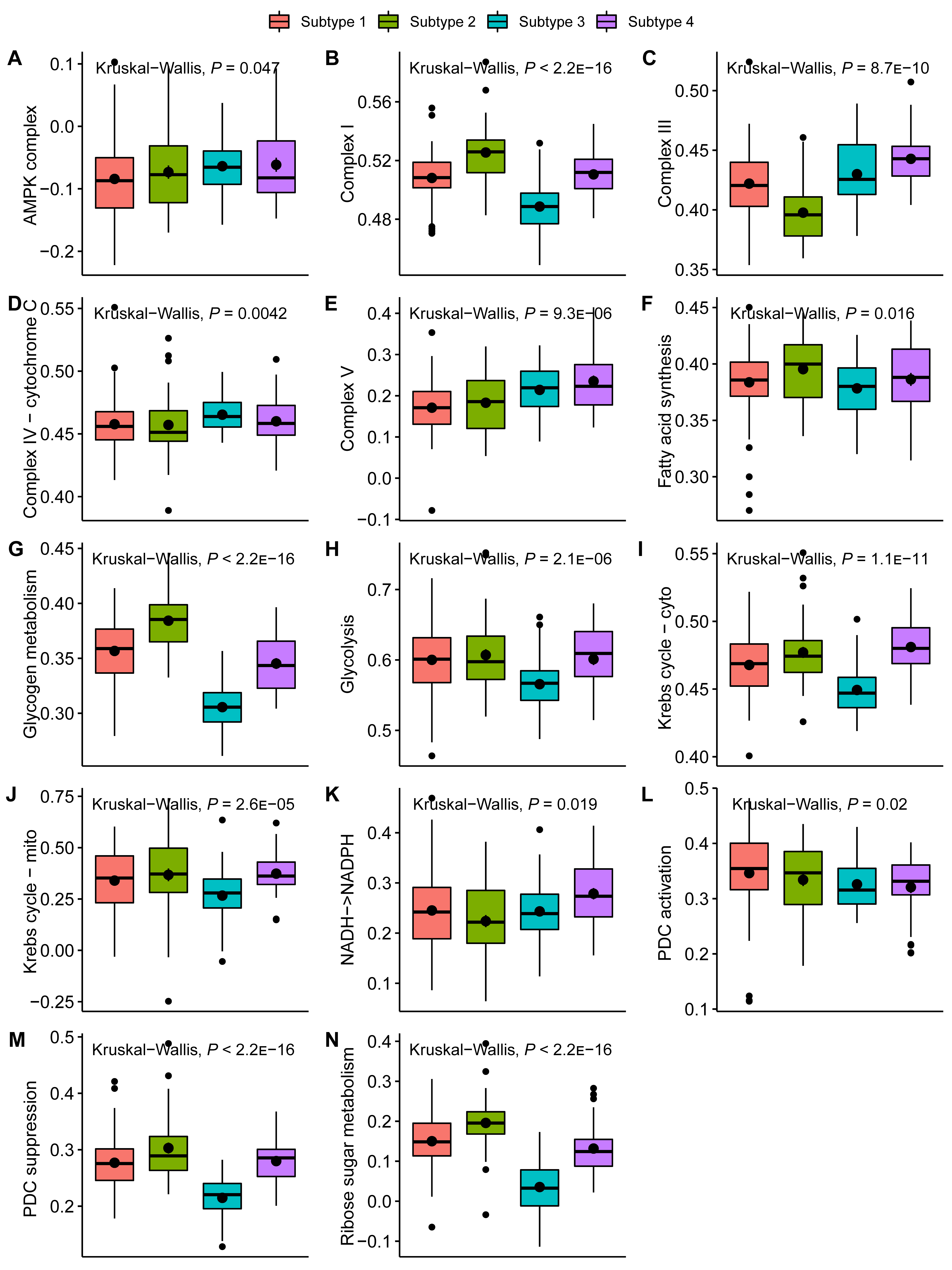

Supplement: qzae045_Supplementary_Data [file qzae045_supplementary_data.zip › Figure S28.tif]

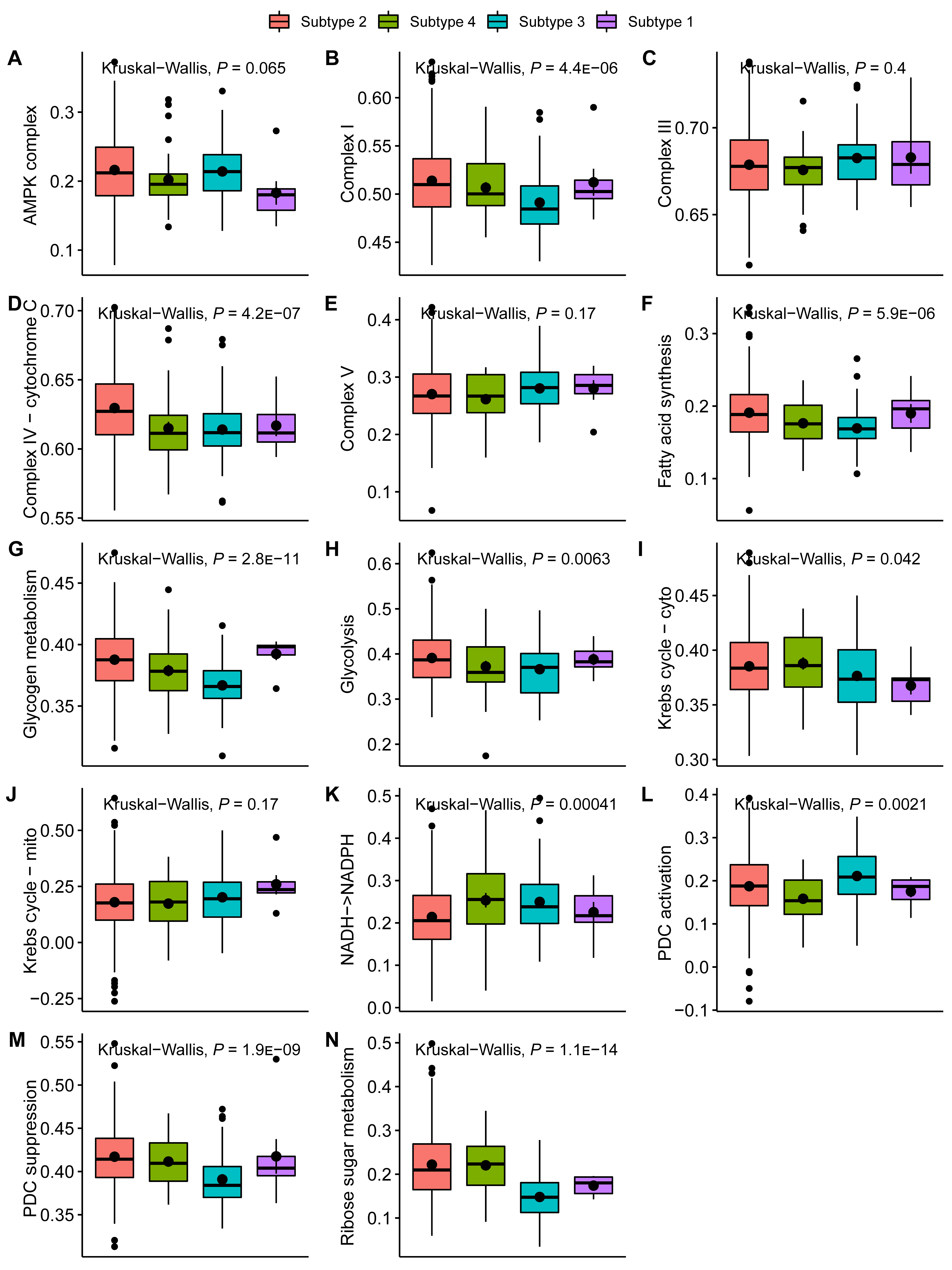

Supplement: qzae045_Supplementary_Data [file qzae045_supplementary_data.zip › Figure S29.tif]

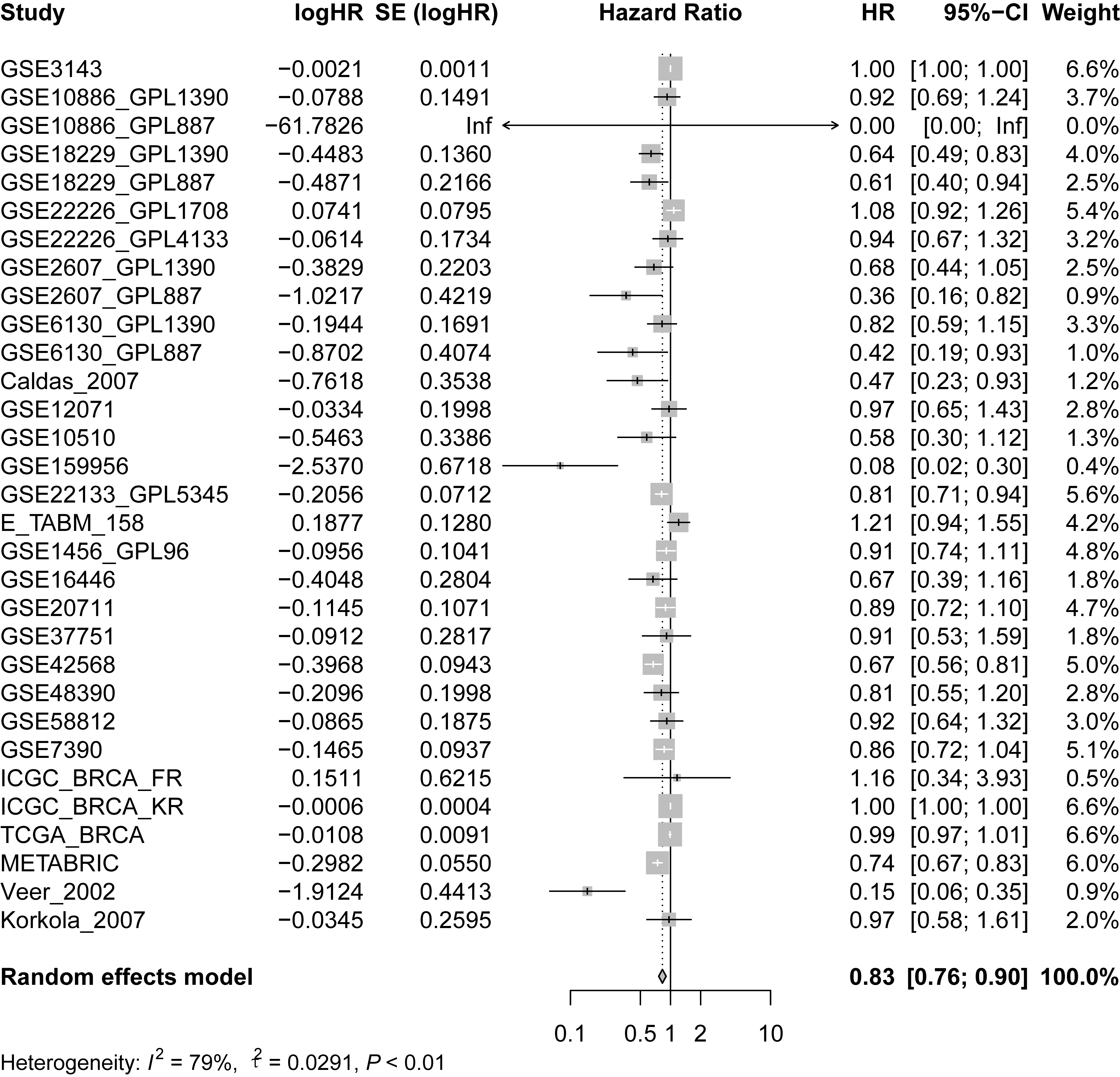

Supplement: qzae045_Supplementary_Data [file qzae045_supplementary_data.zip › Figure S30.tif]

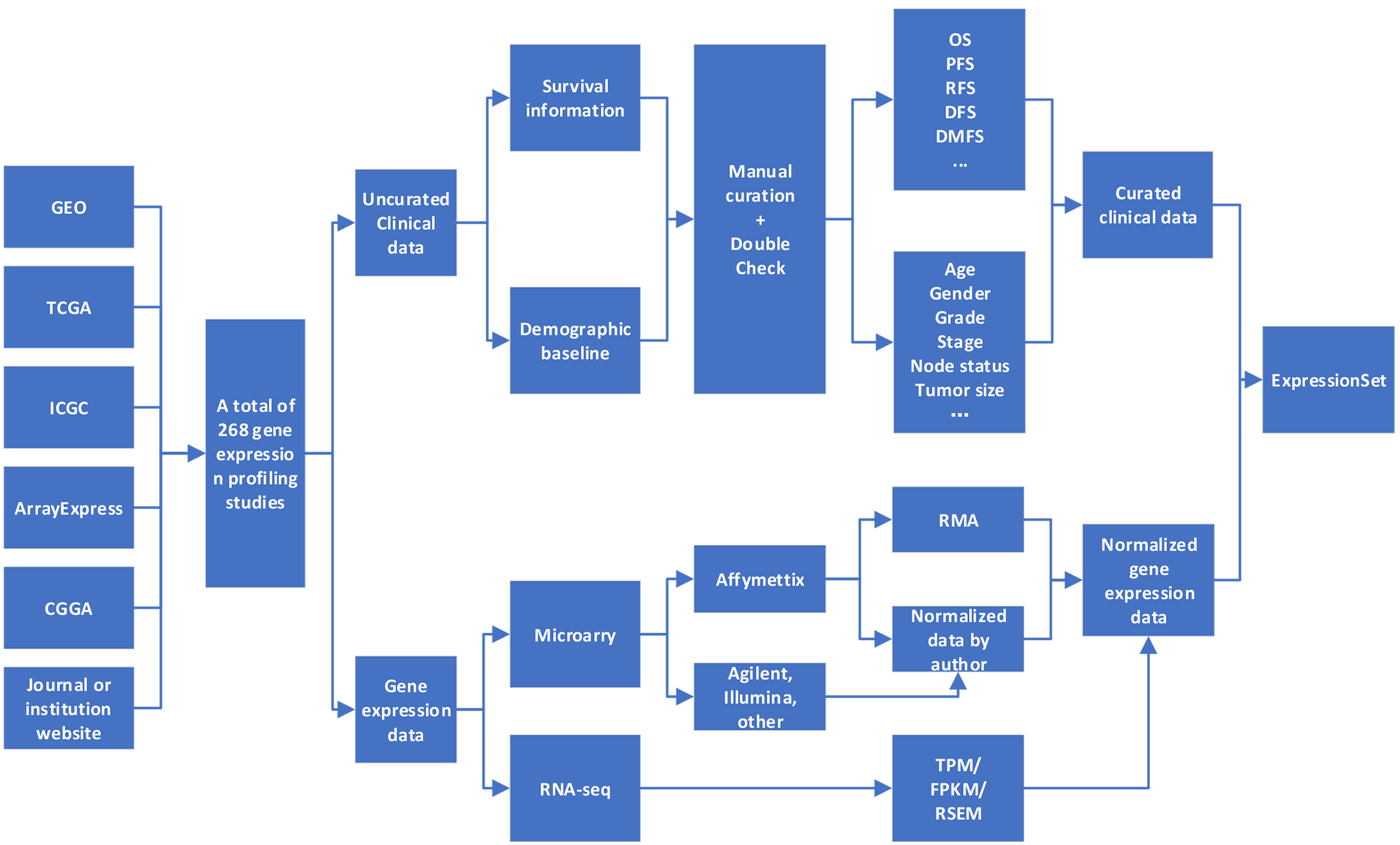

Supplement: qzae045_Supplementary_Data [file qzae045_supplementary_data.zip › Figure S1.tif]

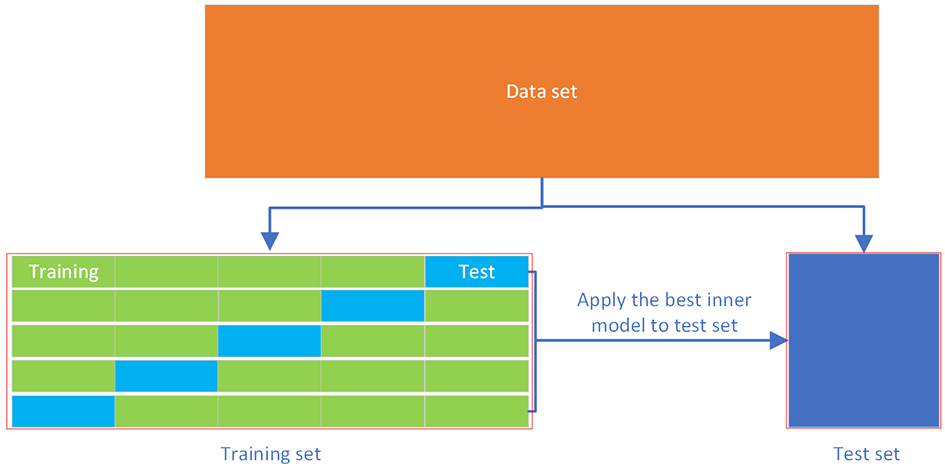

Supplement: qzae045_Supplementary_Data [file qzae045_supplementary_data.zip › Figure S2.tif]

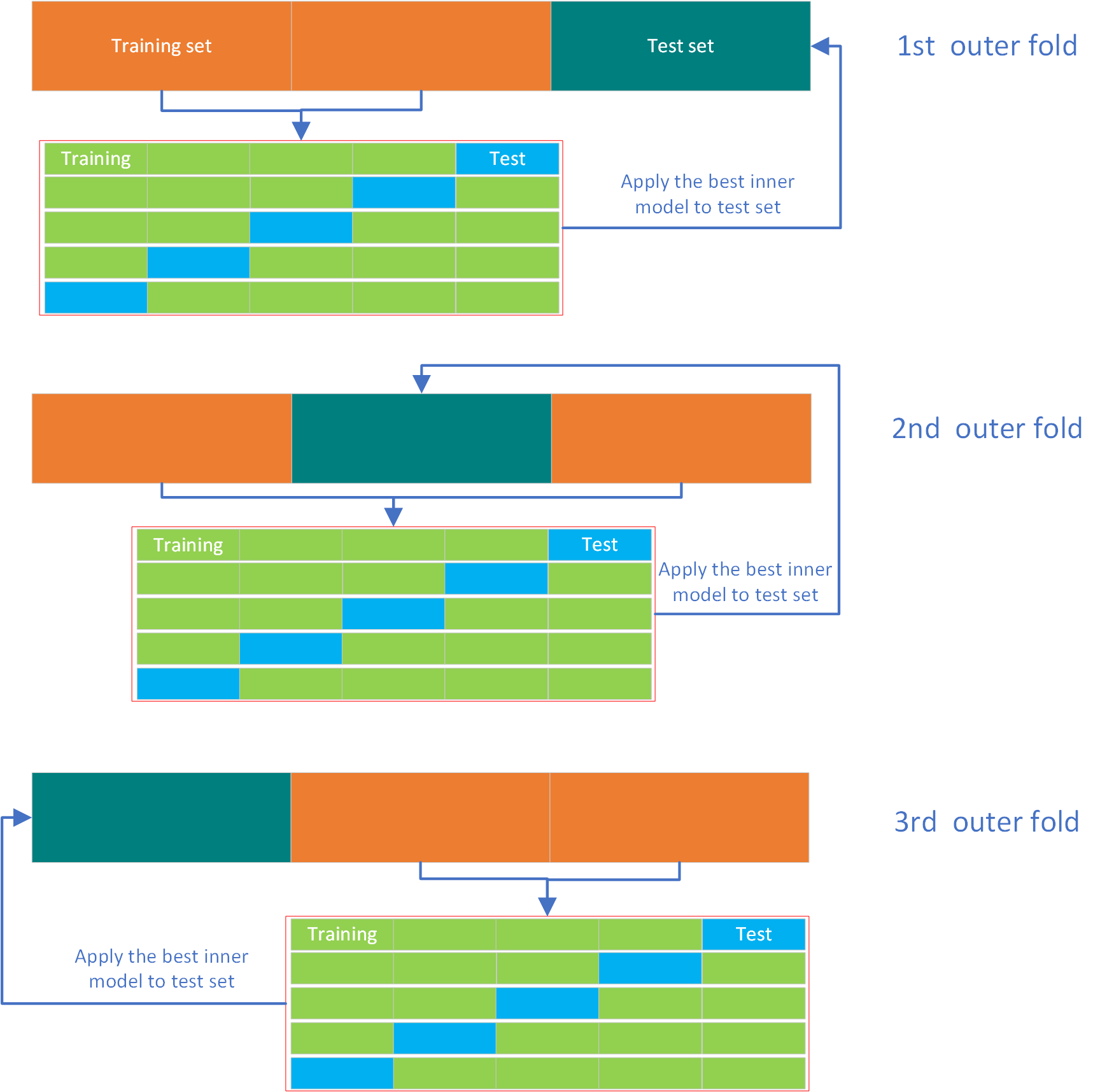

Supplement: qzae045_Supplementary_Data [file qzae045_supplementary_data.zip › Figure S3.tif]

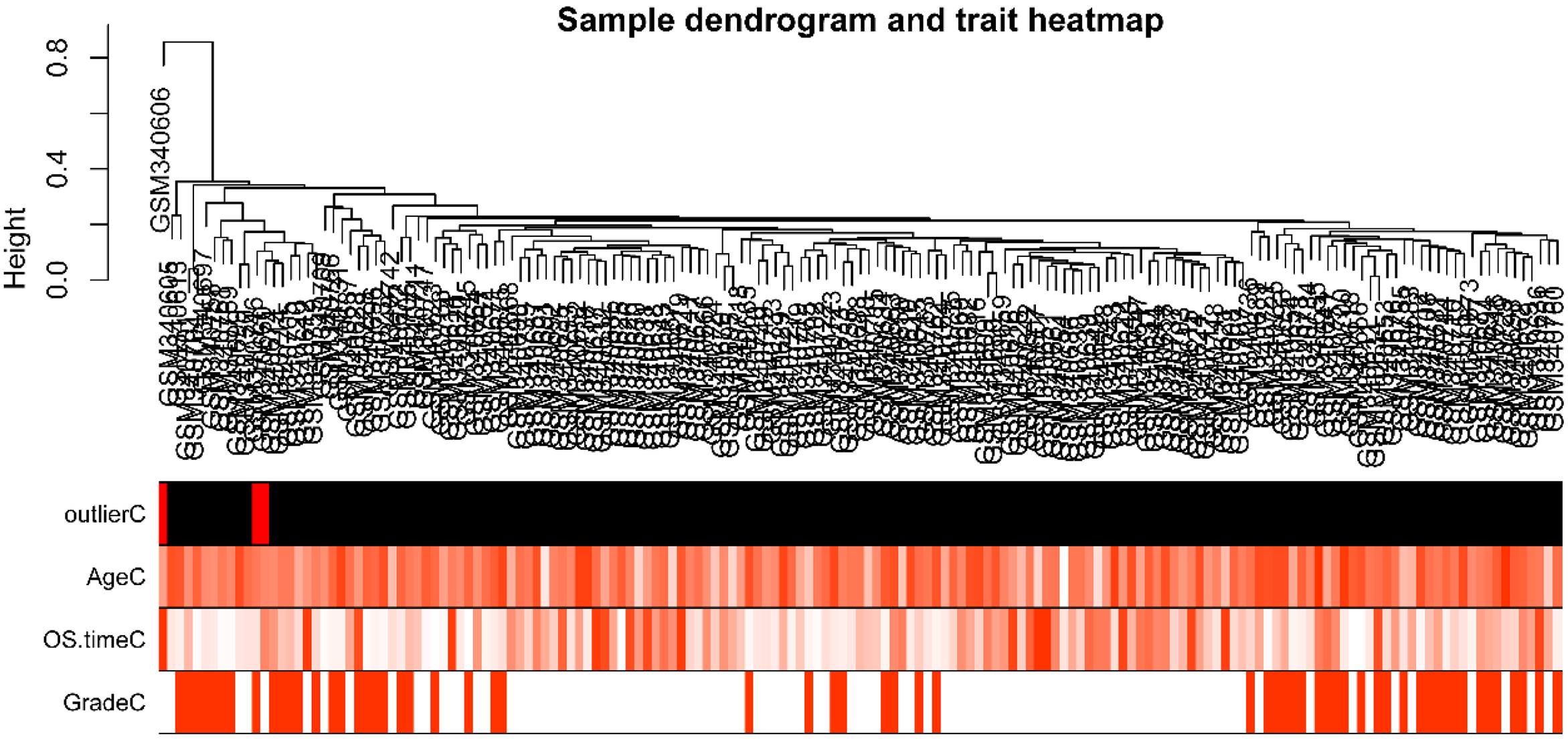

Supplement: qzae045_Supplementary_Data [file qzae045_supplementary_data.zip › Figure S4.tif]

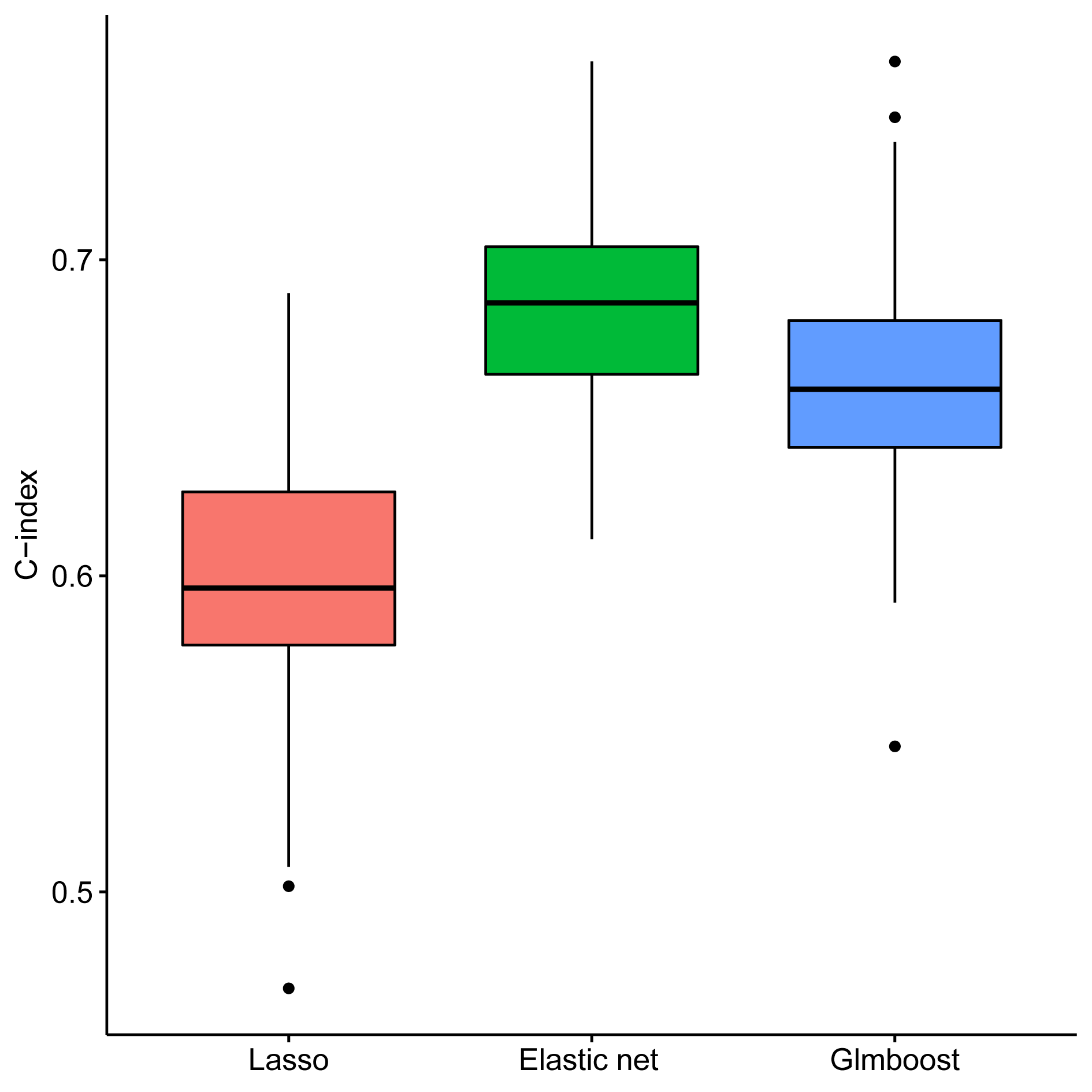

Supplement: qzae045_Supplementary_Data [file qzae045_supplementary_data.zip › Figure S5.tif]
